# Screening helper T lymphocyte epitopes based on IFN-γ/IL-10 ratio for developing a novel multi-epitope vaccine candidate using *Wolbachia* surface protein as an adjuvant against visceral leishmaniasis

**DOI:** 10.1186/s13071-025-06756-5

**Published:** 2025-03-25

**Authors:** Jianhui Zhang, Tianhang Lv, Shuoyan Tan, Lingqi Yu, Yangjian Chi, Jianping Chen, Xiaohui Fan, Xiaoyan Lu

**Affiliations:** 1 Pharmaceutical Informatics Institute, College of Pharmaceutical Sciences, Zhejiang University, Hangzhou, 310058 China; 2 State Key Laboratory of Chinese Medicine Modernization, Innovation Center of Yangtze River Delta, Zhejiang University, Jiaxing, 314100 China; 3 Department of Pathogenic Biology, West China School of Basic Medical Sciences and Forensic Medicine, Sichuan University, Chengdu, 610041 China; 4 Department of Urinary Surgery, Jianou Municipal Hospital of Fujian Province, Jiaou, 353199 China

**Keywords:** *Leishmania*, Multi-epitope vaccine, Immunoinformatics, *Wolbachia* surface protein, Adjuvant

## Abstract

**Background:**

Visceral leishmaniasis (VL) is the most lethal form of leishmaniasis. In terms of anti-leishmanial vaccines, favorable immune responses are Th1 responses that primarily produce interferon gamma (IFN-γ) and activate macrophages for leishmanicidal effects. The selection of IFN-γ-inducing epitopes in silico may reduce the steps of pre-clinical evaluation and increase the certainty of the better-designed vaccine. *Wolbachia* surface protein (WSP) derived from *Wolbachia* bacteria that have been reported to reside in sandflies can trigger TLR2 and TLR4 activation to favor Th1 immune responses, which may serve as a potential adjuvant candidate for the *Leishmania* vaccine. Therefore, in this study, helper T lymphocyte epitopes that may induce favorable immune responses were identified, and WSP was served as an adjuvant to design a novel multi-epitope vaccine candidate.

**Methods:**

*Leishmania* hemoglobin receptor (HbR), kinetoplastid membrane protein-11 (KMP-11), glycoprotein of 63 kDa (Gp63), thiol-specific antioxidant antigen (TSA), and sterol 24-c-methyltransferase (SMT) were analyzed by immunoinformatics to screen helper T lymphocyte and cytotoxic T lymphocyte epitopes. The antigenicity, toxicity, allergenicity, and IFN-γ-inducing epitope potential of T epitopes were predicted. The immune simulation was performed to calculate IFN-γ/interleukin (IL)-10 ratios to predict the immune responses induced by the helper T lymphocyte epitopes. Molecular docking and molecular dynamics simulations were carried out to analyze the interactions and stability of the docked complexes. The immune simulation of a multi-epitope vaccine candidate was carried out to predict its immunogenicity.

**Results:**

Some helper T lymphocyte epitopes that were predicted with the potential of inducing Th1 responses and cytotoxic T lymphocyte epitopes were selected to develop a novel multi-epitope vaccine candidate with WSP as an adjuvant. It was found in molecular docking and interaction analysis that TLR2 and TLR4 can recognize WSP, supporting the potential of adjuvant for the *Leishmania* vaccine. The results from immune simulation demonstrated that the multi-epitope vaccine candidate induced obvious cytokine (IFN-γ, IL-12, and IL-2) secretion and Th1 as well as memory T cell production, similar to that of Leish-111f.

**Conclusions:**

Our vaccine candidate may interact with TLR2 and TLR4 and exhibit good immunogenicity, favoring *Leishmania* clearance. Our strategy provides a novel multi-epitope vaccine candidate and references for other vaccine developments.

**Graphical Abstract:**

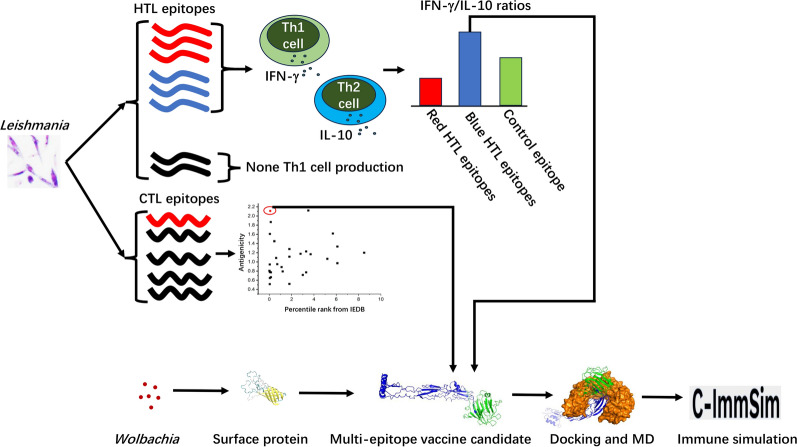

**Supplementary Information:**

The online version contains supplementary material available at 10.1186/s13071-025-06756-5.

## Background

Visceral leishmaniasis (VL) is caused by *Leishmania donovani* (*L. donovani*) and *Leishmania infantum* (*L. infantum*) parasites through transmission mediated by sandflies. VL, which is characterized by irregular bouts of fever, weight loss, and hepatosplenomegaly, is fatal if untreated [1]. Currently, available treatments for VL rely on chemotherapies, but these drugs are accompanied by toxicity, expensive costs, and drug resistance [2]. People in the former Soviet Union, Central Asia, and Iran were vaccinated with live parasites (leishmanization) to prevent *Leishmania* infection [3, 4]. In addition, most individuals recovering from VL develop immune protection against *Leishmania* and become resistant to later reinfection for a long time [4, 5], which demonstrates that immunity against leishmaniasis and the development of anti-*Leishmania* vaccines are feasible.

Multi-epitope vaccines are characterized by their specific composition and safety. These vaccines are equipped with heterovalent protection, allow the coverage of natural pathogen antigen diversity, match the variability of major histocompatibility complex (MHC) alleles, and reduce the risk of pathogen escape [6, 7]. Various epitopes from different *Leishmania* proteins were employed to develop a multi-epitope vaccine against the parasites in our study. *Leishmania* hemoglobin receptor (HbR), kinetoplastid membrane protein-11 (KMP-11), conserved surface glycoprotein of 63 kDa (Gp63), thiol-specific antioxidant antigen (TSA), and sterol 24-c-methyltransferase (SMT) have been shown to trigger appropriate Th1 responses for parasitic clearance, which indicates that these antigens could offer desirable epitopes recognized by T cells. In addition, HbR, KMP-11, Gp63, TSA, and SMT are conserved across *Leishmania* species and are considered as suitable antigens because of their cross-protective efficacy against different *Leishmania* species [4, 8–11]. Consequently, helper T lymphocyte (HTL) and cytotoxic T lymphocyte (CTL) epitopes from HbR, KMP-11, Gp63, TSA, and SMT were screened for the construction of a multi-epitope vaccine.

Th1 responses are vital to interferon gamma (IFN-γ) secretion and are considered to play significant roles in *Leishmania* clearance, whereas Th2 responses favor interleukin (IL)−10 production, suppress Th1 responses, and support parasite survival [12]. The ratio of IFN-γ/IL-10 has been employed to assess the balance between Th1 and Th2 responses to evaluate the types of immune response induced by antigens [8]. In our computational strategy for multi-epitope vaccine development, the Th1 cell generation and IFN-γ/IL-10 ratio of HTL epitopes were predicted to select the epitopes that may trigger Th1 immune responses. Synthetic antigens such as epitope vaccines are insufficiently immunogenic, and adjuvants can induce robust immune responses to enhance the efficacy of weak antigens [13]. Natural infection mediated by sandflies may confer hosts with desirable protection against *Leishmania*. *Leishmania* transmission involves microbiota from sandflies when natural infection occurs [14]. Therefore, the components of microorganisms from sandflies may be suitable adjuvants. *Wolbachia* surface protein (WSP) from *Wolbachia* bacteria, which have been reported to reside in sandflies, can trigger TLR2 and TLR4 activation to promote Th1 response formation [13, 15–18]. It has been found that WSP as an adjuvant supports IFN-γ and IL-2 production, impedes Th2 responses, and decreases *Brugia malayi* burdens [19]. In brief, WSP may serve as a suitable adjuvant for *Leishmania* vaccines.

In summary, the HTL epitopes from HbR, KMP-11, Gp63, TSA, and SMT, with a high IFN-γ/IL-10 ratio, were selected to construct a multi-epitope vaccine candidate with WSP as an adjuvant. The designed vaccine candidate was subsequently used for further analysis, including molecular docking, molecular dynamics simulation, and immune simulation. Our strategy of vaccine design provides a methodological basis for the development of leishmaniasis and other infectious disease vaccines.

## Methods

**Fig. 1:**
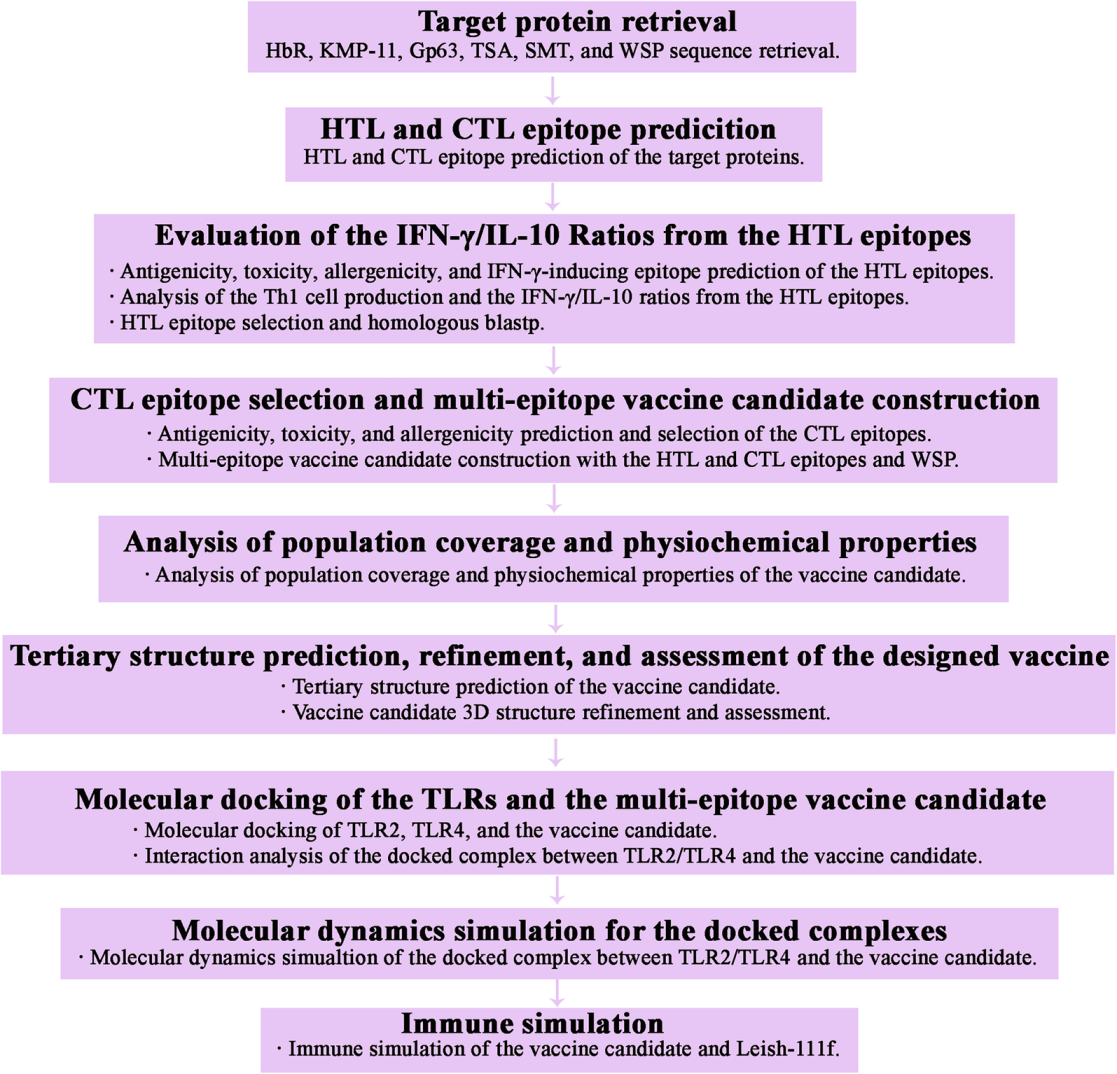
Workflow for the multi-epitope vaccine candidate development

Multiple bioinformatics tools and approaches were employed to develop a multi-epitope vaccine candidate. Antigenicity, toxicity, allergenicity, IFN-γ-inducing epitope potential, epitope percentile rank, Th1 cell production potential, the IFN-γ/IL-10 ratio, and non-homology with human and mouse proteins are the criteria of HTL and CTL epitope selection. The workflow of our study is shown in Fig. 1.

### Target protein retrieval

Hemoglobin receptor (HbR; accession: AAT72300.1), kinetoplastid membrane protein-11 (KMP-11; accession: CAJ1993035.1), *Leishmania* conserved surface glycoprotein of 63 kDa (Gp63; accession: CAD42816.1), thiol-specific antioxidant antigen (TSA; accession: ABX11567.1), and sterol 24-c-methyltransferase (SMT; accession: ADX32472.1) were retrieved from the National Center for Biotechnology Information (NCBI) (https://www.ncbi.nlm.nih.gov). *Wolbachia* surface protein (WSP; accession: AJ252062.1) was used as an adjuvant. PEPCK_335–351_, the peptide (335–351 amino acid (AA)) of *Leishmania* glycosomal phosphoenolpyruvate carboxykinase, was used as a control for the analysis of the IFN-γ/IL-10 ratio. Leish-111f, which has been assessed in clinical trials, was used as a control for in silico immune stimulations. The sequences of PEPCK_335–351_ and Leish-111f were obtained from publications with PMIDs 26491077 and 12213399, respectively [20, 21].

### HTL and CTL epitope prediction

HbR, KMP-11, Gp63, TSA, and SMT amino acid sequence fasta files were submitted to the Immune Epitope Database server (IEDB; http://tools.iedb.org/, 85% accuracy) for the HTL and CTL epitope prediction using NetMHCIIPan 4.1 EL (for HTL epitopes) and NetMHCPan 4.1 EL (for CTL epitopes) algorithms. A reference set of 27 human leukocyte antigen II (HLA II) alleles, covering 99% of population, was chosen to predict 15-mer HTL epitopes [22]. A reference set of HLA I alleles, prepared by the IEDB and covering 97% of population, was selected to predict 9- or 10-mer CTL epitopes [22]. Epitopes with percentile ranks < 10 were considered potential binding epitopes.

### Ratio of IFN-γ to IL-10 from HTL epitope evaluation

First, the antigenicity, toxicity, allergenicity, and IFN-γ-inducing epitope potential of the HTL epitopes from HbR, KMP-11, Gp63, TSA, and SMT were analyzed. Antigenicity was evaluated using the VaxiJen server (https://www.ddg-pharmfac.net/vaxijen/VaxiJen/VaxiJen.html). Evaluation of toxicity was performed with the ToxinRred2 (https://webs.iiitd.edu.in/raghava/toxinpred2/index.html). Allergenicity identification was performed via the AllerTOP2 (https://www.ddg-pharmfac.net/AllerTOP/index.html). IFN-γ-inducing epitopes was identified by the IFNepitope server (https://webs.iiitd.edu.in/raghava/ifnepitope/predict.php).

In addition, nontoxic and nonallergenic HTL epitopes with IFN-γ-inducing epitope potential and an antigenicity index > 0.5 were submitted to the C-ImmSim server for immune simulations (https://kraken.iac.rm.cnr.it/C-IMMSIM/index.php). Three doses of the HTL epitopes and PEPCK_335–351_ without any adjuvants were administered at an interval of 2 weeks. Th1 cells from C-ImmSim were analyzed, the HTL epitopes that failed to induce Th1 cells were excluded. The levels of IFN-γ and IL-10 on the 5th, 30th, and 40th day after immunization were subjected to calculate the IFN-γ/IL-10 ratios. HTL epitopes that had higher log10(IFN-γ/IL-10) values on the 5th, 30th, and 40th day after immunization than those of PEPCK_335–351_ simultaneously were selected for homologous protein analysis.

### Multi-epitope vaccine candidate construction

Nontoxic and nonallergenic CTL epitopes were selected on the basis of their high antigenicity indices and low percentile ranks. The similarity of the selected epitopes and WSP to humans and mice was identified using Blastp alignment on the NCBI platform. Epitopes with sequence identity < 30% or an expected value (E value) > 1e-5 cutoff were considered nonhomologous with human and mouse proteins and taken to construct multi-epitope vaccine candidate. *Wolbachia* surface protein (WSP) was utilized as an adjuvant linked to the N-terminal of vaccine candidate by an EAAK linker. AAY and GPGPG linkers merged the CTL and HTL epitopes, respectively. Finally, a 6 × His-tag was attached to the C-terminal of the vaccine candidate for future detection and purification of recombinant protein.

### Analysis of population coverage and physiochemical properties

Input texts containing HLA alleles of HTL or CTL epitopes from the designed vaccine were prepared and submitted to the IEDB population coverage server (http://tools.iedb.org/population/). Diverse regions (such as the world, East Asia, Europe, and East Africa) were chosen to calculate the cumulative distribution of HLA class I and II alleles. Furthermore, the coverage of all epitopes from the vaccine candidate recognized by HLA alleles was computed on the basis of the coverage of the individual epitopes. Physiochemical analysis of the multi-epitope vaccine candidate was performed with the Expasy ProtParam server (https://web.expasy.org/protparam/) to evaluate molecular weight, instability index, estimated half-life, aliphatic index, and grand average of hydropathicity (GRAVY). An instability index < 40 indicates the stability of the vaccine candidates at room temperature [23]. The aliphatic index is regarded as a positive factor for increasing protein thermostability [24], and GRAVY is calculated as the sum of hydropathy values [25].

### Tertiary structure prediction, refinement, and assessment of the designed vaccine

After vaccine mapping, the sequence of the vaccine candidate was submitted to the I-TASSER server (https://zhanggroup.org//I-TASSER/) for tertiary structure prediction. I-TASSER, a platform for protein structure and function prediction, is ranked as the best method in critical assessment of protein structure prediction (CASP) experiments [26]. C-score is a crucial parameter that describes the confidence of predicted three-dimensional (3D) models, and a selected model should have a high C-score value [26]. The refinement of 3D model from the I-TASSER server was carried out using the GalaxyRefine server (http://galaxy.seoklab.org/cgi-bin/submit.cgi?type=REFINE). The GalaxyRefine server, which has shown the best performance in CASP10, rebuilds side chains, repacks side chains, and conducts overall structure relaxation via molecular dynamics simulation [27]. The quality of the refined and initial models was assessed using ProSA *z*-scores, Ramachandran plots, and 3D-1D scores. The ProSA-web (https://prosa.services.came.sbg.ac.at/prosa.php), SWISS-MODEL (https://swissmodel.expasy.org/interactive), and VERIFY 3D (https://saves.mbi.ucla.edu/) were used to analyze ProSA *z*-scores, Ramachandran plots, and 3D-1D scores, respectively.

### Molecular docking of TLRs and multi-epitope vaccine candidate

Molecular docking was conducted using the ClusPro server (https://cluspro.bu.edu/login.php?redir/queue.php). The ClusPro server, which is suitable for stiff docking, performs on the basis of rigid body docking, lowest energy structure clustering, and refinement [28]. TLR2 (PDB ID: 2Z7X) and TLR4 (PDB ID: 3FXI) acted as receptors, and multi-epitope vaccine candidate worked as ligands. A balanced coefficient model was selected to calculate the interaction energy. In addition, the docked complex structures in PDB format were submitted to the PDBsum server (https://www.ebi.ac.uk/thorntonsrv/databases/pdbsum/Generate.html) to analyze the interactions (hydrogen bonds and salt bridges) of the docked complexes. The interactions between the multi-epitope vaccine candidate and the TLRs were visualized using PyMOL software.

### Molecular dynamics simulation

Molecular dynamics (MD) simulations were used to assess the stability of the docked complexes. The ff19SB force field was used for proteins and peptides. Each complex in the presence of 0.15 M NaCl was immersed in a TIP3P water box that extends 10 Å from the solute surface. The LINCS algorithm was employed to constrain contacts involving hydrogen atoms. Electrostatic interactions were computed by Particle-mesh Ewald with a nonbonded cutoff of 1 Å. All MD simulations were performed in AMBER 20 software. Initially, to relieve unfavorable interactions and steric clashes, energy minimization was performed by the steepest descent method for the first 2500 steps and conjugated gradient method for the subsequent 2500 steps. Afterward, each system was warmed under the NVT ensemble over 200 ps by restraining the complex with a 1.0 kcal/(mol·Å2) force constant. Then, system equilibration with 310 K temperature and 1 bar pressure was conducted under NPT ensemble. Finally, a 100 ns production MD simulation was carried out, and trajectories were saved every 10 ps. The stability of the docked complexes was assessed by calculating the root mean square deviation (RMSD), root mean square fluctuation (RMSF), radius of gyration (Rg), and hydrogen bond (H-bond) interactions using CPPTRAJ in AmberTools20.

### Immune simulation

Immune simulations of the multi-epitope vaccine candidate and Leish-111f were carried out with the C-ImmSim server, which has been employed to assess antigen immunogenicity in previous publications [29–31]. This server (https://kraken.iac.rm.cnr.it/C-IMMSIM/index.php) simulates immune responses and immune interactions in bone marrow, thymus, and tertiary lymphoid organs [32]. The vaccine candidate and Leish-111f amino acid sequences were inputted into the C-ImmSim server. The simulation parameters were set as follows: simulation volume: 10; simulation steps: 540; HLA (A*03:01, A*11:01, B*15:01, B*35:01, DRB1*07:01, and DRB1*03:01); three injections at an interval of 14 days; and time steps including 1, 42, and 84, without LPS, num Ag to inject: 1000.

## Results

### HTL and CTL epitope prediction

We chose *Leishmania* HbR, KMP-11, Gp63, TSA, and SMT to design a multi-epitope vaccine. The IEDB server was employed for HTL and CTL epitope predictions on the basis of a percentile rank of < 10. Protein sequences were submitted and automatically split into 15-mer and 9/10-mer sequences by the IEDB server with user selected length through the algorithms of the server. The 15-mer sequences (HbR, 457 sequences; KMP-11, 78 sequences; Gp63, 627 sequences; TSA, 185 sequences; and SMT: 339 sequences) (Additional File 1, Supplementary Table S1) were used for HTL epitope prediction. HTL epitopes of HbR (305 epitopes), KMP-11 (57 epitopes), Gp63 (368 epitopes), TSA (120 epitopes), and SMT (214 epitopes) were identified (Additional File 1, Supplementary Table S2 and S3). The 9- and 10-mer sequences of HbR (925 sequences), KMP-11 (167 sequences), Gp63 (1265 sequences), TSA (381 sequences), and SMT (689 sequences) (Additional File 1, Supplementary Table S4) were used for CTL epitope prediction. HbR (628 epitopes), KMP-11 (127 epitopes), Gp63 (843 epitopes), TSA (263 epitopes), and SMT (460 epitopes) were identified (Additional File 1, Supplementary Table S5 and S6).

### Evaluation of the IFN-γ/IL-10 ratios and validation of the HTL and CTL epitopes

**Fig. 2:**
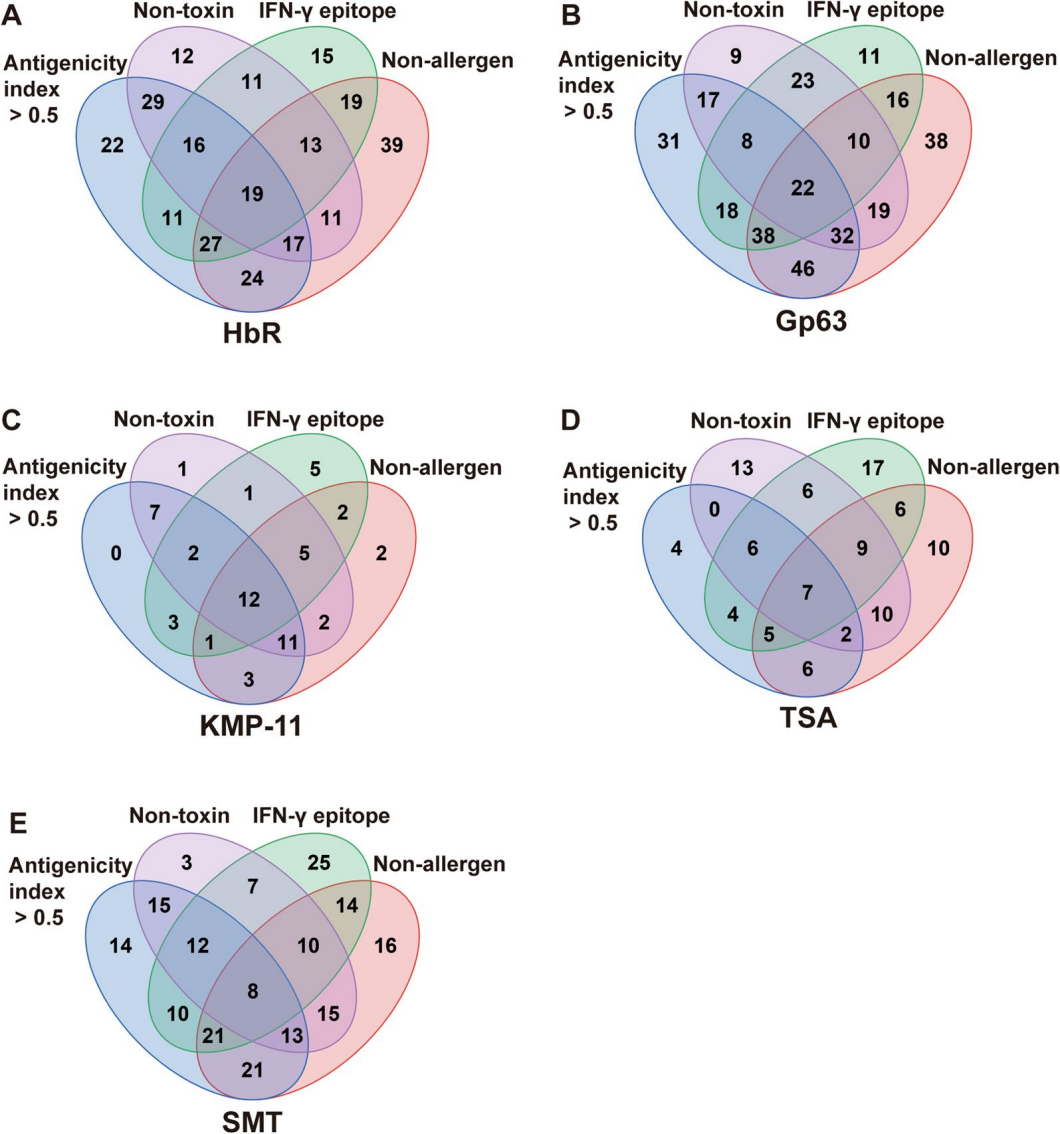
Results of antigenicity, toxicity, allergenicity, and IFN-γ-inducing epitope predictions for leishmanial HTL epitopes. **A** HbR; **B** Gp63; **C** KMP-11; **D** TSA; **E** SMT

To screen the HTL epitopes that may skew Th1 immune responses, the evaluation of IFN-γ/IL-10 ratio was performed. *Leishmania* HTL epitopes with antigenicity indices > 0.5, nontoxic and nonallergenic properties, and IFN-γ-inducing epitope potential were selected for further analysis (Fig. 2 and Additional File 1, Supplementary Table S7 and S8). These HTL epitopes included HbR (19 epitopes), KMP-11 (12 epitopes), Gp63 (22 epitopes), TSA (7 epitopes), and SMT (8 epitopes).

**Fig. 3:**
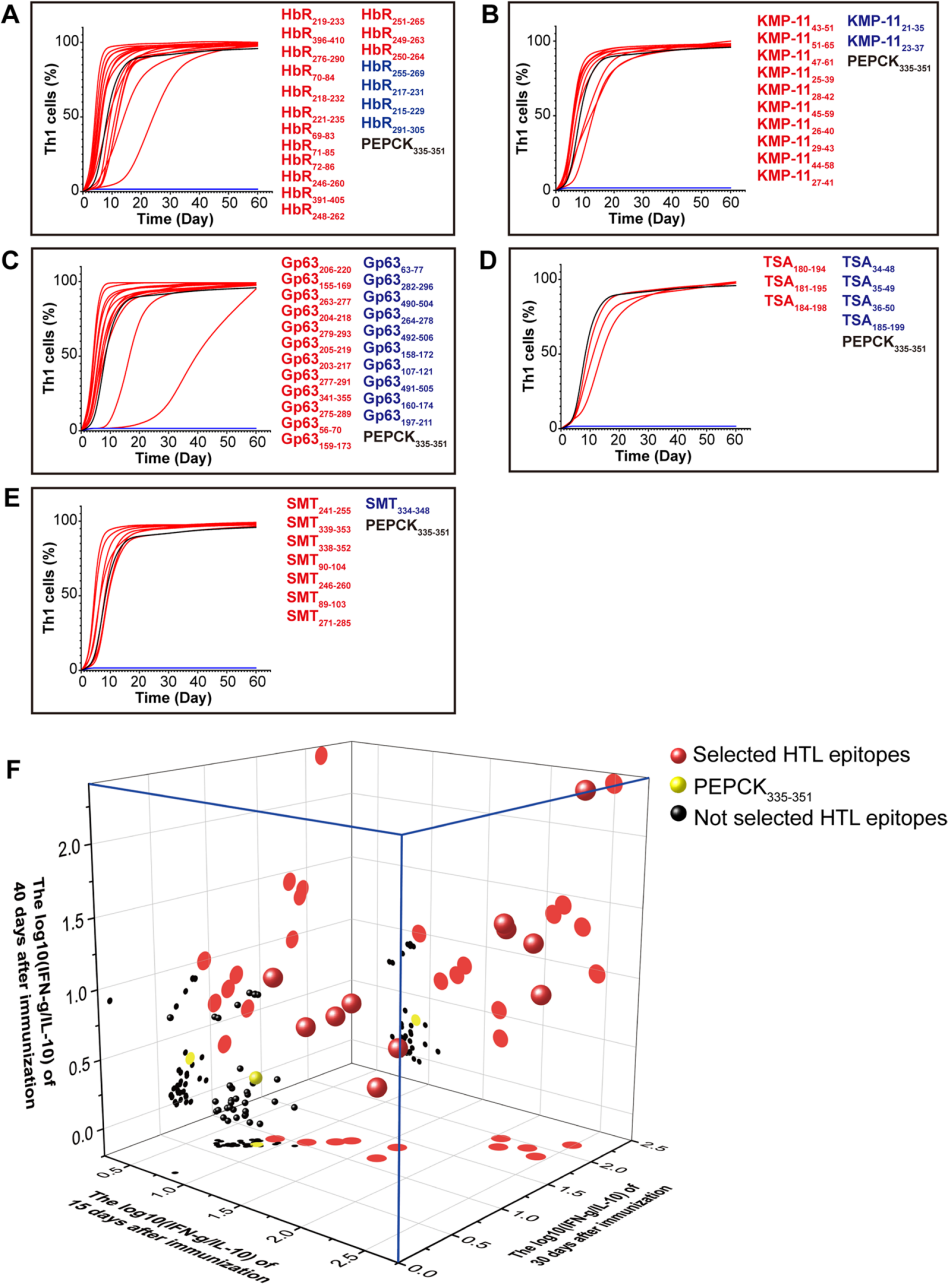
Th1 cell and IFN-γ/IL-10 ratio analysis. **A–E** HbR, KMP-11, Gp63, TSA, and SMT HTL epitopes with antigenicity indices > 0.5, nontoxic and nonallergenic properties, and IFN-γ-inducing epitope potential were used to predict Th1 cell generation using C-IMMSIM. The HTL epitopes that induced Th1 cells were marked with red lines, and those that failed to elicit Th1 cells were denoted with blue lines; **F** the HTL epitopes inducing Th1 cells and PEPCK_335–351_ (yellow spheres) were then analyzed for their IFN-γ/IL-10 ratios at 3 time points (on the 15th, 30th, and 40th day after immunization) with C-IMMSIM, and the results were presented as a 3D scatter plot. Epitopes showing higher IFN-γ/IL-10 ratios than those of PEPCK_335–351_ at 3 time points simultaneously were selected (red spheres). The remaining not selected epitopes were marked with black spheres. The colored plots on the planes formed by the axes are the projections of the spheres

We subjected the selected HTL epitopes and *Leishmania* PEPCK_335–351_ (as controls) to C-IMMSIM for the analysis of IFN-γ and IL-10 levels (Additional File 2, Supplementary Fig. S1). The C-IMMSIM results revealed that some epitopes failed to induce Th1 cells (Fig. 3A–E) and were excluded. The log10(IFN-γ/IL-10) values of the remaining epitopes were analyzed. PEPCK_335–351_ was used as a control in the analysis of IFN-γ/IL-10 ratio, because it activates strong Th1 immune responses and provides significant protection against leishmaniasis. [20]. Compared with the PEPCK_335–351_ ratio, 11 HTL epitopes presented higher ratios at 3 time points (Fig. 3F and Table 1) and were selected. HbR, KMP-11, Gp63, TSA, and SMT had CTL epitopes that had antigenicity indices > 0.5 and were nontoxic and nonallergenic (Additional File 1, Supplementary Table S9 and S10). Five CTL epitopes with low percentile ranks and high antigenicity indices were selected for the construction of the multi-epitope vaccine (Table 1 and Additional File 2, Supplementary Fig. S2). The selected HTL and CTL epitopes were blasted with human and mouse proteins, and no homologous sequence was found. In brief, these selected epitopes will serve as the epitopes of the multi-epitope vaccine candidate.

**Table 1:** Selected epitopes for the multi-epitope vaccine construction and PEPCK sequence

Epitope type	Epitope	Sequence	Percentile rank	Antigenicity/allergenicity/toxicity	The log10 (IFN-γ/IL-10) 15th, 30th, and 40th day after immunization
HTL	HbR_219–233_	LVARYFVDTDVQVGV	0.13	0.9052/NA/NA	2.291/1.928/1.288
HTL	HbR_221–235_	ARYFVDTDVQVGVII	0.45	1.3064/NA/NA	2.133/1.817/1.422
HTL	HbR_276–290_	KYKYALPITVYDDEM	1.9	1.2343/NA/NA	1.100/1.045/0.656
HTL	HbR_218–232_	TLVARYFVDTDVQVG	0.16	0.8039/NA/NA	2.054/1.954/1.341
HTL	HbR_250–264_	VTKDPAVSARGNAVT	9.9	0.9514/NA/NA	2.421/1.841/0.970
HTL	Kmp-11_51–65_	FERMIKEHTEKFNKK	2.3	0.649/NA/NA	1.623/1.370/0.535
HTL	Gp63_56–70_	RVRQSVARHHTAPGA	2.3	0.6758/NA/NA	2.486/2.536/2.329
HTL	Gp63_275–289_	TEILVVTQMMNIRGK	2.3	0.987/NA/NA	0.895/0.954/0.994
HTL	TSA_181–195_	APTMKPEPKASVEGY	2.3	0.9824/NA/NA	1.642/1.134/0.318
HTL	SMT_90–104_	HEYFLAARGGFMEGD	0.37	0.5668/NA/NA	1.311/1.263/0.820
HTL	SMT_89–103_	RHEYFLAARGGFMEG	0.9	0.5435/NA/NA	1.244/1.179/0.732
CTL	HbR_301–310_	RQEKLVSGMY	0.11	1.8986/NA/NA	
CTL	Kmp-11_53–61_	RMIKEHTEK	0.05	0.8008/NA/NA	
CTL	Gp63_228–236_	QVFSDGHPA	0.32	1.6761/NA/NA	
CTL	SMT_337–345_	IFTPSFYIR	0.09	2.1097/NA/NA	
CTL	TSA_187–196_	EPKASVEGYF	0.16	1.6472/NA/NA	
PEPCK	PEPCK_335–351_	NDAFGVMPPVARLTPEQ	0.12	0.285/NA/NA	0.873/0.799/0.298

### Construction of the multi-epitope vaccine candidate

**Fig. 4:**
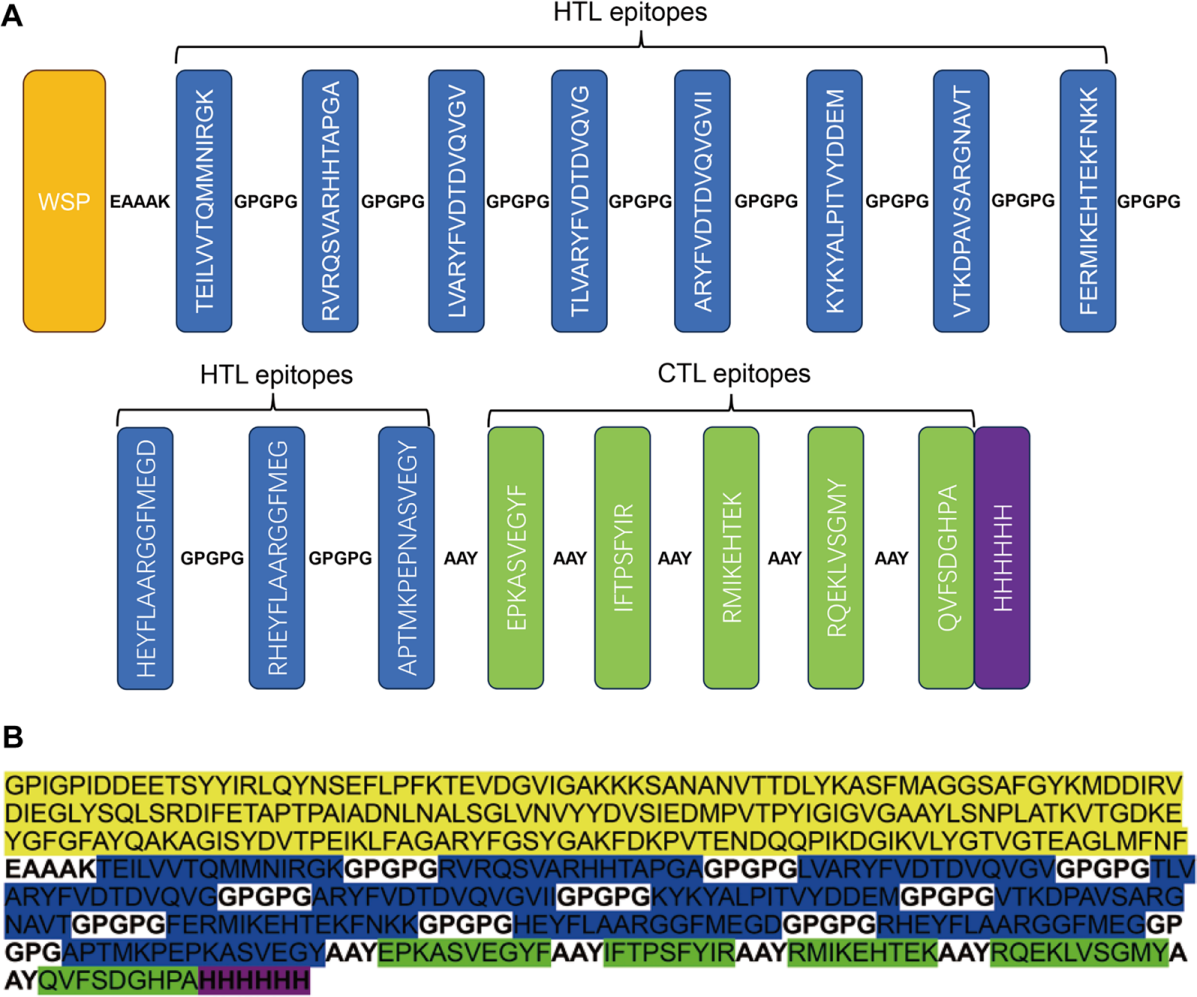
Construction and amino acid sequence of the multi-epitope vaccine candidate. **A** Schematic representation of the multi-epitope vaccine candidate construction; **B** amino acid sequence of the multi-epitope vaccine candidate: WSP (yellow), selected HTL (blue) and CTL (green) epitopes, and 6 × His tag (purple)

As presented in Table 1, a total of 11 HTL epitopes and 5 CTL epitopes were used to develop a multi-epitope vaccine candidate. The HTL epitopes were connected with GPGPG linkers, while AAY linkers were employed to merge the CTL epitopes. TLR2 and TLR4 activation of *Wolbachia* surface protein (WSP) has been reported, which is important for *Leishmania* vaccines. [13, 17, 18]. WSP was attached to the N-terminal of the vaccine candidate through the connection of an EAAAK linker. Finally, a 6 × His tag sequence was added to the C-terminal of the vaccine candidate for identification and purification of protein expression (Fig. 4). The vaccine candidate was not similar to human or mouse proteins. It had an antigenicity index of 0.666 and was nonallergenic and nontoxic, suggesting its potential as a safe and effective antigen.

### Evaluation of the population coverage of vaccine candidate

**Fig. 5:**
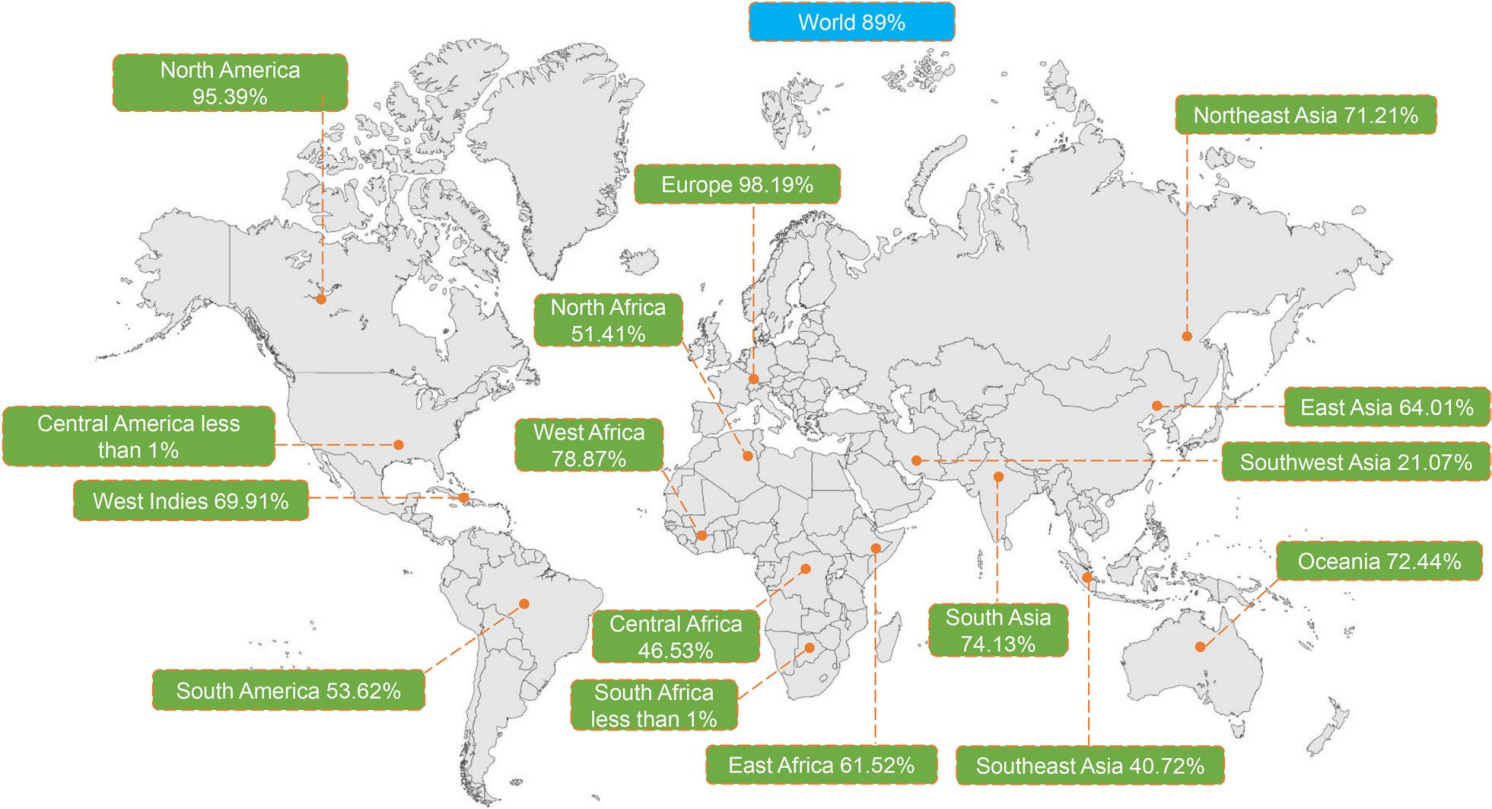
Coverage of all epitopes simultaneously recognized by HLA alleles

MHC polymorphisms and dramatically different frequencies across different ethnicities lead to consider the population coverage of vaccine. The distribution of HLA alleles was evaluated using MHC I and II combined mode to analyze the geographical distribution and population coverage of the vaccine candidate. According to the statistical results, the selected HTL and CTL epitopes were recognized by multiple alleles in the world. Most regions exhibited high cumulative distribution rates (Additional File 1, Supplementary Table S11). The cumulative coverage of each epitope across most geographical regions exceeded 87%, with the exception of South Africa (Additional File 1, Supplementary Table S11). Coverage of all epitopes being simultaneously recognized by HLA was also calculated on the basis of the coverage of individual epitopes. The results revealed more than 50% coverage rates in most regions (Fig. 5 and Additional File 1, Supplementary Table S11). In summary, the population coverage analysis illustrated that the designed vaccine candidate may be recognized by the majority of people and may confer protection across diverse geographical regions.

### Physiochemical characteristics of multi-epitope vaccine candidate

To evaluate the stability of the multi-epitope vaccine candidate, physiochemical properties, including molecular weight, instability index, estimated half-life, aliphatic index, and grand average of hydropathicity (GRAVY), were determined with the help of Expasy ProtParam server. The multi-epitope vaccine candidate (formula: C2420H3656N636O716S13) comprised 500 amino acids and had a molecular weight of 53532.26, a theoretical isolectric point (pI) of 5.48, 55 negatively charged residues, and 46 positively charged residues. The vaccine candidate exhibited an estimated half-life of 30 h (mammalian reticulocytes, in vitro), > 20 h (yeast, in vivo), and > 10 h (*E. coli*, in vivo). The aliphatic index of the vaccine candidate was 67.14, indicating the thermostability of the vaccine candidate at body temperature. The instability index of the vaccine candidate was calculated as 20.23, which is smaller than 40, indicating the stability of the vaccine candidate at room temperature [23]. The GRAVY of the vaccine candidate was −0.3, suggesting its hydrophilic nature [24].

### Tertiary structural modeling of the designed vaccine candidate

**Fig. 6:**
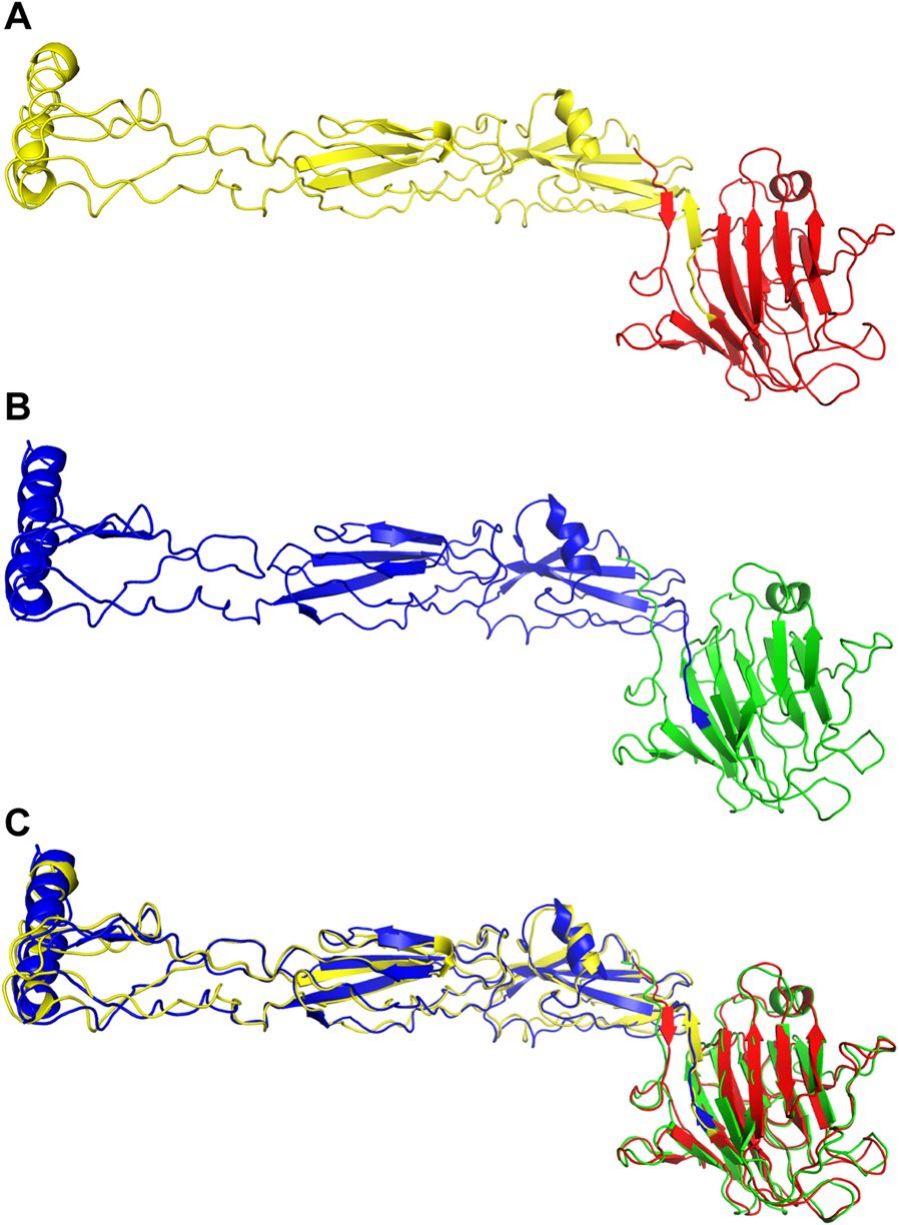
Vaccine candidate model and superimposition display. **A** Initial model of the vaccine candidate (WSP colored with red, epitopes colored with yellow); **B** refined model of the vaccine candidate (WSP colored with green, epitopes colored with blue); **C** superimposition of the initial model (yellow epitopes and red WSP) and the refined model (blue epitopes and green WSP)

The tertiary structure of the designed vaccine was generated to analyze the interactions between the vaccine candidate and TLRs. The I-TASSER server generated five top-ranked tertiary structure models after submitting the vaccine candidate sequence. The first model with the largest C-score value was chosen as initial tertiary structure model (Fig. 6A), and it was further refined through GalaxyRefine server to enhance 3D structure model quality. After refinement, five refined models were provided with key parameters (GDT-HA and MolProbity, which contains Clash score, Poor rotamer, and Ramafavored region). A higher GDT-HA score indicates increased backbone structure accuracy, and a lower MolProbity score suggests superior mode quality [33]. The 3D-1D scores from Verify3D were computed to measure the compatibility of the refined model structure with its sequence. A higher 3D-1D score of residues indicates favorable compatibility with 3D structure [34]. Then, refined model 5 (Fig. 6B) was selected because 80.8% of the residues had a 3D-1D average score > = 0.1, a GDT-HA score of 0.9045, and a MolProbity score of 2.234 (Table 2). The alignment between the initial and refined models was performed (Fig. 6C), and the root mean square deviation (RMSD) was 1.544 Å, indicating the distinction between the initial and refined models.

**Table 2:** Refined models from the GalaxyRefine server

Model	3D-1D score %	GDT-HA	MolProbity	Clash score	Poor rotamers	Rama favored	RMSD
Initial model	75.2	1	3.435	12.2	18.9	66.9	0
Refined model 1	78.4	0.9021	2.258	16.3	16.3	90.2	0.205
Refined model 2	80.0	0.8998	2.271	17.1	17.1	90.4	0.231
Refined model 3	79.4	0.9045	2.306	17.0	17.0	89.0	0.180
Refined model 4	78.2	0.9	2.245	15.8	15.8	90.2	0.191
Refined model 5	80.8	0.9045	2.234	15.8	15.4	90.2	0.222

### Assessment and validation of the 3D structure of the designed vaccine candidate

**Fig. 7:**
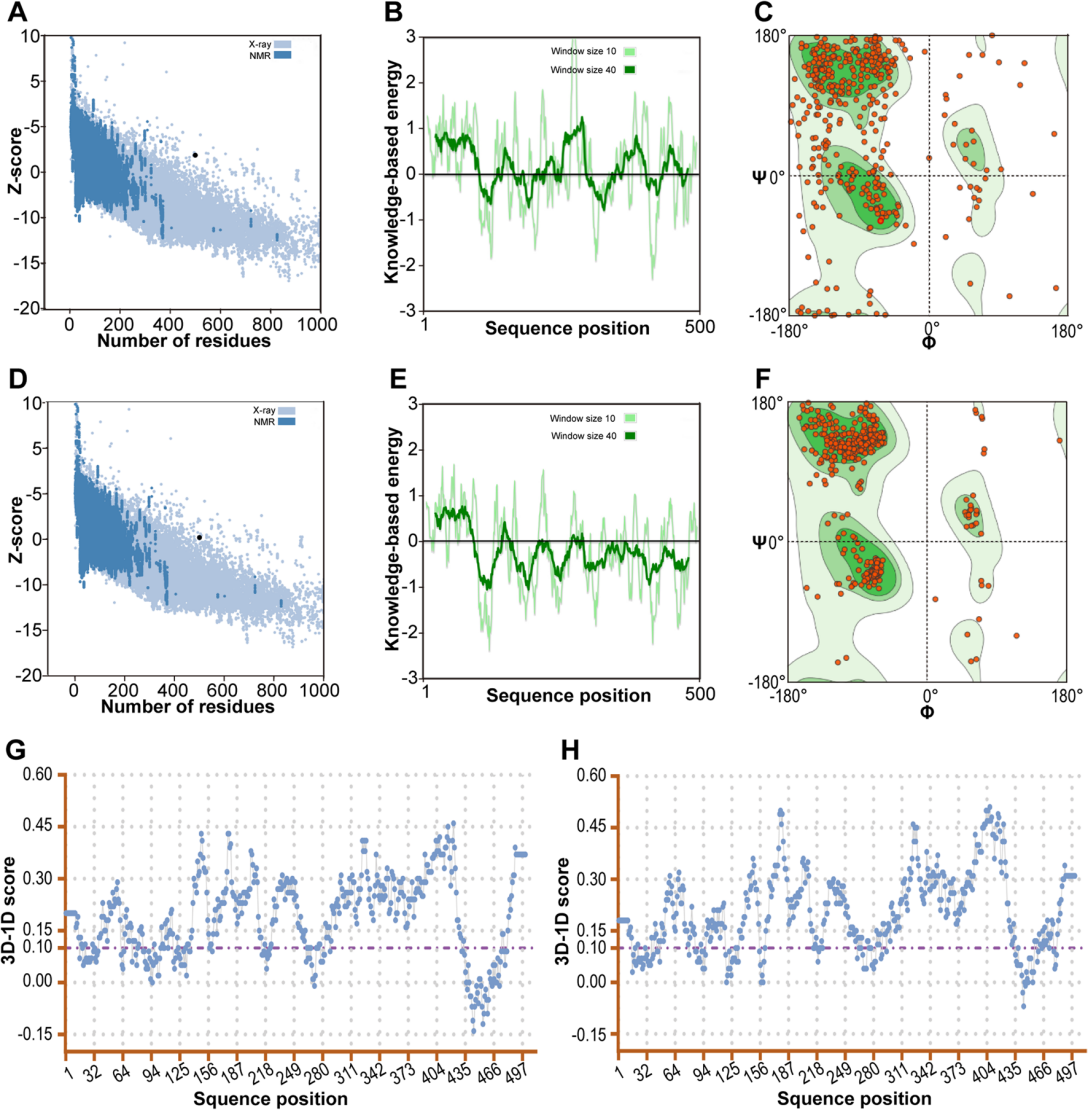
ProSA *z*-score, local energy distribution, Ramachandran plot, and 3D-1D scores of the initial and refined models. **A** ProSA *z*-score of the initial model; **B** local energy distribution of the initial model; **C** Ramachandran plot of the initial model; **D** the ProSA *z*-score of the refined model; **E** local energy distribution of the refined model; **F** Ramachandran plot of the refined model; **G** the initial model had 75.2% residues with 3D-1D scores ≥ 0.1; **H** the refined model had 80.8% residues with 3D-1D scores ≥ 0.1

The ProSA *z*-score, Ramachandran plot, and 3D-1D score of the initial and refined models were assessed to confirm the effect of the refinement. The *z*-scores of the initial and refined models were −3.09 and −4.83, respectively. Energy plots revealed that the refined model was within the range of native conformations and was closer to the majority of *z*-scores from X-ray structures (Fig. 7A and D). Compared with the refined model, the initial model exhibited higher positive local energy values (Fig. 7B and E), suggesting a reduction in problematic structure after refinement. The results of Ramachandran plot analyzed by SWISS-MODEL revealed the initial model with 65.15% favored regions and 17.43% outlier regions and the refined model with 91.16% favored regions and 2.01% outlier regions. (Fig. 7C and F). With respect to the percentage of residues with an average 3D-1D score ≥ 0.1, the initial model exhibited 75.2% residues, whereas the refined model showed 80.8% residues (Fig. 7G and H, and Table 2). These improved values indicate the enhanced quality of the refined 3D structure.

### Protein‒protein docking between TLRs and the designed vaccine

**Fig. 8:**
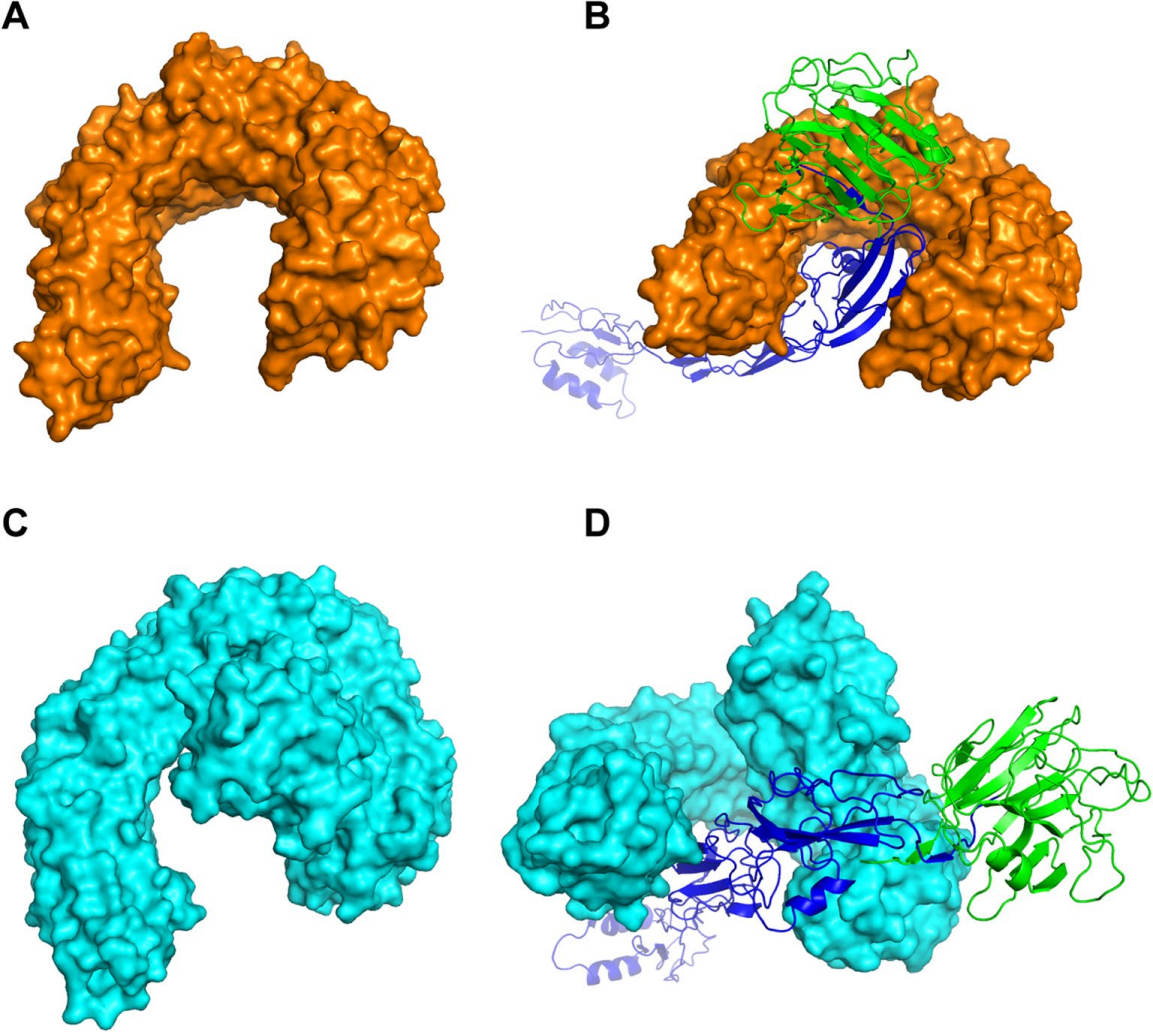
Three-dimensional structures of the TLRs and the TLR-vaccine candidate docked complexes. TLR2 and TLR4/MD2 were docked with the designed vaccine candidate. **A** TLR2 structure; **B** TLR2 (orange surface structure)-vaccine candidate (cartoon structure, blue epitopes and green WSP) docked complex structure; **C** TLR4/MD2 structure; **D** TLR4/MD2 (cyan surface structure)-vaccine candidate (cartoon structure, blue epitopes and green WSP) docked complex structure

Molecular docking between TLR2, TLR4, and the designed vaccine candidate was carried out using the ClusPro server to investigate the interactions between the TLRs and the multi-epitope vaccine candidate. In accordance with the cluster size of the docked complexes and the conformation of reported analogous docked structures [29, 31], the TLR2-vaccine candidate docked complex (74 clusters, −1345.1 lowest energy) and the TLR4/MD2-vaccine candidate docked complex (34 clusters, −1068 lowest energy) (Fig. 8) were taken for further analysis.

**Fig. 9:**
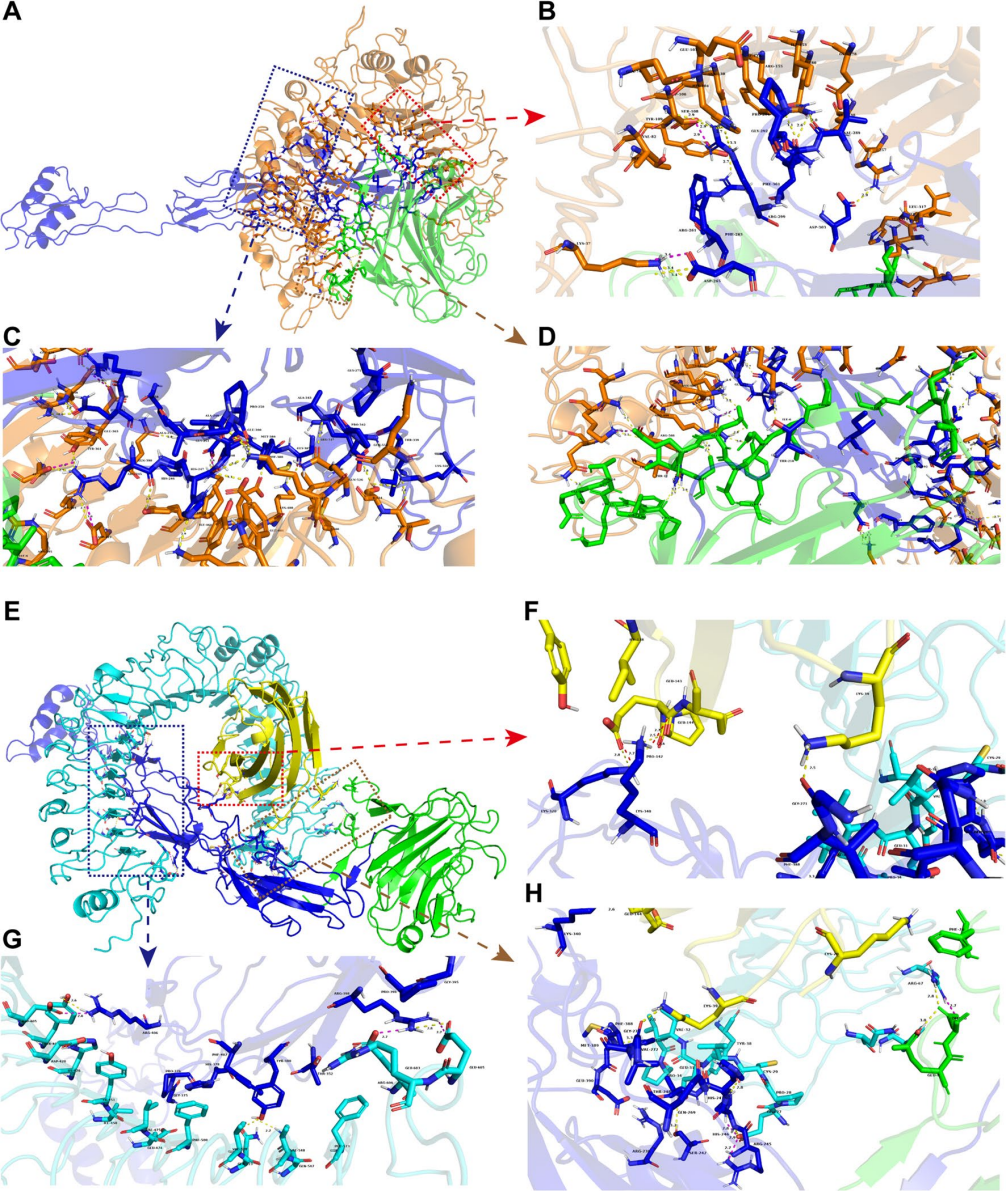
Representation of interactions between the TLRs and the vaccine candidate. Hydrogen bonds and salt bridges were marked with yellow and red dotted lines, respectively. **A** TLR2-vaccine candidate docked complex. TLR2, epitopes, and WSP were colored orange, blue, and green, respectively; **B–D** interactions between TLR2 and the vaccine candidate; **E** TLR4/MD2-vaccine candidate docked complex. TLR4, MD2, epitopes, and WSP were colored cyan, yellow, blue, and green, respectively; **F–H** interactions between TLR4/MD2 and the vaccine candidate

The docked complexes between TLR2, TLR4/MD2, and the vaccine candidate were submitted to PDBsum for interaction analysis. The TLR2-vaccine candidate docked complex revealed 49 hydrogen bonds and 10 salt bridges (Fig. 9A–D, Additional File 1, and Supplementary Table S12). In the TLR2-docked complex, 16 hydrogen bonds (32.6% hydrogen bonds) and two salt bridges (20% salt bridges) formed in WSP. With respect to the TLR4/MD2-vaccine candidate complex, 14 hydrogen bonds and six salt bridges were found between TLR4 and the vaccine candidate, whereas the interactions between MD2 and the vaccine candidate formed 4 hydrogen bonds and two salt bridges (Fig. 9E–H, Additional File 1, and Supplementary Table S12). In the TLR4-docked complex, three hydrogen bonds (16.7% hydrogen bonds) and one salt bridge (12.5% salt bridges) occurred in WSP. In brief, the interactions between the TLRs and the vaccine candidate occurred at the N-terminal region of the vaccine candidate, suggesting the interaction between WSP and the TLRs (Fig. 9D–H, Additional File 1, and Supplementary Table S12). These results imply that the multi-epitope vaccine candidate may be recognized by TLR2 and TLR4 to trigger leishmanicidal immune responses.

### Molecular dynamics simulation

**Fig. 10:**
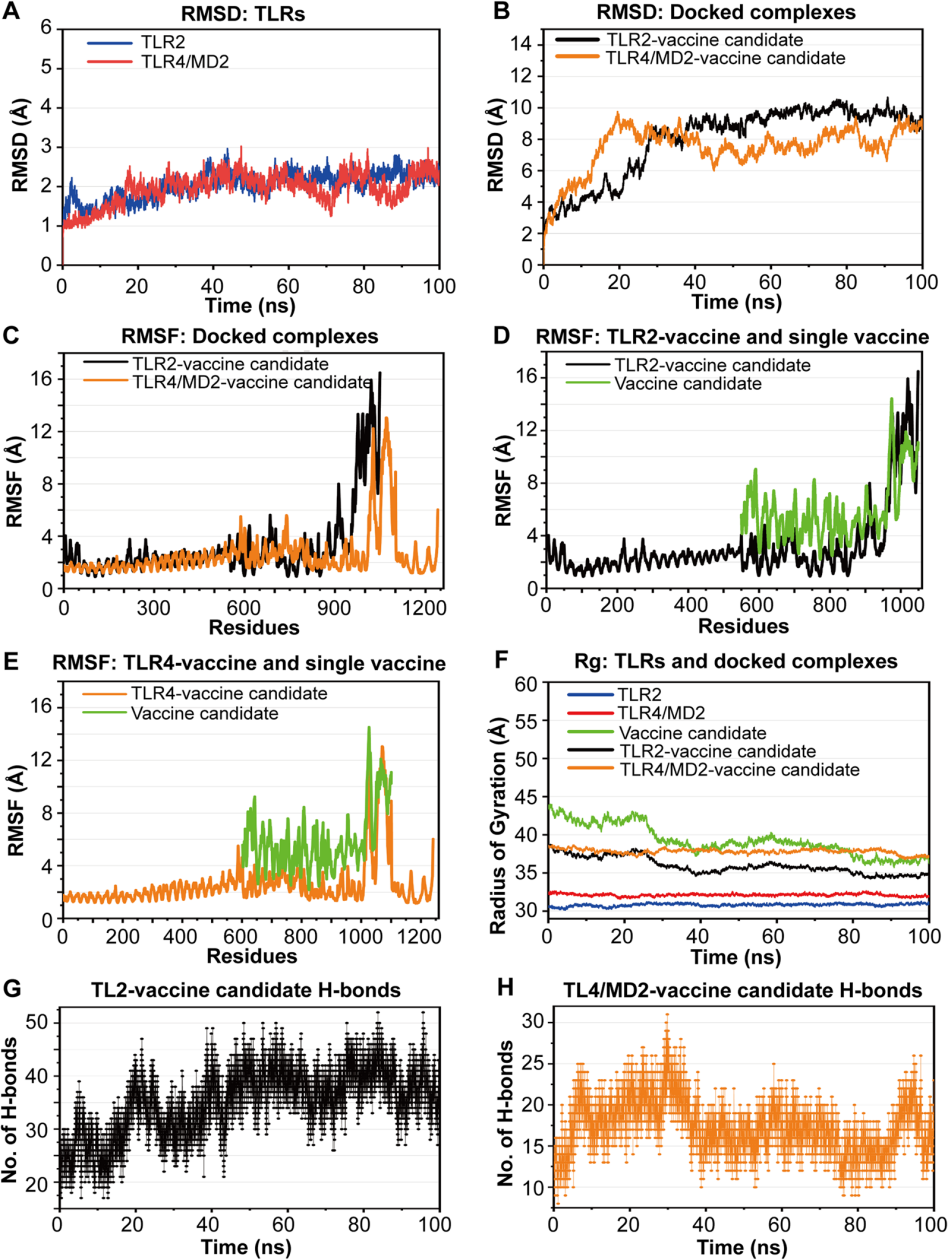
Molecular dynamics simulation results. **A** TLR2 and TLR4/MD2 RMSD; **B** RMSD of the TLR-vaccine candidate docked complexes; **C** RMSF of the TLR-vaccine candidate docked complexes; **D–E** RMSF of the single vaccine candidate and the docked complexes; **F** Rg values of TLR2, TLR4/MD2, the vaccine candidate, and the docked complexes; **G, H** analysis of intermolecular H-bonds in the docked complexes

To assess the stability, flexibility, compactness, and intermolecular contacts of the docked complexes, a 100 ns MD simulation was performed for each complex. According to the RMSD results, TLR2 and TLR4/MD2 slightly fluctuated at approximately 2 Å owing to their naturally stable structures (Fig. 10A). The TLR2-vaccine candidate and TLR4/MD2-vaccine candidate docked complexes began to stabilize at 20 ns and 29 ns, respectively, and their RMSD remained stable throughout the rest of MD simulation (Fig. 10B). These docked complexes had high RMSD values, which may be due to the flexible linkers and dynamic structural behaviors of the docked complexes [33]. In summary, the RMSD of the docked complexes ultimately exhibited a steady phase, indicating conformational stability during MD simulation. The 1–500 amino acids from the docked complexes had a stable RMSF of approximately 2 Å, and 600–1240 AAs had obvious RMSF fluctuations near 0.9–16.1 Å (Fig. 10C), which suggests that the docked complexes have both stability and flexibility [35]. The RMSF values of the single vaccine candidate were compared with those of the vaccine candidates within the TLR docked complexes (the vaccine candidate within the TLR2 and TLR4 docked complexes ranging from 550 to 1049 AA and 602 to 1101 AA, respectively) (Fig. 10D–E). The RMSF values of almost amino acids from the single vaccine candidate were greater than those of the vaccine candidate within the docked complexes, further supporting the stability of the docked complexes, which is consistent with the increased interactions between the vaccine candidate and the TLRs. Compared with the vaccine candidate, the docked complexes presented smaller radius of gyration (Rg) values for the majority of the simulation time (Fig. 10F), which may indicate that the docked complexes remain folded and show their stability and extensibility. The docked complexes showed obvious fluctuations in H-bonds (Fig. 10G–H), which may be due to the diversity of H-bond types that involve variations in the distance, angles, and energies between the donor and acceptor [33]. The average numbers of H-bonds in the docked complexes during MD were 17 for the TLR4/MD2-vaccine candidate and 34 for the TLR2-vaccine candidate. These results were consistent with the results of the PDBsum interaction analysis, suggesting the stability of the docked complexes.

### Immune simulation of the multi-epitope vaccine candidate

**Fig. 11:**
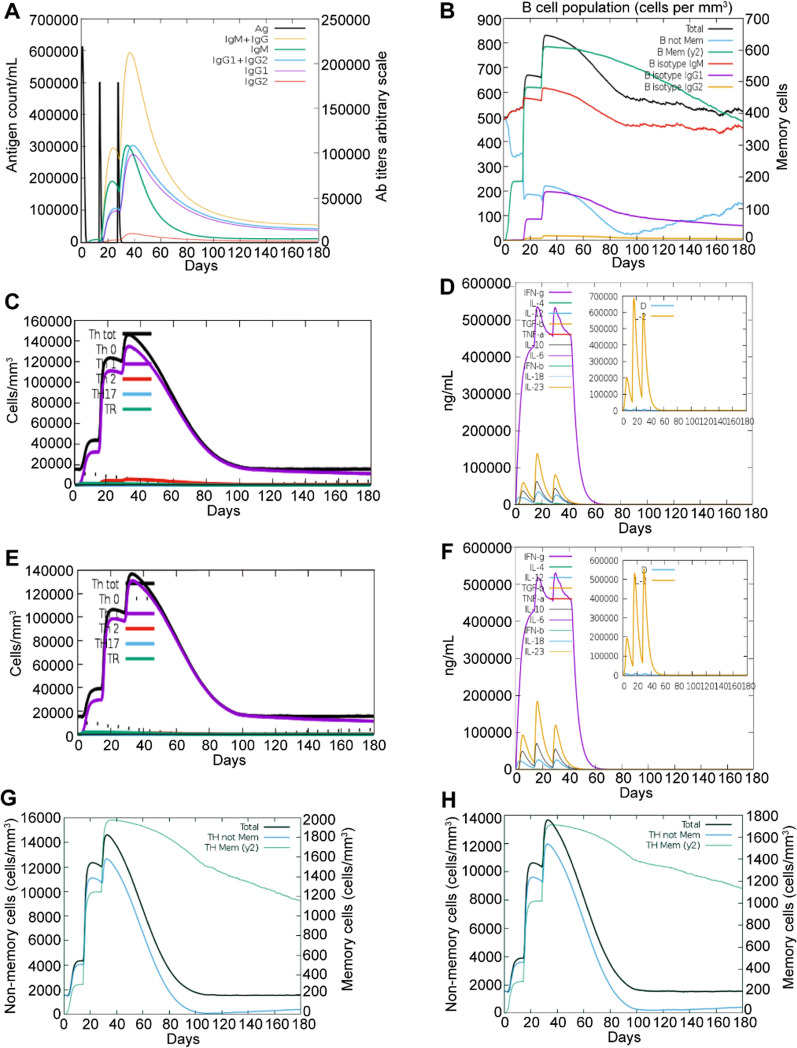
Immune simulation results of the multi-epitope vaccine candidate and Leish-111f from the C-ImmSim server. **A**–**D** Immune simulation results of the multi-epitope vaccine candidate; **A** antibody production after three Ag injections; **B** B cell formation; **C** changes in T isotype cells; **D** production of cytokines; **E, F** production of T isotype cells (**E**) and cytokines (**F**) in response to Leish-111f. IL-2 and the danger signal denoted as D are presented by yellow and blue thick lines in the insert plot of the cytokine results, respectively; **G, H** the memory Th cell production of the vaccine candidate (**G**) and Leish-111f (**H**)

The multi-epitope vaccine candidate and Leish-111f (as a control) were subjected to the C-ImmSim server to evaluate the immunogenicity of the vaccine candidate. The results revealed that immunoglobulin (Ig)M and IgG appeared and peaked following immunization (Fig. 11A). IgG expression was associated with the formation of IgG B isotype cells after the second antigen (Ag) injection (Fig. 11B), indicating isotype switching with the help of corresponding CD4 T cells specific for *Leishmania* epitopes [36, 37]. Furthermore, total helper T cells appeared, and Th1 cells showed a significant increase (Fig. 11C). The vaccine candidate induced relatively few Th2 cells, and its Th1 cell production slightly exceeded that of Leish-111f (Fig. 11C and E). During the immune responses, cytokines (IFN-γ, IL-2, IL-12, IL-10, etc.) were induced with increasing Ag injection (Fig. 11D). Compared with Leish-111f, our vaccine candidate showed a similar trend toward changes in Th1 cell, IFN-γ, IL-12, IL-2, and IL-10 (Fig. 11C–F). The maximum concentrations of IFN-γ, IL-12, and IL-2 induced by the vaccine candidate were slightly higher than those induced by Leish-111f, whereas the maximum concentration of IL-10 was slightly lower than that induced by Leish-111f. Memory T cell induction indicates the immunogenicity and efficacy of vaccines [38]. The production of memory Th cells induced by the vaccine candidate was greater than that induced by Leish-111f (Fig. 11G–H). In summary, on the basis of immune simulation in silico, our multi-epitope vaccine candidate may trigger favorable leishmanicidal immune responses.

## Discussion

Approaches to screening desirable epitopes are important for the development of multi-epitope vaccines. Classical approaches determine peptides as T epitopes through stimulation with synthetic peptides of T cell clones and classical competitive inhibition assay [7, 39, 40]. However, it is obvious that time, energy, and cost are required by classical and developed classical approaches because of complicated protocols and many peptides that need to be studied. These drawbacks could be saved by immunoinformatics, which offers available tools for epitope identification. Immunoinformatics is concerned with epitope prediction in silico and screens epitopes from many peptides within a short time. Currently, immunoinformatics tools have been employed universally to predict epitopes from known antigenic proteins to develop multi-epitope vaccines against *Leishmania*, influenza virus, severe acute respiratory syndrome coronavirus 2 (SARS-CoV-2), etc. [12, 30, 33, 41]. Immunoinformatics indeed predicts epitopes, yet it overlooks immune response biases triggered by the selected epitopes. Therefore, we integrated an additional strategy that further screens HTL epitopes with high IFN-γ/IL-10 ratios for the development of a leishmanicidal vaccine.

It is generally acknowledged that Th1 responses are associated with *Leishmania* parasite clearance, whereas Th2 responses favor disease progression. The development of Th1 response requires CD4 T cells to recognize specific epitopes presented by MHC II for activation. Although further elucidation is needed to determine whether CD8 T cells are protective in leishmaniasis, CD8 T cells appear to provide resistance to reinfection [42]. As a result, our multi-epitope vaccine candidate contained CTL and more HTL epitopes, and more consideration was given to HTL epitope screening. In addition, further analysis was based on antigenicity and safety. Those epitopes with antigenicity indices > 0.5 were selected because of the ability of antigen to bind with immune effector cells [39]. Allergic and toxic epitopes were simultaneously excluded to avoid allergic reactions and toxicity.

In our previous work, the IFN-γ/IL-10 ratio was used to screen differentially expressed proteins as antigen candidates [29]. The IFN-γ/IL-10 ratio served as a criterion for epitope selection in this study. Because of the critical role of Th1 responses in *Leishmania* clearance, 21 HTL epitopes from HbR, KMP-11, Gp63, TSA, and SMT, which failed to induce Th1 cells, were excluded. PEPCK_335–351_, which has been found to elicit polyfunctional CD4^+^ T cell responses and provide good protection against leishmaniasis, was used as a control for our analysis [20]. In addition, 11 remaining epitopes with IFN-γ/IL-10 ratios at 3 time points that were simultaneously higher than those of PEPCK_335–351_ were subsequently used to construct the multi-epitope vaccine candidate [20]. CTL epitopes with low percentile ranks and high antigenicity index values were chosen for the multi-epitope vaccine development.

Epitope vaccines are very poor immunogens and require assistance from adjuvants to promote immune responses [6]. In our study, WSP as an adjuvant was linked to the N-terminal of our vaccine candidate. The interface analysis of the TLR-docked complexes revealed interactions between WSP and the TLRs, suggesting that WSP may be recognized by TLR2 and TLR4. Moreover, interactions were also found between the *Leishmania* epitopes and the TLRs, which indicates the self-adjuvant potential of the multi-epitope segment. In our study, steady-phase RMSD, more stable RSMF, decreased Rg, and H-bond formation demonstrated the stable structure of the docked complexes, supporting the interaction between the TLRs and WSP and the *Leishmania* epitopes. WSP, as an adjuvant conjugated with the multi-epitope vaccine candidate, may be recognized by TLR2 and TLR4 to elicit a favorable immune response against *Leishmania*.

Leish-111f has been reported to have desirable protection against VL and has been tested in clinical development [4]. In this study, the immune responses of the multi-epitope vaccine candidate and Leish-111f were predicted in silico. The multi-epitope vaccine candidate induced the production of IgM, IgG1, and IgG2, as well as IgG B isotype cells, implying that antibody isotype switching occurred. Because antibody isotype switching requires the assistance of activated CD4 follicular helper T cells, the production of IgG indicates that the multi-epitope vaccine candidate may be recognized by epitope-specific CD4 T cells [37]. According to the immune simulation results, including Th1 cell induction, cytokine production (IFN-γ, IL-12, IL-2, and IL-10), and memory Th cell generation, these findings supported the factor that the vaccine candidate might induce strong desirable Th1 responses, indicating its potential immunogenicity.

Our study presents a novel strategy of multi-epitope vaccine development through multiple bioinformatics. However, bioinformatics-based vaccine design heavily relies on prediction methods, which leads to the limits of prediction accuracy. The actual efficacy of our vaccine candidate remains uncertain and requires verification by experiments in vivo. In addition, WSP as an adjuvant may be susceptible to degradation in the sites of injection, and its efficacy should be evaluated by comparing with other standard adjuvants. Consequently, other immunostimulatory and particulate delivery adjuvants should be administrated with the vaccine candidate to ensure the sure the best strategies through animal experiments.

## Conclusions

For the development of a vaccine against *Leishmania*, epitopes were screened in silico on the basis of the IFN-γ/IL-10 ratios, and *Wolbachia* surface protein was integrated as an adjuvant. Compared with Leish-111f, our multi-epitope vaccine candidate elicited appropriate immune responses in silico. Our vaccine candidate may interact with TLR2 and TLR4 and exhibit good immunogenicity, favoring *Leishmania* clearance. Our strategy for developing a multi-epitope vaccine offers a reference for the development of leishmaniasis and other infectious disease vaccines.

## Supplementary Information

Additional File 1: Supplementary Table S1. The IEDB input sequences of HbR, KMP-11, Gp63, TSA, and SMT for HTL epitope prediction; Supplementary Table S2. The HTL epitopes of HbR, KMP-11, Gp63, TSA, and SMT identified by IEDB; Supplementary Table S3. The original HTL prediction results of HbR, KMP-11, Gp63, TSA, and SMT identified by IEDB; Supplementary Table S4. The IEDB input sequences of HbR, KMP-11, Gp63, TSA, and SMT for CTL epitope prediction; Supplementary Table S5. The CTL epitopes of HbR, KMP-11, Gp63, TSA, and SMT identified by IEDB; Supplementary Table S6. The original CTL prediction results of HbR, KMP-11, Gp63, TSA, and SMT identified by IEDB. Supplementary Table S7. HTL epitopes with antigenicity indices > 0.5, nontoxic and non-allergenic properties, and IFN-γ-inducing epitope potential from HbR, KMP-11, Gp63, TSA, and SMT; Supplementary Table S8. Antigenicity, allergenicity, toxicity, and IFN-g-inducing epitope potential analysis results from HTL epitopes; Supplementary Table S9. Non-allergenic and non-toxic CTL epitopes with antigenicity index > 0.5 from HbR, KMP-11, Gp63, TSA, and SMT; Supplementary Table S10. Antigenicity, allergenicity, and toxicity analysis results from CTL epitopes; Supplementary Table S11. Coverage of epitopes from the vaccine candidate recognized by HLA alleles/The distribution of HLA I and HLA II alleles recognizing epitopes in the world; Supplementary Table S12. The interfaces between TLRs and vaccine candidate.

Additional File 2: Fig. 1: The cytokines of immune simulations from HTL epitopes with antigenicity index > 0.5 non-toxic and non-allergenic properties, and IFN-γ-inducing epitope potential; Fig. 2: The distribution of CTL epitopes based on percentile rank and antigenicity.

## Data Availability

No datasets were generated or analyzed during the current study.

## Abbreviations

VL: Visceral leishmaniasis
HTL: Helper T lymphocyte
CTL: Cytotoxic T lymphocyte
*L. donovani*: *Leishmania donovani*
*L. infantum*: *Leishmania infantum*
MHC: Major histocompatibility complex
HbR: Hemoglobin receptor
KMP-11: Kinetoplastid membrane protein-11
Gp63: Glycoprotein of 63KDa
TSA: Thiol-specific antioxidant antigen
SMT: Sterol 24-c-methyltransferase
WSP: *Wolbachia* Surface protein
TLR: Toll-like receptor
HLA: Human leukocyte antigen
IEDB: Immune Epitope Database server
CASP: Critical assessment of protein structure prediction
GRAVY: Grand average of hydropathicity
MD: Molecular dynamics
APCs: Antigen-presenting cells
RMSD: Root mean square deviation
iRMSD: Interface root mean square deviation
RMSF: Root mean square fluctuation
Rg: Radius of gyration
AA: Amino acid
