# Supplementary material for: Screening helper T lymphocyte epitopes based on IFN-γ/IL-10 ratio for developing a novel multi-epitope vaccine candidate using Wolbachia surface protein as an adjuvant against visceral leishmaniasis
Source: Parasit Vectors. 2025 Mar 25;18:116. doi: 10.1186/s13071-025-06756-5 (PMC11938772; doi:10.1186/s13071-025-06756-5)
Supplement: Supplementary file 2 — Additional File 2: Fig. 1: The cytokines of immune simulations from HTL epitopes with antigenicity index > 0.5 non-toxic and non-allergenic properties, and IFN-γ-inducing epitope potential; Fig. 2: The distribution of CTL epitopes based on percentile rank and antigenicity. [file 13071_2025_6756_MOESM2_ESM.docx]

**Additional file 2 contains:**

**Figure S1:** The cytokines of immune simulations from HTL epitopes with antigenicity index >0.5 non-toxic and non-allergenic properties, and IFN-γ-inducing epitope potential

**Figure S2:** The distribution of CTL epitopes based on percentile rank and antigenicity

**Supplementary Figure S1: The cytokines of immune simulations from HTL epitopes with antigenicity index >0.5 non-toxic and non-allergenic properties, and IFN-γ-inducing epitope potential**

C-ImmSim online server at (http://kraken.iac.rm.cnr.it/C-IMMSIM/) was employed for immune simulations. The HTL epitopes without any adjuvant were respectively injected for three times at intervals of 2 weeks. The results of cytokines are shown as follow:

**HbR_219-233_:**


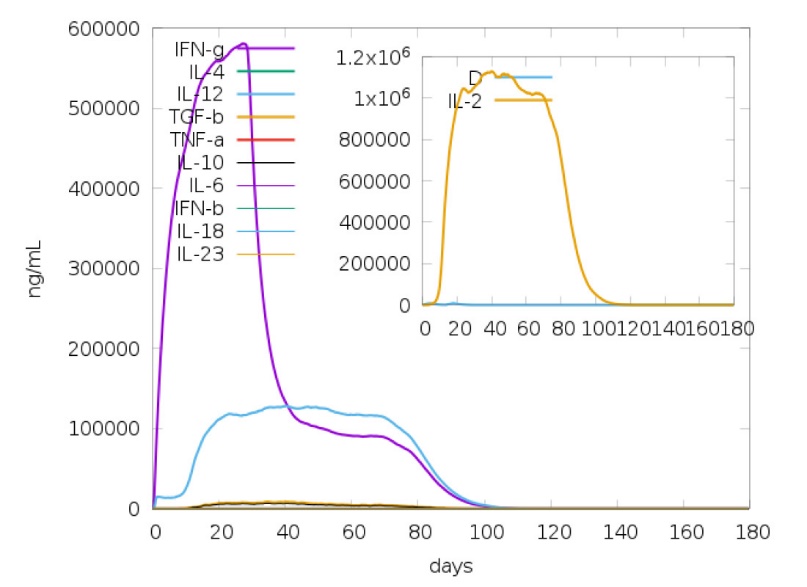


**Supplementary Figure S1-1**. Concentration of cytokines and interleukins analyzed by C-ImmSim online server. HbR_219-233_ epitope without any adjuvant was injected for three times at intervals of 2 weeks. IFN-gamma (IFN-γ), TGF-b (TGF-β), IL-10, and IL-12 are shown in purple line, yellow thick line, black line, and blue thick line respectively. IL-2 and danger signal (D) are presented in yellow thick line and blue thick line in the insert plot respectively.

**HbR_218-232_:**


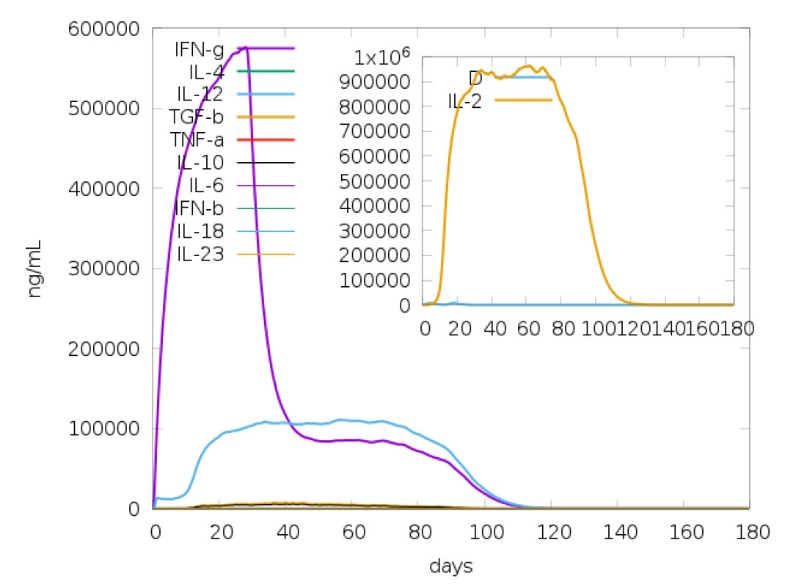


**Supplementary Figure S1-2**. Concentration of cytokines and interleukins analyzed by C-ImmSim online server. HbR_218-232_ epitope without any adjuvant was injected for three times at intervals of 2 weeks. IFN-gamma (IFN-γ), TGF-b (TGF-β), IL-10, and IL-12 are shown in purple line, yellow thick line, black line, and blue thick line respectively. IL-2 and danger signal (D) are presented in yellow thick line and blue thick line in the insert plot respectively.

**HbR_70-84_:**

**
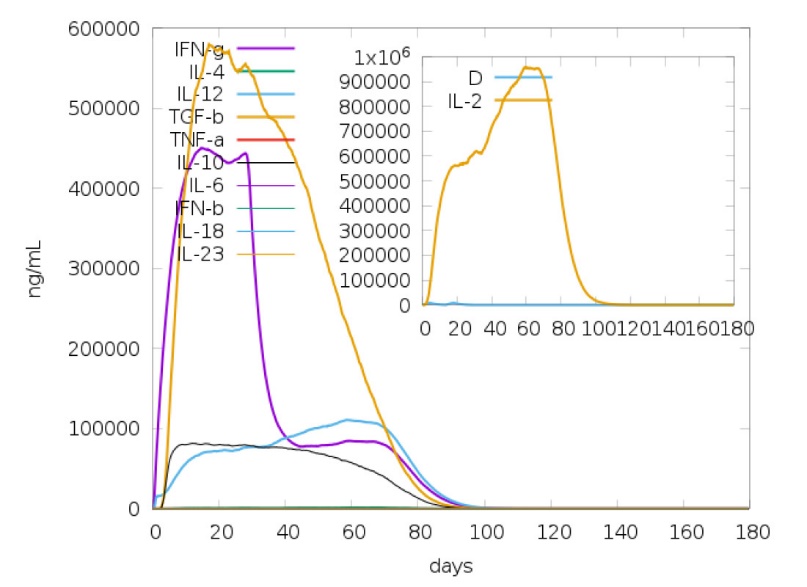
**

**Supplementary Figure S1-3**. Concentration of cytokines and interleukins analyzed by C-ImmSim online server. HbR_70-84_ epitope without any adjuvant was injected for three times at intervals of 2 weeks. IFN-gamma (IFN-γ), TGF-b (TGF-β), IL-10, and IL-12 are shown in purple line, yellow thick line, black line, and blue thick line respectively. IL-2 and danger signal (D) are presented in yellow thick line and blue thick line in the insert plot respectively.

**HbR_221-235_:**


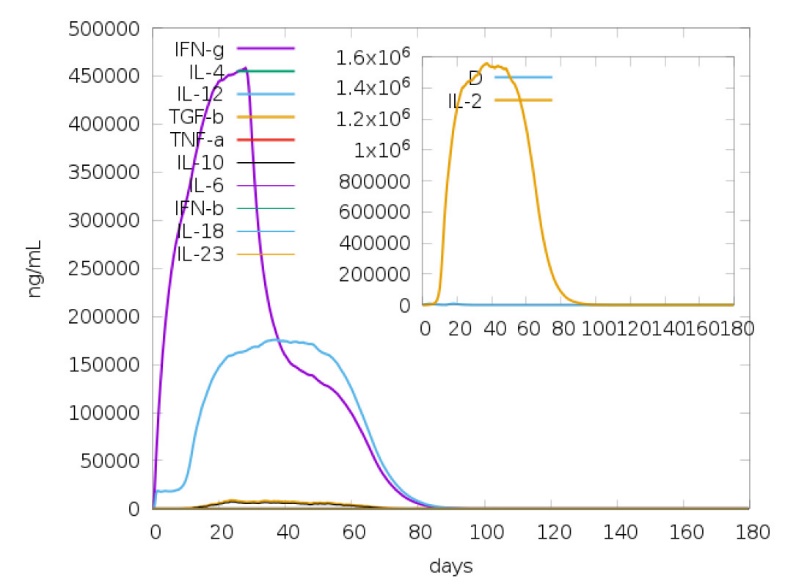


**Supplementary Figure S1-4**. Concentration of cytokines and interleukins analyzed by C-ImmSim online server. HbR_221-235_ epitope without any adjuvant was injected for three times at intervals of 2 weeks. IFN-gamma (IFN-γ), TGF-b (TGF-β), IL-10, and IL-12 are shown in purple line, yellow thick line, black line, and blue thick line respectively. IL-2 and danger signal (D) are presented in yellow thick line and blue thick line in the insert plot respectively.

**HbR_215-229_:**


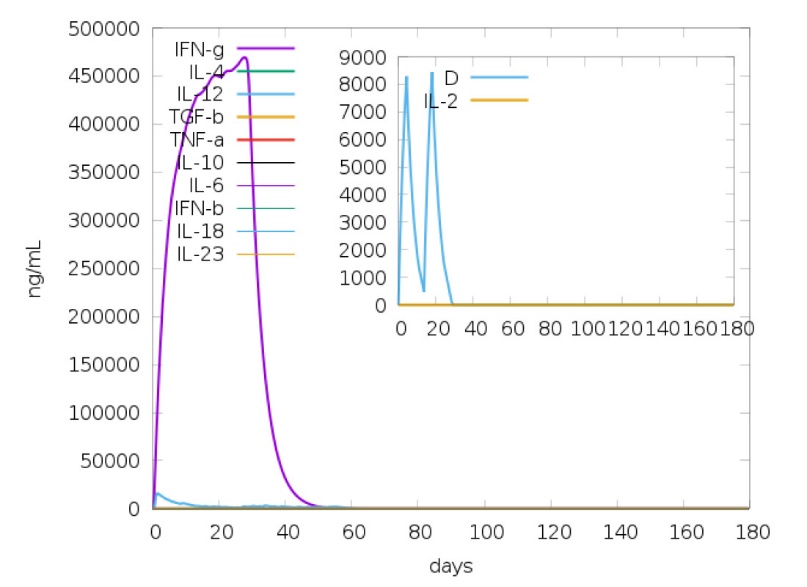


**Supplementary Figure S1-5**. Concentration of cytokines and interleukins analyzed by C-ImmSim online server. HbR_215-229_ epitope without any adjuvant was injected for three times at intervals of 2 weeks. IFN-gamma (IFN-γ), TGF-b (TGF-β), IL-10, and IL-12 are shown in purple line, yellow thick line, black line, and blue thick line respectively. IL-2 and danger signal (D) are presented in yellow thick line and blue thick line in the insert plot respectively.

**HbR_217-231_:**


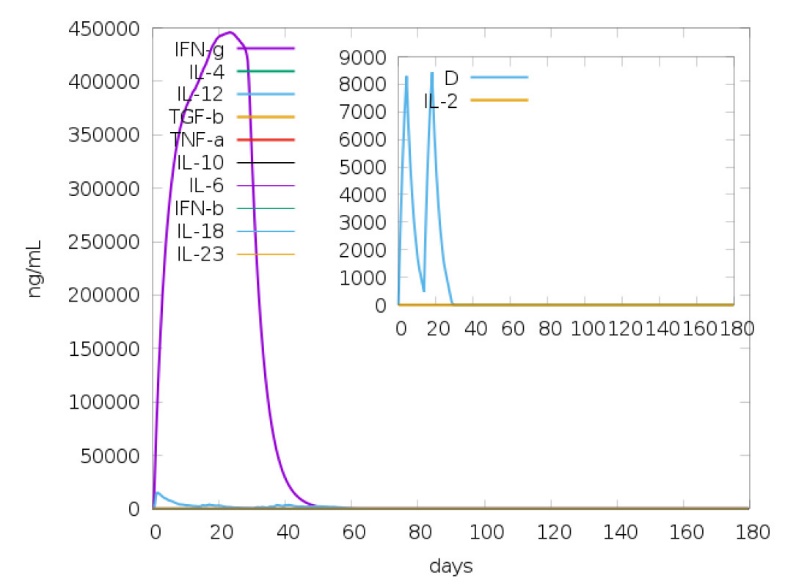


**Supplementary Figure S1-6**. Concentration of cytokines and interleukins analyzed by C-ImmSim online server. HbR_217-231_ epitope without any adjuvant was injected for three times at intervals of 2 weeks. IFN-gamma (IFN-γ), TGF-b (TGF-β), IL-10, and IL-12 are shown in purple line, yellow thick line, black line, and blue thick line respectively. IL-2 and danger signal (D) are presented in yellow thick line and blue thick line in the insert plot respectively.

**HbR_69-83_:**


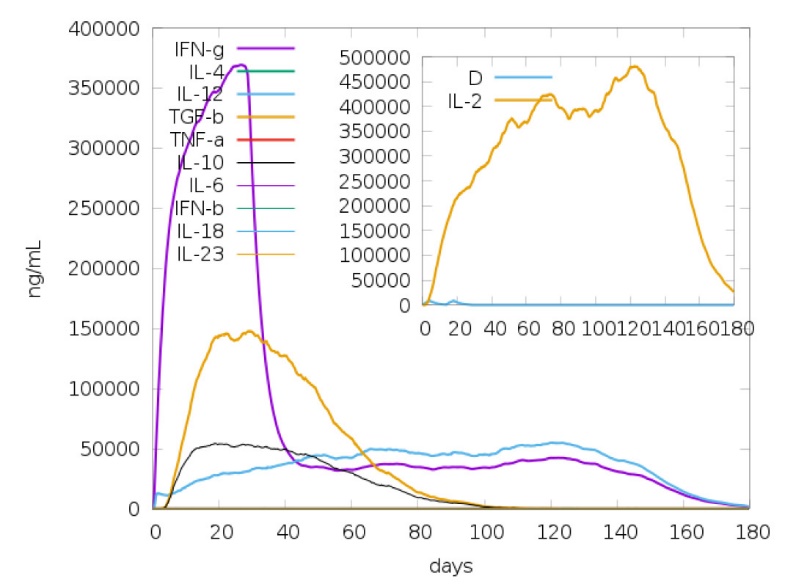


**Supplementary Figure S1-7**. Concentration of cytokines and interleukins analyzed by C-ImmSim online server. HbR_69-83_ epitope without any adjuvant was injected for three times at intervals of 2 weeks. IFN-gamma (IFN-γ), TGF-b (TGF-β), IL-10, and IL-12 are shown in purple line, yellow thick line, black line, and blue thick line respectively. IL-2 and danger signal (D) are presented in yellow thick line and blue thick line in the insert plot respectively.

**HbR_71-85_:**

**_
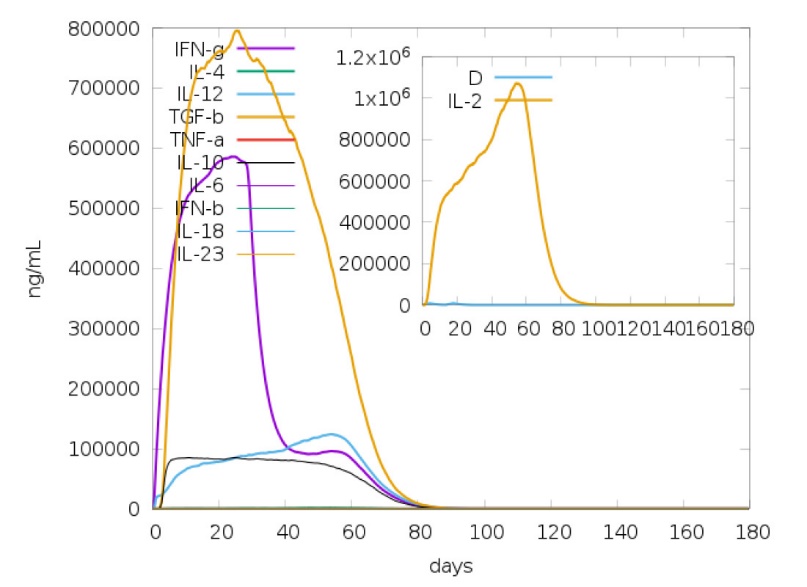
_**

**Supplementary Figure S1-8**. Concentration of cytokines and interleukins analyzed by C-ImmSim online server. HbR_71-85_ epitope without any adjuvant was injected for three times at intervals of 2 weeks. IFN-gamma (IFN-γ), TGF-b (TGF-β), IL-10, and IL-12 are shown in purple line, yellow thick line, black line, and blue thick line respectively. IL-2 and danger signal (D) are presented in yellow thick line and blue thick line in the insert plot respectively.

**HbR_72-86_:**


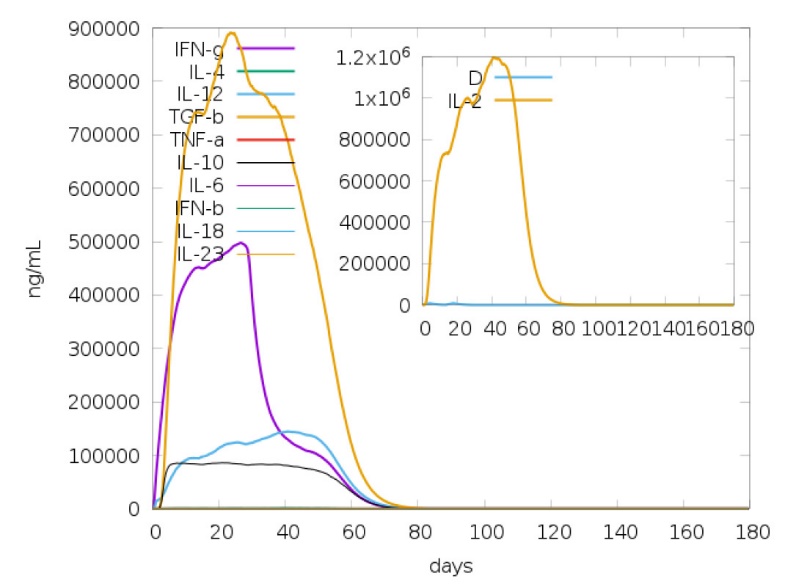


**Supplementary Figure S1-9**. Concentration of cytokines and interleukins analyzed by C-ImmSim online server. HbR_72-86_ epitope without any adjuvant was injected for three times at intervals of 2 weeks. IFN-gamma (IFN-γ), TGF-b (TGF-β), IL-10, and IL-12 are shown in purple line, yellow thick line, black line, and blue thick line respectively. IL-2 and danger signal (D) are presented in yellow thick line and blue thick line in the insert plot respectively.

**HbR_246-260_:**

**_
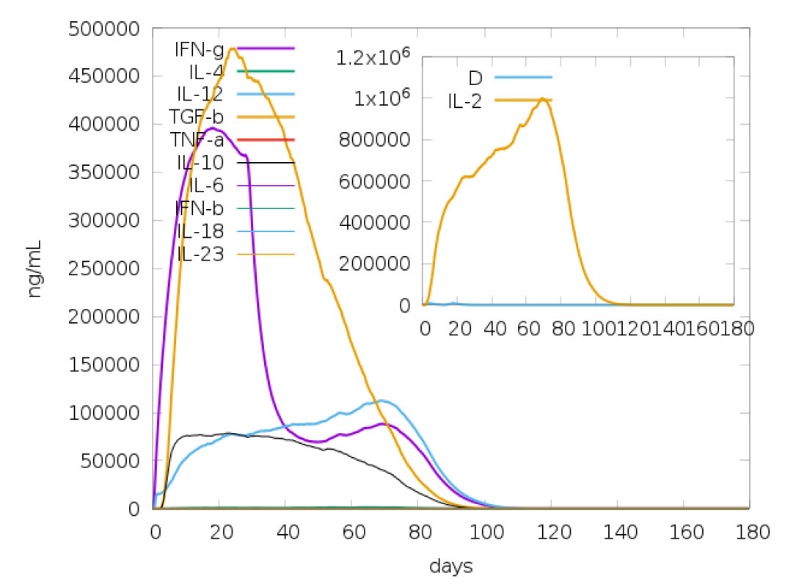
_**

**Supplementary Figure S1-10**. Concentration of cytokines and interleukins analyzed by C-ImmSim online server. HbR_246-260_ epitope without any adjuvant was injected for three times at intervals of 2 weeks. IFN-gamma (IFN-γ), TGF-b (TGF-β), IL-10, and IL-12 are shown in purple line, yellow thick line, black line, and blue thick line respectively. IL-2 and danger signal (D) are presented in yellow thick line and blue thick line in the insert plot respectively.

**HbR_396-410_:**


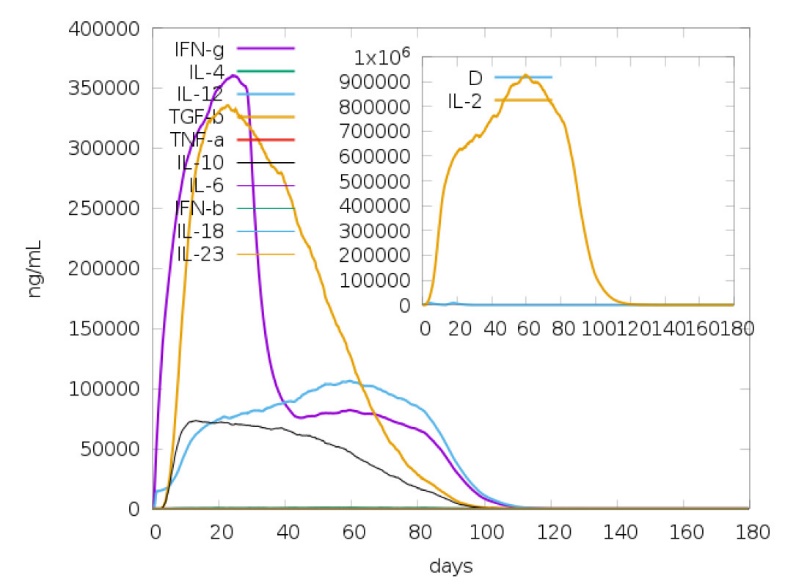


**Supplementary Figure S1-11**. Concentration of cytokines and interleukins analyzed by C-ImmSim online server. HbR_396-410_ epitope without any adjuvant was injected for three times at intervals of 2 weeks. IFN-gamma (IFN-γ), TGF-b (TGF-β), IL-10, and IL-12 are shown in purple line, yellow thick line, black line, and blue thick line respectively. IL-2 and danger signal (D) are presented in yellow thick line and blue thick line in the insert plot respectively.

**HbR_291-305_:**


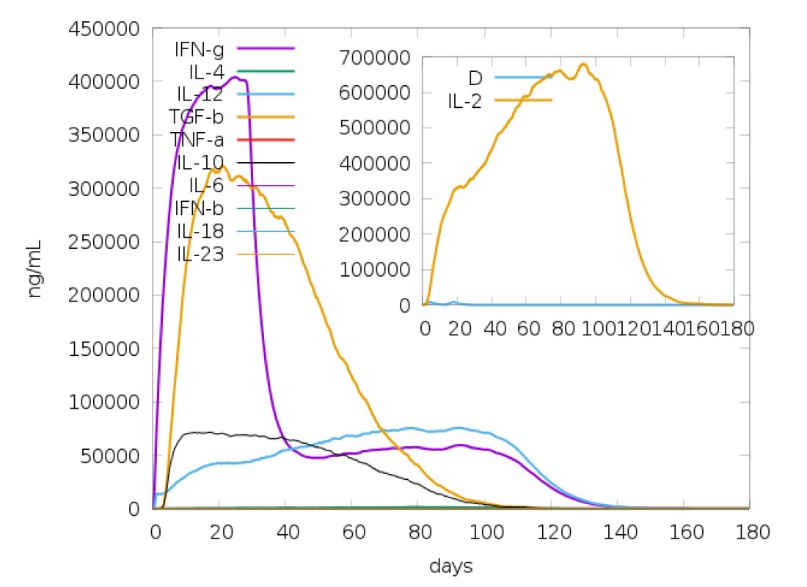


**Supplementary Figure S1-12**. Concentration of cytokines and interleukins analyzed by C-ImmSim online server. HbR_291-305_ epitope without any adjuvant was injected for three times at intervals of 2 weeks. IFN-gamma (IFN-γ), TGF-b (TGF-β), IL-10, and IL-12 are shown in purple line, yellow thick line, black line, and blue thick line respectively. IL-2 and danger signal (D) are presented in yellow thick line and blue thick line in the insert plot respectively.

**HbR_248-262_:**


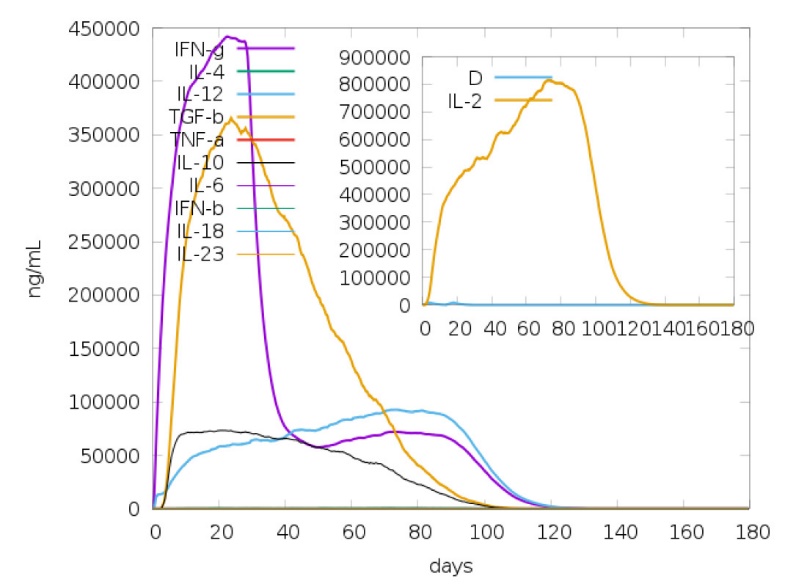


**Supplementary Figure S1-13**. Concentration of cytokines and interleukins analyzed by C-ImmSim online server. HbR_248-262_ epitope without any adjuvant was injected for three times at intervals of 2 weeks. IFN-gamma (IFN-γ), TGF-b (TGF-β), IL-10, and IL-12 are shown in purple line, yellow thick line, black line, and blue thick line respectively. IL-2 and danger signal (D) are presented in yellow thick line and blue thick line in the insert plot respectively.

**HbR_391-405_:**


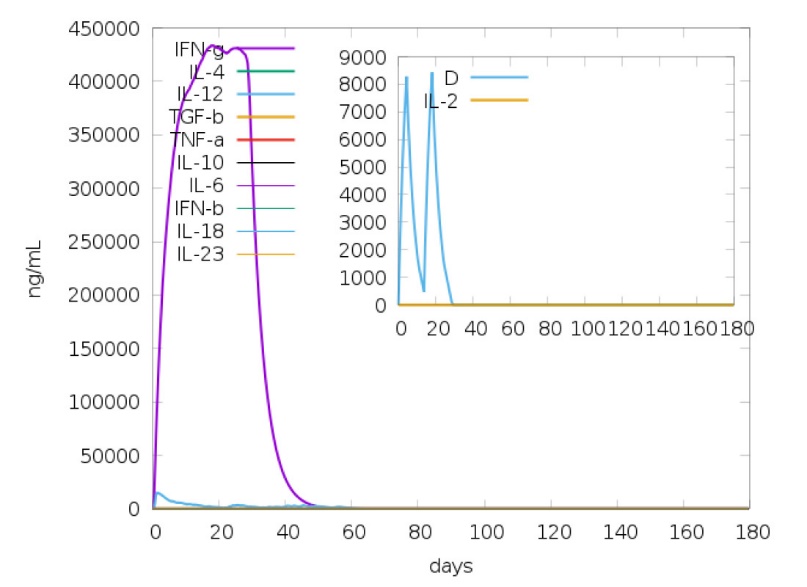


**Supplementary Figure S1-14**. Concentration of cytokines and interleukins analyzed by C-ImmSim online server. HbR_391-405_ epitope without any adjuvant was injected for three times at intervals of 2 weeks. IFN-gamma (IFN-γ), TGF-b (TGF-β), IL-10, and IL-12 are shown in purple line, yellow thick line, black line, and blue thick line respectively. IL-2 and danger signal (D) are presented in yellow thick line and blue thick line in the insert plot respectively.

**HbR_276-290_:**


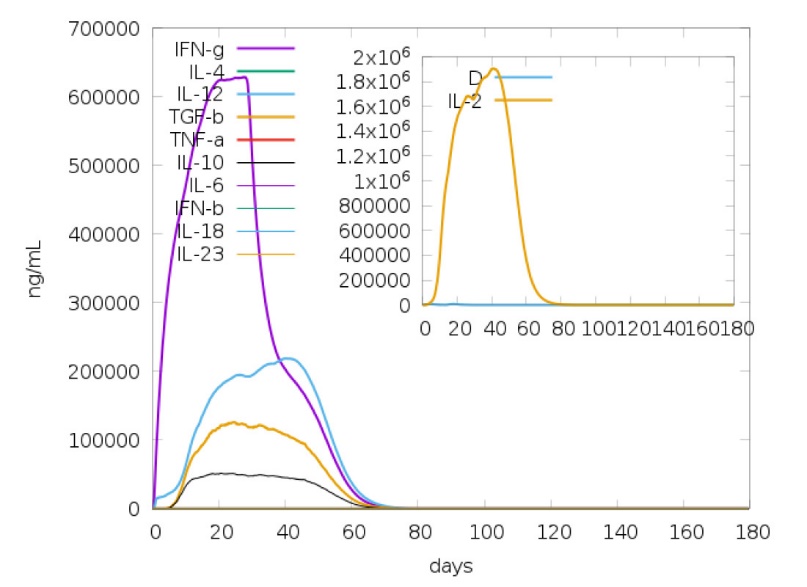


**Supplementary Figure S1-15**. Concentration of cytokines and interleukins analyzed by C-ImmSim online server. HbR_276-290_ epitope without any adjuvant was injected for three times at intervals of 2 weeks. IFN-gamma (IFN-γ), TGF-b (TGF-β), IL-10, and IL-12 are shown in purple line, yellow thick line, black line, and blue thick line respectively. IL-2 and danger signal (D) are presented in yellow thick line and blue thick line in the insert plot respectively.

**HbR_251-265_:**


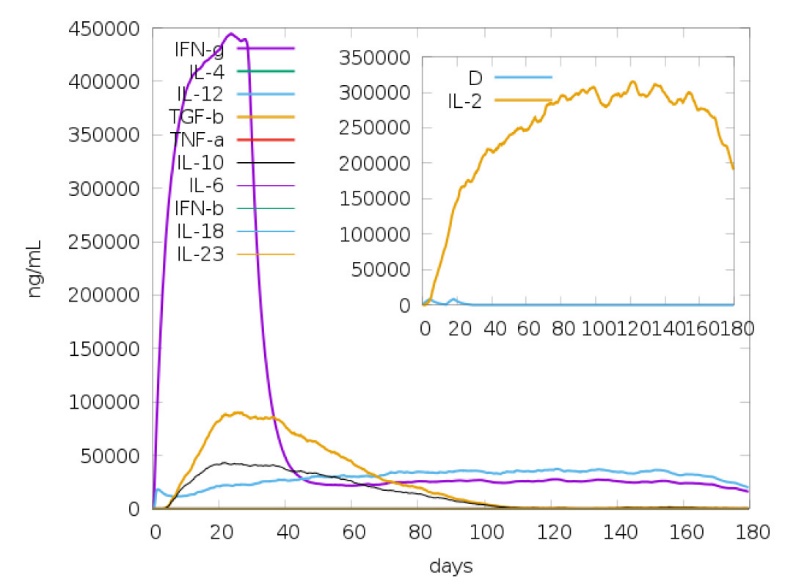


**Supplementary Figure S1-16**. Concentration of cytokines and interleukins analyzed by C-ImmSim online server. HbR_251-265_ epitope without any adjuvant was injected for three times at intervals of 2 weeks. IFN-gamma (IFN-γ), TGF-b (TGF-β), IL-10, and IL-12 are shown in purple line, yellow thick line, black line, and blue thick line respectively. IL-2 and danger signal (D) are presented in yellow thick line and blue thick line in the insert plot respectively.

**HbR_255-269_:**


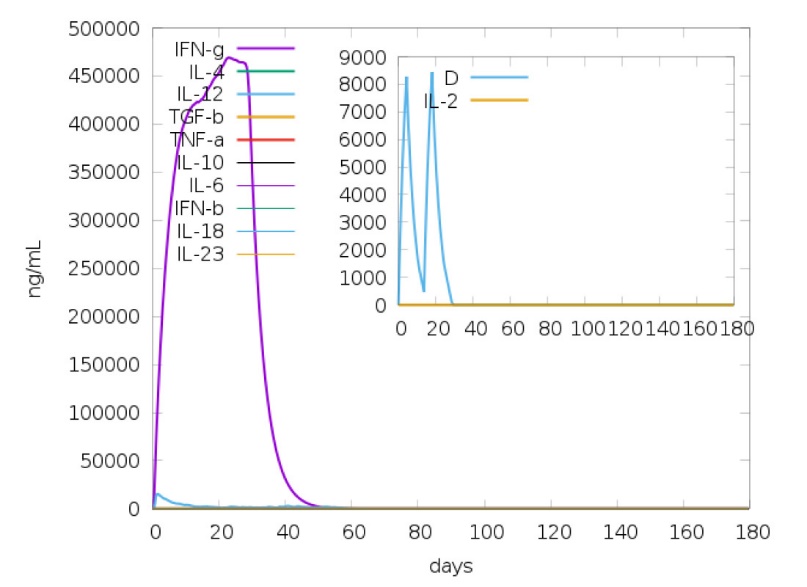


**Supplementary Figure S1-17**. Concentration of cytokines and interleukins analyzed by C-ImmSim online server. HbR_255-269_ epitope without any adjuvant was injected for three times at intervals of 2 weeks. IFN-gamma (IFN-γ), TGF-b (TGF-β), IL-10, and IL-12 are shown in purple line, yellow thick line, black line, and blue thick line respectively. IL-2 and danger signal (D) are presented in yellow thick line and blue thick line in the insert plot respectively.

**HbR_249-263_:**


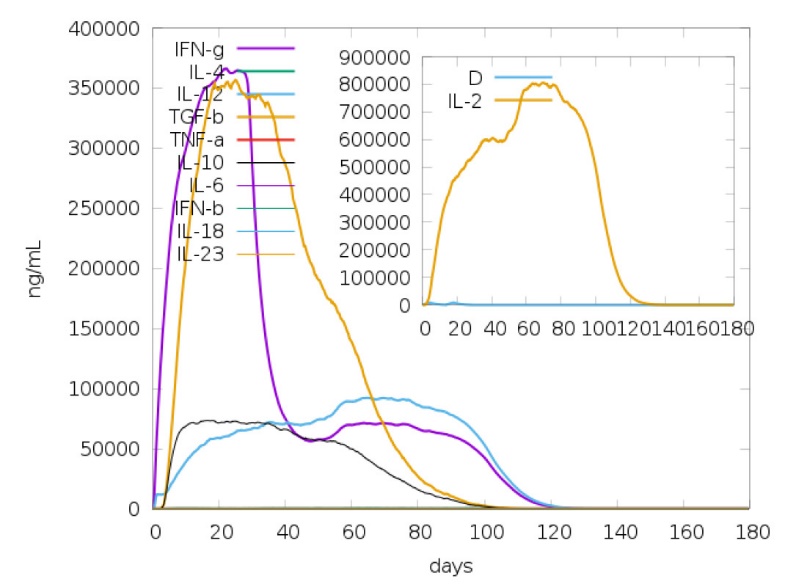


**Supplementary Figure S1-18**. Concentration of cytokines and interleukins analyzed by C-ImmSim online server. HbR_249-263_ epitope without any adjuvant was injected for three times at intervals of 2 weeks. IFN-gamma (IFN-γ), TGF-b (TGF-β), IL-10, and IL-12 are shown in purple line, yellow thick line, black line, and blue thick line respectively. IL-2 and danger signal (D) are presented in yellow thick line and blue thick line in the insert plot respectively.

**HbR_250-264_:**


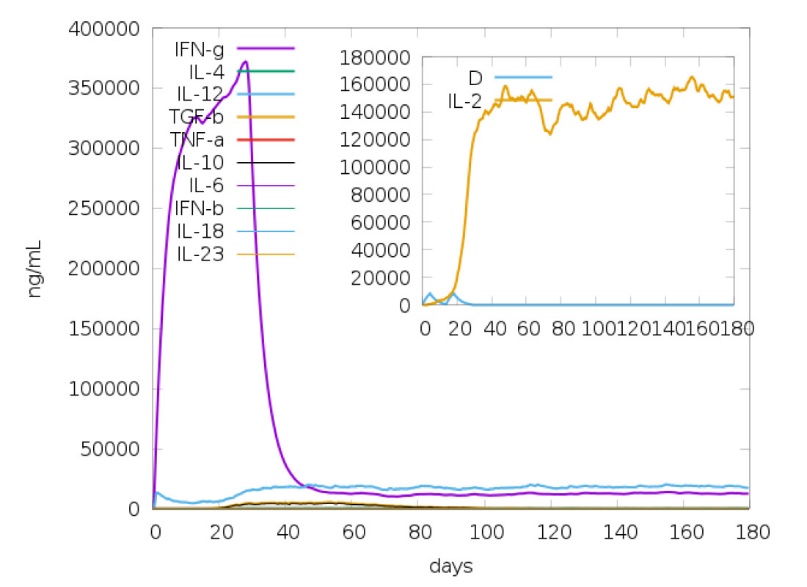


**Supplementary Figure S1-19**. Concentration of cytokines and interleukins analyzed by C-ImmSim online server. HbR_250-264_ epitope without any adjuvant was injected for three times at intervals of 2 weeks. IFN-gamma (IFN-γ), TGF-b (TGF-β), IL-10, and IL-12 are shown in purple line, yellow thick line, black line, and blue thick line respectively. IL-2 and danger signal (D) are presented in yellow thick line and blue thick line in the insert plot respectively.

**KMP-11_43-57_:**


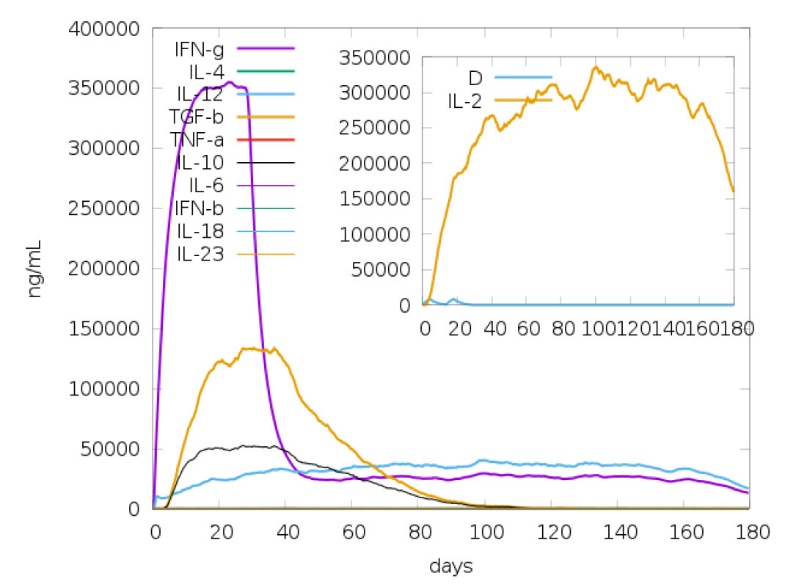


**Supplementary Figure S1-20**. Concentration of cytokines and interleukins analyzed by C-ImmSim online server. KMP-11_43-57_ epitope without any adjuvant was injected for three times at intervals of 2 weeks. IFN-gamma (IFN-γ), TGF-b (TGF-β), IL-10, and IL-12 are shown in purple line, yellow thick line, black line, and blue thick line respectively. IL-2 and danger signal (D) are presented in yellow thick line and blue thick line in the insert plot respectively.

**KMP-11_47-61_:**


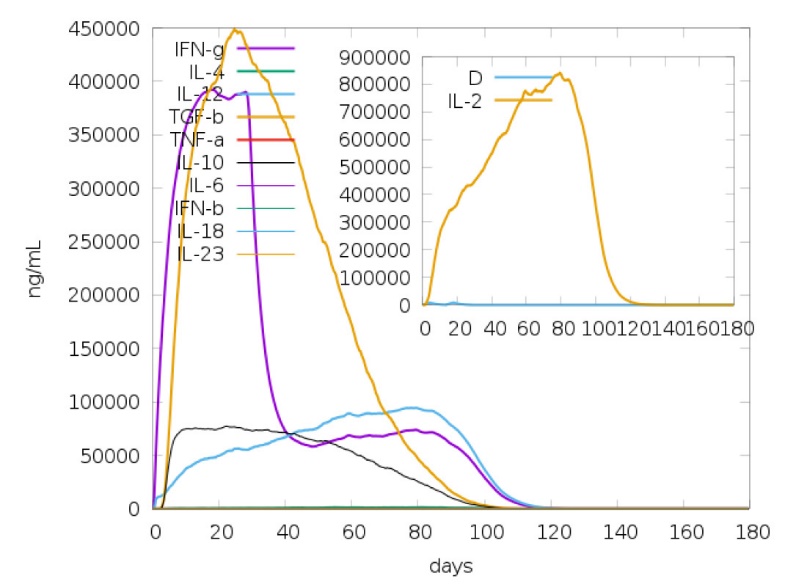


**Supplementary Figure S1-21**. Concentration of cytokines and interleukins analyzed by C-ImmSim online server. KMP-11_47-61_ epitope without any adjuvant was injected for three times at intervals of 2 weeks. IFN-gamma (IFN-γ), TGF-b (TGF-β), IL-10, and IL-12 are shown in purple line, yellow thick line, black line, and blue thick line respectively. IL-2 and danger signal (D) are presented in yellow thick line and blue thick line in the insert plot respectively.

**KMP-11_44-58_:**

**_
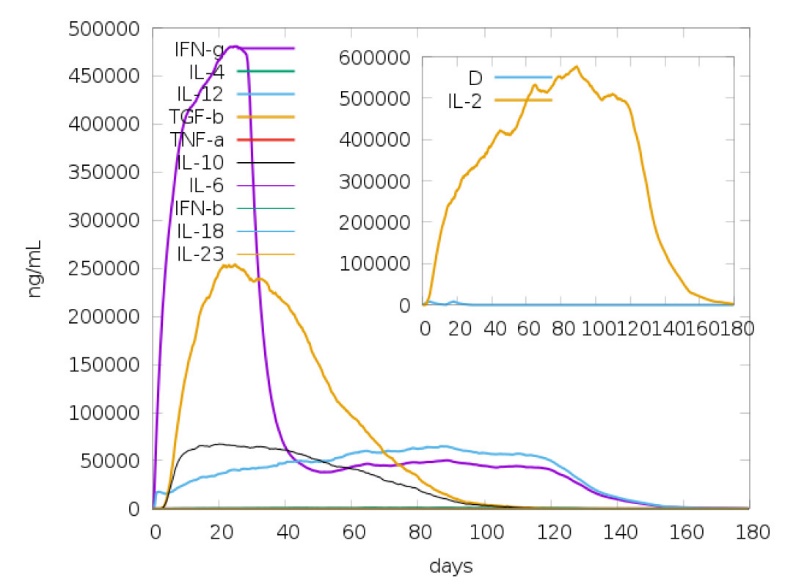
_**

**Supplementary Figure S1-22**. Concentration of cytokines and interleukins analyzed by C-ImmSim online server. KMP-11_44-58_ epitope without any adjuvant was injected for three times at intervals of 2 weeks. IFN-gamma (IFN-γ), TGF-b (TGF-β), IL-10, and IL-12 are shown in purple line, yellow thick line, black line, and blue thick line respectively. IL-2 and danger signal (D) are presented in yellow thick line and blue thick line in the insert plot respectively.

**KMP-11_25-39_:**


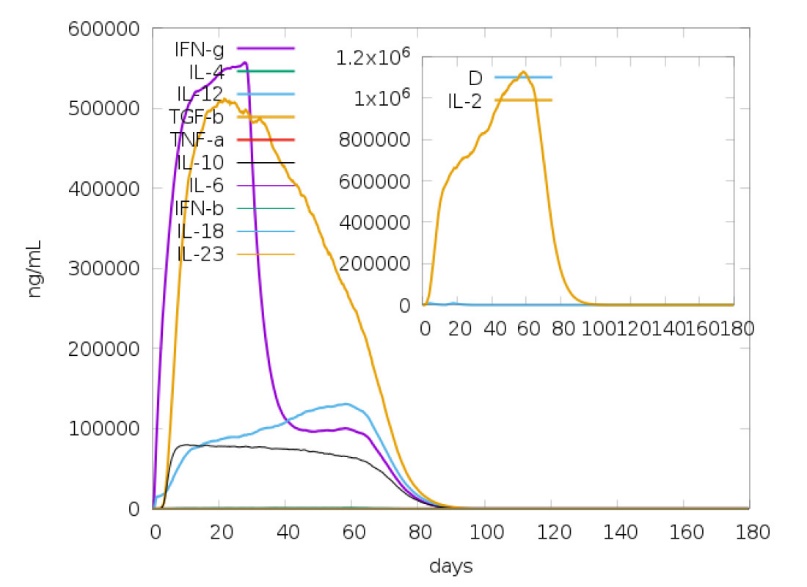


**Supplementary Figure S1-23**. Concentration of cytokines and interleukins analyzed by C-ImmSim online server. KMP-11_25-39_ epitope without any adjuvant was injected for three times at intervals of 2 weeks. IFN-gamma (IFN-γ), TGF-b (TGF-β), IL-10, and IL-12 are shown in purple line, yellow thick line, black line, and blue thick line respectively. IL-2 and danger signal (D) are presented in yellow thick line and blue thick line in the insert plot respectively.

**KMP-11_45-59_:**


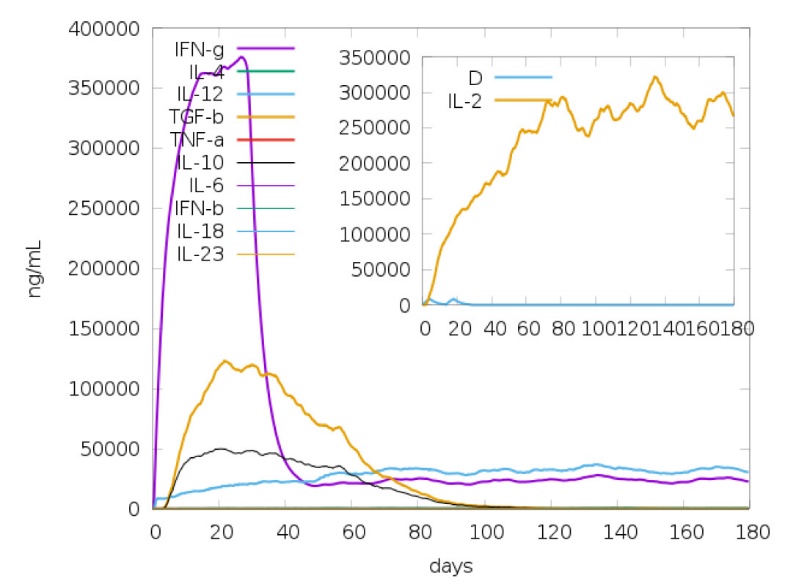


**Supplementary Figure S1-24**. Concentration of cytokines and interleukins analyzed by C-ImmSim online server. KMP-11_45-59_ epitope without any adjuvant was injected for three times at intervals of 2 weeks. IFN-gamma (IFN-γ), TGF-b (TGF-β), IL-10, and IL-12 are shown in purple line, yellow thick line, black line, and blue thick line respectively. IL-2 and danger signal (D) are presented in yellow thick line and blue thick line in the insert plot respectively.

**KMP-11_27-41_:**

**_
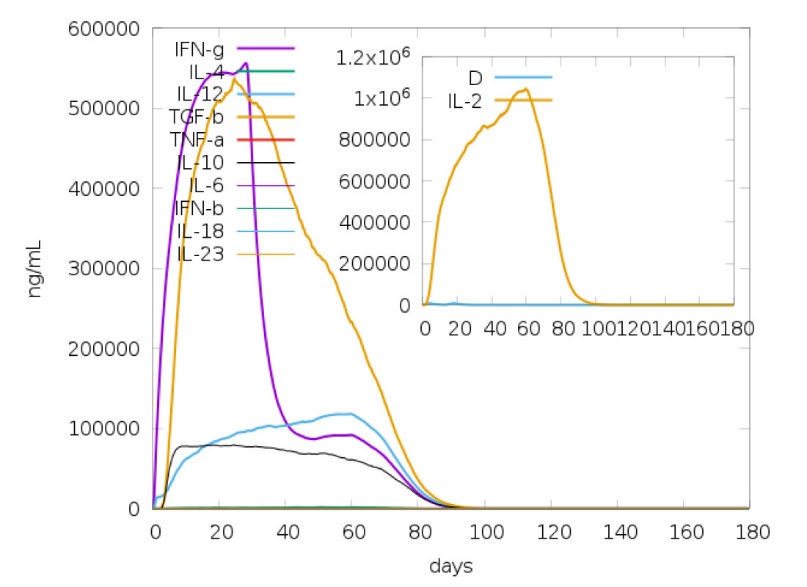
_**

**Supplementary Figure S1-25**. Concentration of cytokines and interleukins analyzed by C-ImmSim online server. KMP-11_27-41_ epitope without any adjuvant was injected for three times at intervals of 2 weeks. IFN-gamma (IFN-γ), TGF-b (TGF-β), IL-10, and IL-12 are shown in purple line, yellow thick line, black line, and blue thick line respectively. IL-2 and danger signal (D) are presented in yellow thick line and blue thick line in the insert plot respectively.

**KMP-11_26-40_:**


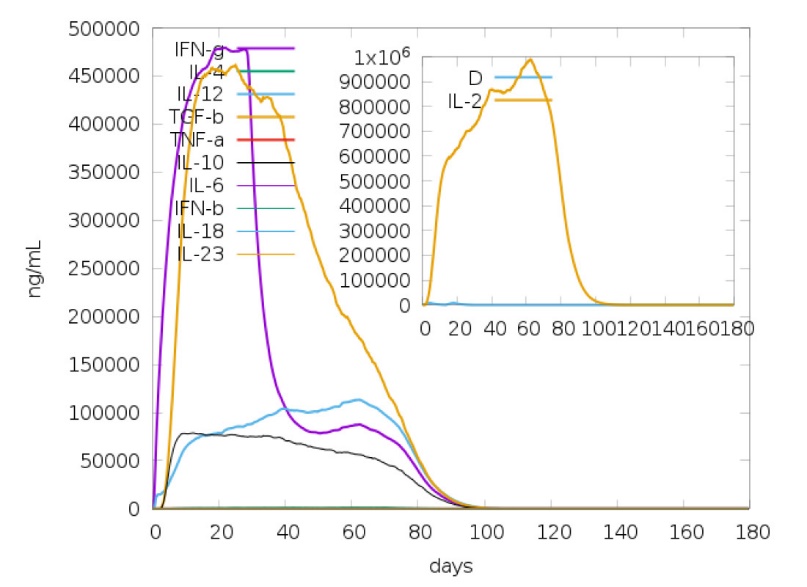


**Supplementary Figure S1-26**. Concentration of cytokines and interleukins analyzed by C-ImmSim online server. KMP-11_26-40_ epitope without any adjuvant was injected for three times at intervals of 2 weeks. IFN-gamma (IFN-γ), TGF-b (TGF-β), IL-10, and IL-12 are shown in purple line, yellow thick line, black line, and blue thick line respectively. IL-2 and danger signal (D) are presented in yellow thick line and blue thick line in the insert plot respectively.

**KMP-11_23-37_:**


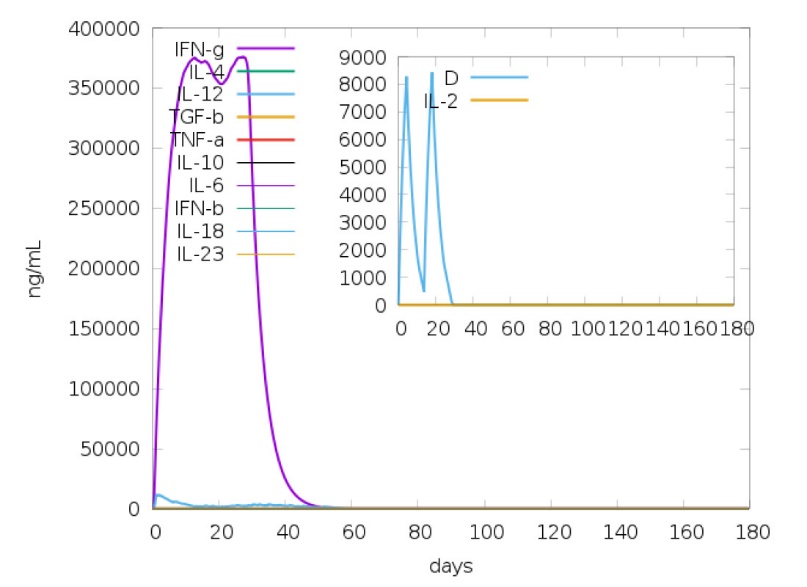


**Supplementary Figure S1-27**. Concentration of cytokines and interleukins analyzed by C-ImmSim online server. KMP-11_23-37_ epitope without any adjuvant was injected for three times at intervals of 2 weeks. IFN-gamma (IFN-γ), TGF-b (TGF-β), IL-10, and IL-12 are shown in purple line, yellow thick line, black line, and blue thick line respectively. IL-2 and danger signal (D) are presented in yellow thick line and blue thick line in the insert plot respectively.

**KMP-11_28-42_:**

**_
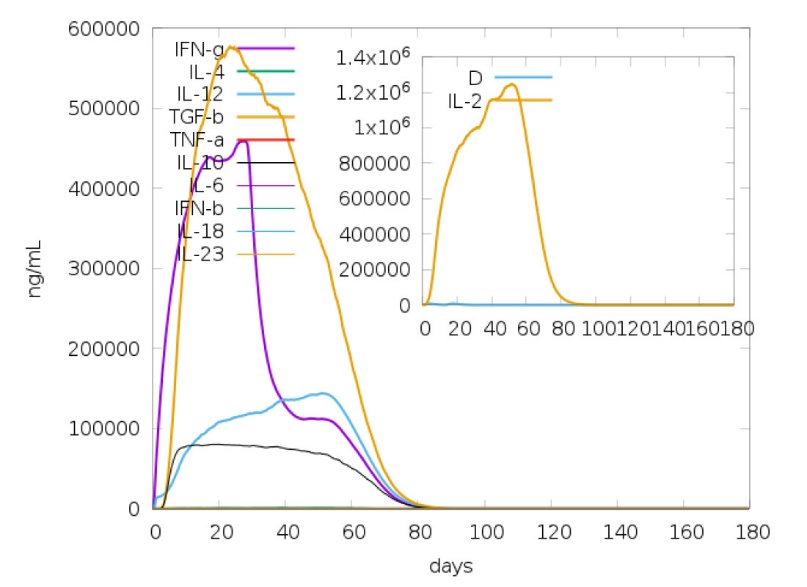
_**

**Supplementary Figure S1-28**. Concentration of cytokines and interleukins analyzed by C-ImmSim online server. KMP-11_28-42_ epitope without any adjuvant was injected for three times at intervals of 2 weeks. IFN-gamma (IFN-γ), TGF-b (TGF-β), IL-10, and IL-12 are shown in purple line, yellow thick line, black line, and blue thick line respectively. IL-2 and danger signal (D) are presented in yellow thick line and blue thick line in the insert plot respectively.

**KMP-11_51-65_:**


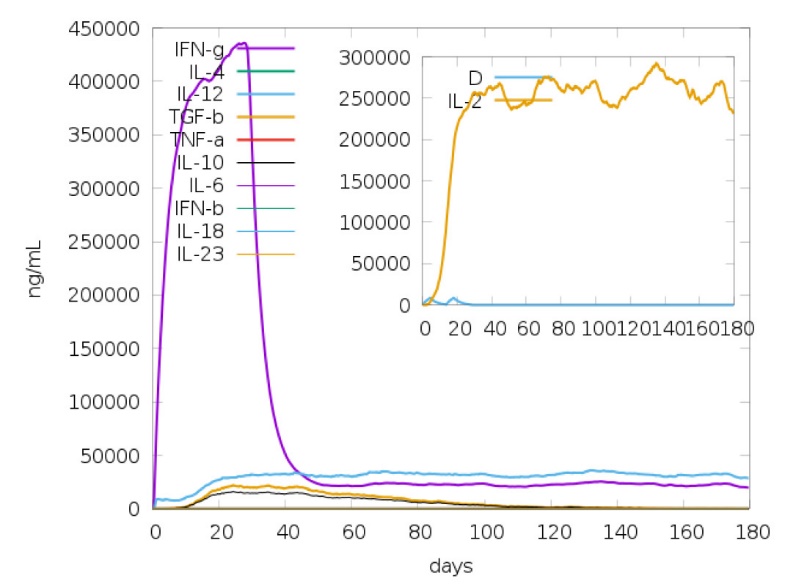


**Supplementary Figure S1-29**. Concentration of cytokines and interleukins analyzed by C-ImmSim online server. KMP-11_51-65_ epitope without any adjuvant was injected for three times at intervals of 2 weeks. IFN-gamma (IFN-γ), TGF-b (TGF-β), IL-10, and IL-12 are shown in purple line, yellow thick line, black line, and blue thick line respectively. IL-2 and danger signal (D) are presented in yellow thick line and blue thick line in the insert plot respectively.

**KMP-11_21-35_:**

**_
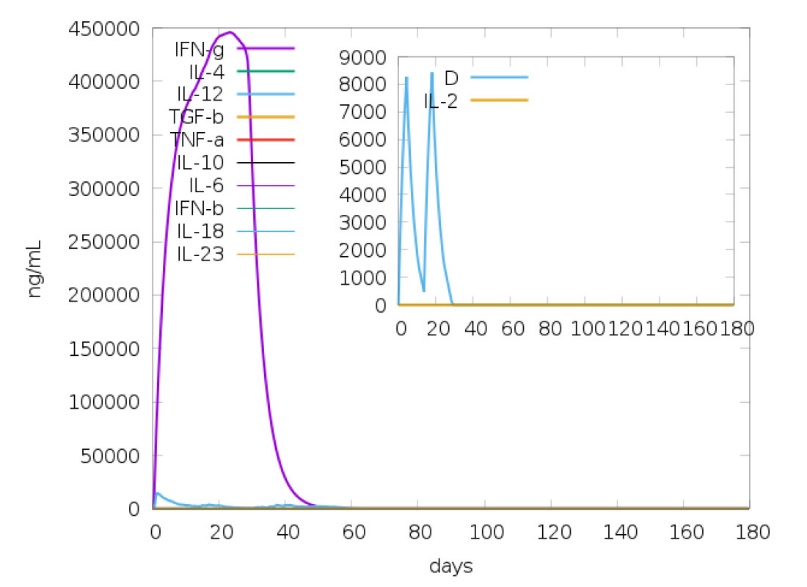
_**

**Supplementary Figure S1-30**. Concentration of cytokines and interleukins analyzed by C-ImmSim online server. KMP-11_21-35_ epitope without any adjuvant was injected for three times at intervals of 2 weeks. IFN-gamma (IFN-γ), TGF-b (TGF-β), IL-10, and IL-12 are shown in purple line, yellow thick line, black line, and blue thick line respectively. IL-2 and danger signal (D) are presented in yellow thick line and blue thick line in the insert plot respectively.

**KMP-11_29-43_:**


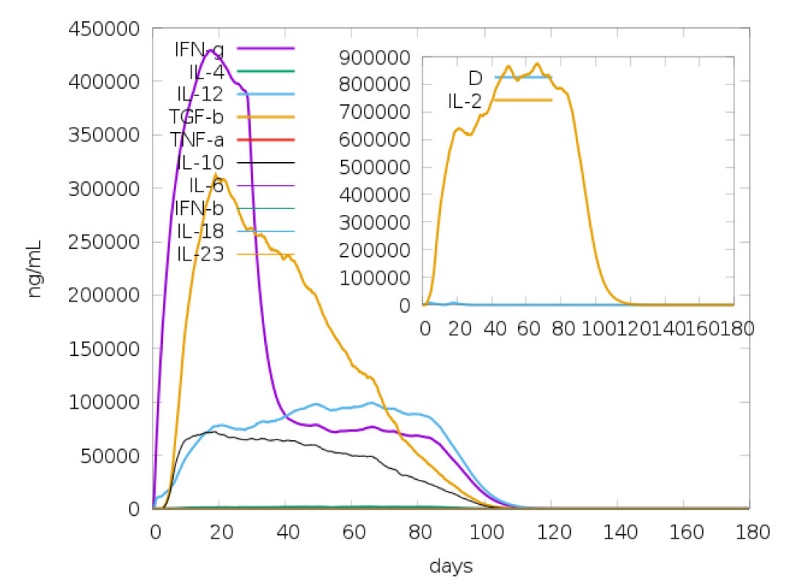


**Supplementary Figure S1-31**. Concentration of cytokines and interleukins analyzed by C-ImmSim online server. KMP-11_29-43_ epitope without any adjuvant was injected for three times at intervals of 2 weeks. IFN-gamma (IFN-γ), TGF-b (TGF-β), IL-10, and IL-12 are shown in purple line, yellow thick line, black line, and blue thick line respectively. IL-2 and danger signal (D) are presented in yellow thick line and blue thick line in the insert plot respectively.

**Gp63_282-296_:**


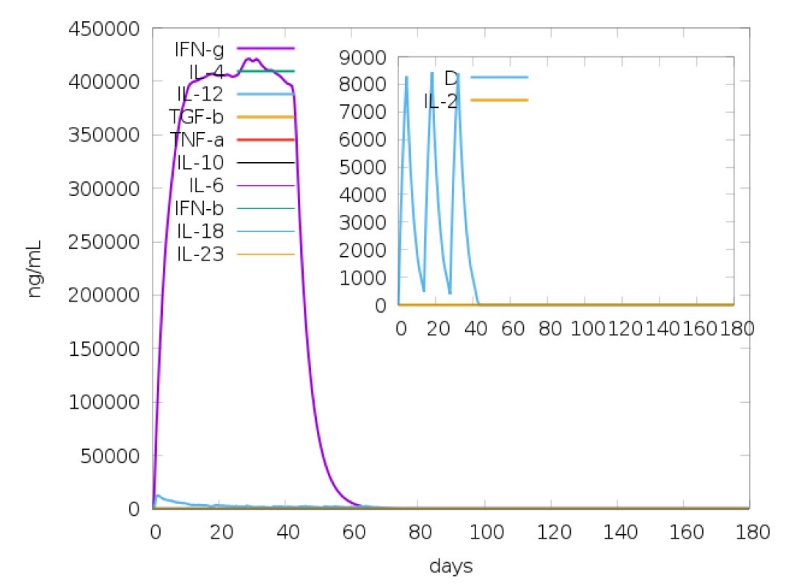


**Supplementary Figure S1-32**. Concentration of cytokines and interleukins analyzed by C-ImmSim online server. Gp63_282-296_ epitope without any adjuvant was injected for three times at intervals of 2 weeks. IFN-gamma (IFN-γ), TGF-b (TGF-β), IL-10, and IL-12 are shown in purple line, yellow thick line, black line, and blue thick line respectively. IL-2 and danger signal (D) are presented in yellow thick line and blue thick line in the insert plot respectively.

**Gp63_490-504_:**


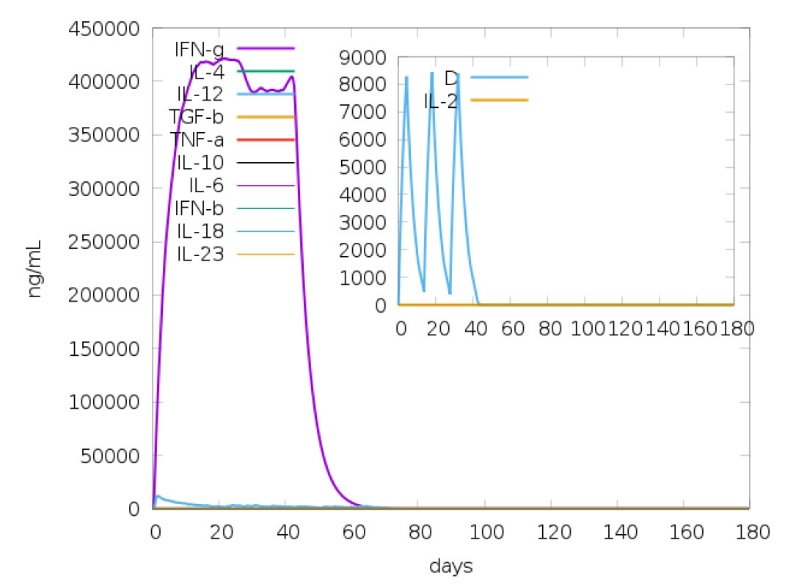


**Supplementary Figure S1-33**. Concentration of cytokines and interleukins analyzed by C-ImmSim online server. Gp63_490-504_ epitope without any adjuvant was injected for three times at intervals of 2 weeks. IFN-gamma (IFN-γ), TGF-b (TGF-β), IL-10, and IL-12 are shown in purple line, yellow thick line, black line, and blue thick line respectively. IL-2 and danger signal (D) are presented in yellow thick line and blue thick line in the insert plot respectively.

**Gp63_264-278_:**

**_
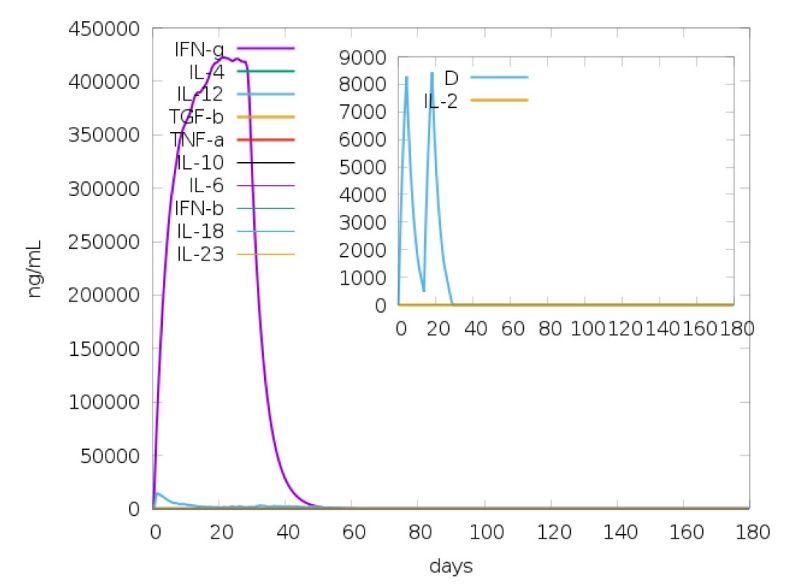
_**

**Supplementary Figure S1-34**. Concentration of cytokines and interleukins analyzed by C-ImmSim online server. Gp63_264-278_ epitope without any adjuvant was injected for three times at intervals of 2 weeks. IFN-gamma (IFN-γ), TGF-b (TGF-β), IL-10, and IL-12 are shown in purple line, yellow thick line, black line, and blue thick line respectively. IL-2 and danger signal (D) are presented in yellow thick line and blue thick line in the insert plot respectively.

**Gp63_206-220_:**


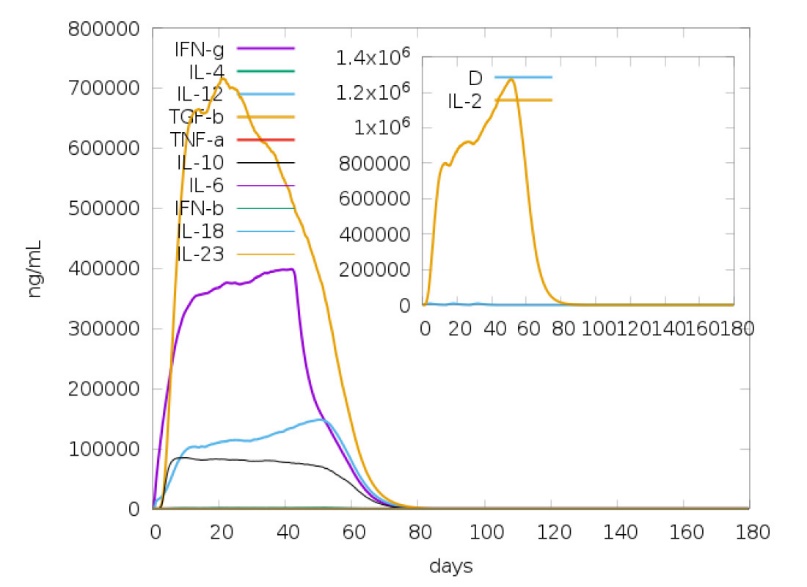


**Supplementary Figure S1-35**. Concentration of cytokines and interleukins analyzed by C-ImmSim online server. Gp63_206-220_ epitope without any adjuvant was injected for three times at intervals of 2 weeks. IFN-gamma (IFN-γ), TGF-b (TGF-β), IL-10, and IL-12 are shown in purple line, yellow thick line, black line, and blue thick line respectively. IL-2 and danger signal (D) are presented in yellow thick line and blue thick line in the insert plot respectively.

**Gp63_205-219_:**

**
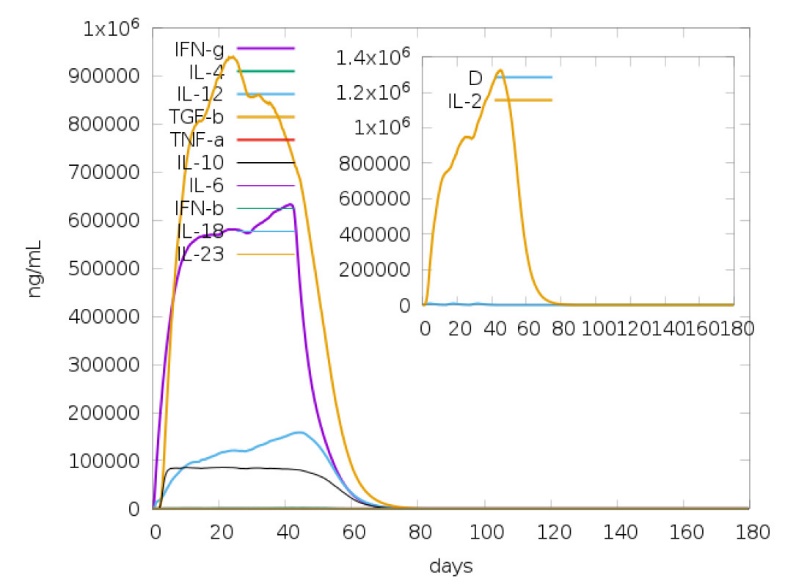
**

**Supplementary Figure S1-36**. Concentration of cytokines and interleukins analyzed by C-ImmSim online server. Gp63_205-219_ epitope without any adjuvant was injected for three times at intervals of 2 weeks. IFN-gamma (IFN-γ), TGF-b (TGF-β), IL-10, and IL-12 are shown in purple line, yellow thick line, black line, and blue thick line respectively. IL-2 and danger signal (D) are presented in yellow thick line and blue thick line in the insert plot respectively.

**Gp63_155-169_:**

**_
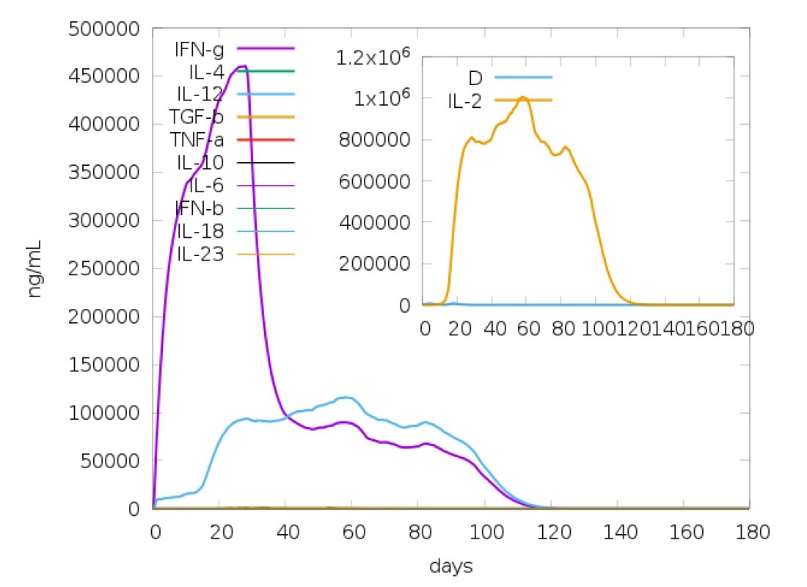
_**

**Supplementary Figure S1-37**. Concentration of cytokines and interleukins analyzed by C-ImmSim online server. Gp63_155-169_ epitope without any adjuvant was injected for three times at intervals of 2 weeks. IFN-gamma (IFN-γ), TGF-b (TGF-β), IL-10, and IL-12 are shown in purple line, yellow thick line, black line, and blue thick line respectively. IL-2 and danger signal (D) are presented in yellow thick line and blue thick line in the insert plot respectively.

**Gp63_204-218_:**

**_
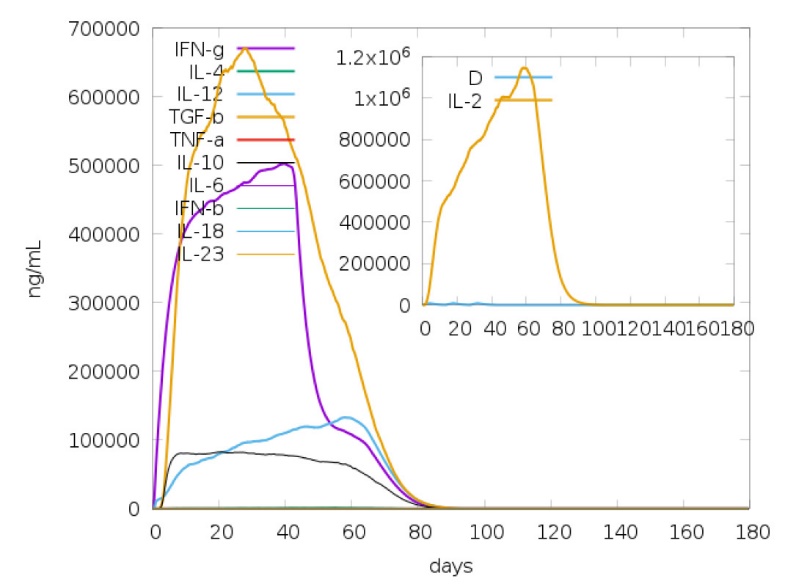
_**

**Supplementary Figure S1-38**. Concentration of cytokines and interleukins analyzed by C-ImmSim online server. Gp63_204-218_ epitope without any adjuvant was injected for three times at intervals of 2 weeks. IFN-gamma (IFN-γ), TGF-b (TGF-β), IL-10, and IL-12 are shown in purple line, yellow thick line, black line, and blue thick line respectively. IL-2 and danger signal (D) are presented in yellow thick line and blue thick line in the insert plot respectively.

**Gp63_63-77_:**

**_
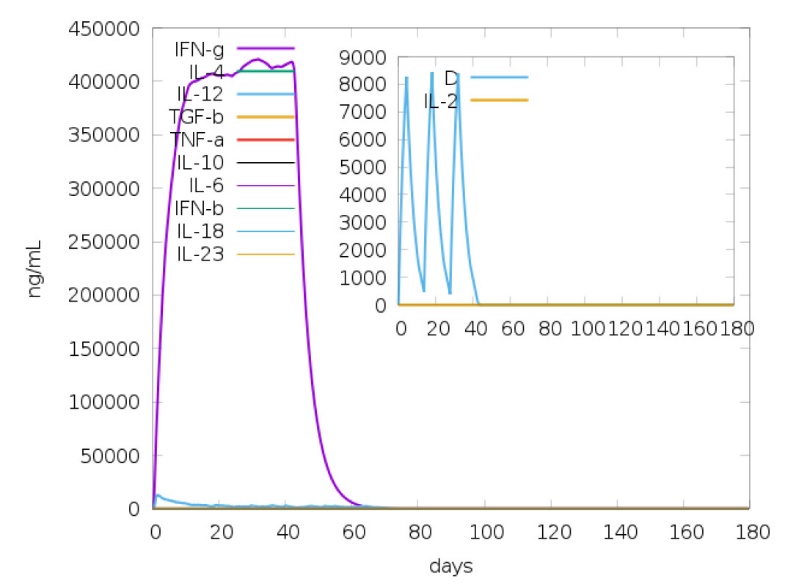
_**

**Supplementary Figure S1-39**. Concentration of cytokines and interleukins analyzed by C-ImmSim online server. Gp63_63-77_ epitope without any adjuvant was injected for three times at intervals of 2 weeks. IFN-gamma (IFN-γ), TGF-b (TGF-β), IL-10, and IL-12 are shown in purple line, yellow thick line, black line, and blue thick line respectively. IL-2 and danger signal (D) are presented in yellow thick line and blue thick line in the insert plot respectively.

**Gp63_263-277_:**


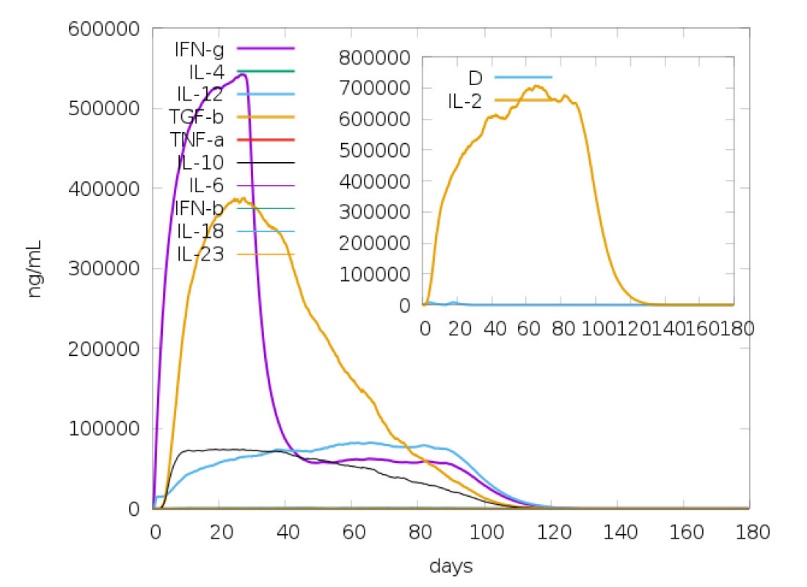


**Supplementary Figure S1-40**. Concentration of cytokines and interleukins analyzed by C-ImmSim online server. Gp63_263-277_ epitope without any adjuvant was injected for three times at intervals of 2 weeks. IFN-gamma (IFN-γ), TGF-b (TGF-β), IL-10, and IL-12 are shown in purple line, yellow thick line, black line, and blue thick line respectively. IL-2 and danger signal (D) are presented in yellow thick line and blue thick line in the insert plot respectively.

**Gp63_275-289_:**

**_
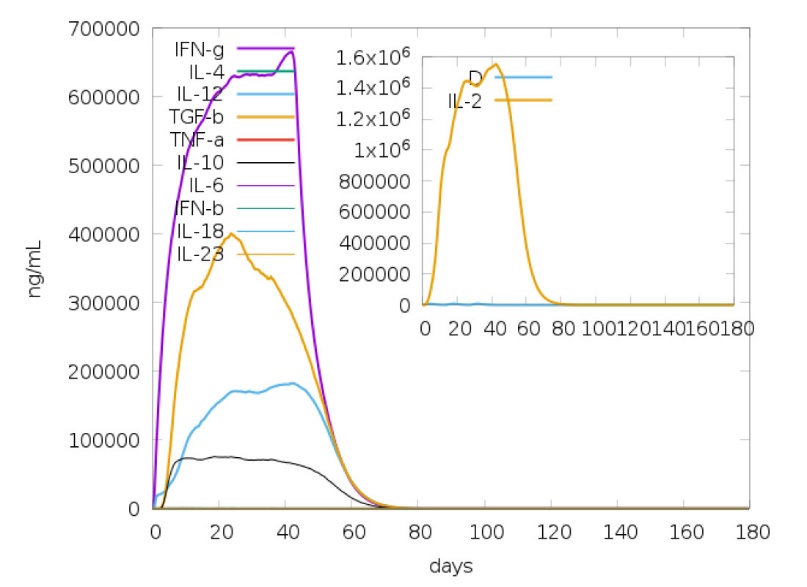
_**

**Supplementary Figure S1-41**. Concentration of cytokines and interleukins analyzed by C-ImmSim online server. Gp63_275-289_ epitope without any adjuvant was injected for three times at intervals of 2 weeks. IFN-gamma (IFN-γ), TGF-b (TGF-β), IL-10, and IL-12 are shown in purple line, yellow thick line, black line, and blue thick line respectively. IL-2 and danger signal (D) are presented in yellow thick line and blue thick line in the insert plot respectively.

**Gp63_56-70_:**

**_
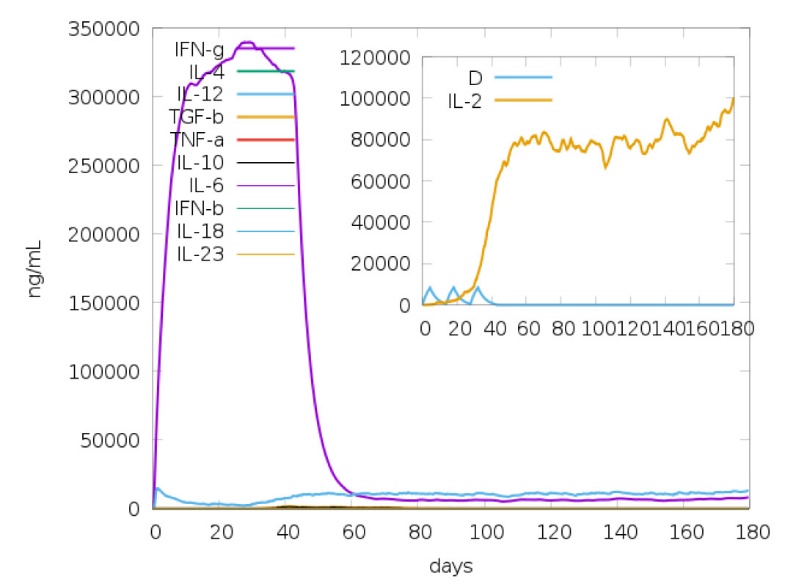
_**

**Supplementary Figure S1-42**. Concentration of cytokines and interleukins analyzed by C-ImmSim online server. Gp63_56-70_ epitope without any adjuvant was injected for three times at intervals of 2 weeks. IFN-gamma (IFN-γ), TGF-b (TGF-β), IL-10, and IL-12 are shown in purple line, yellow thick line, black line, and blue thick line respectively. IL-2 and danger signal (D) are presented in yellow thick line and blue thick line in the insert plot respectively.

**Gp63_159-173_:**


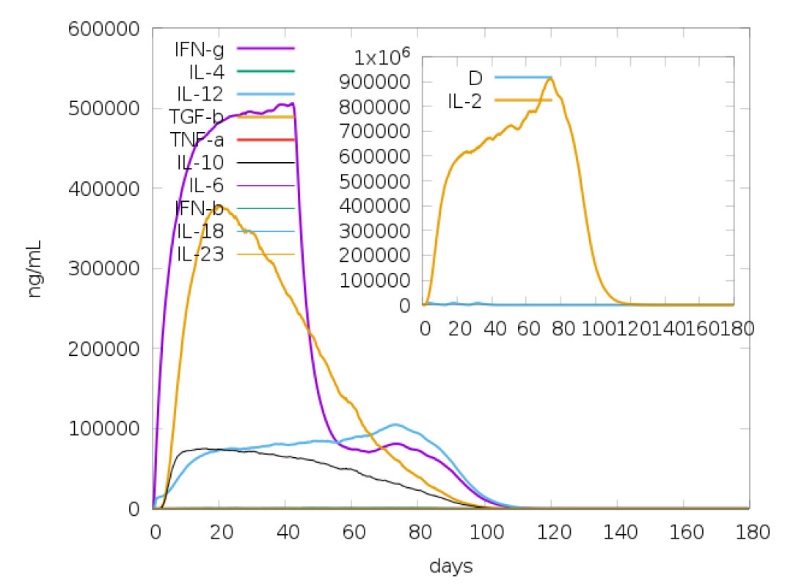


**Supplementary Figure S1-43**. Concentration of cytokines and interleukins analyzed by C-ImmSim online server. Gp63_159-173_ epitope without any adjuvant was injected for three times at intervals of 2 weeks. IFN-gamma (IFN-γ), TGF-b (TGF-β), IL-10, and IL-12 are shown in purple line, yellow thick line, black line, and blue thick line respectively. IL-2 and danger signal (D) are presented in yellow thick line and blue thick line in the insert plot respectively.

**Gp63_203-217_:**


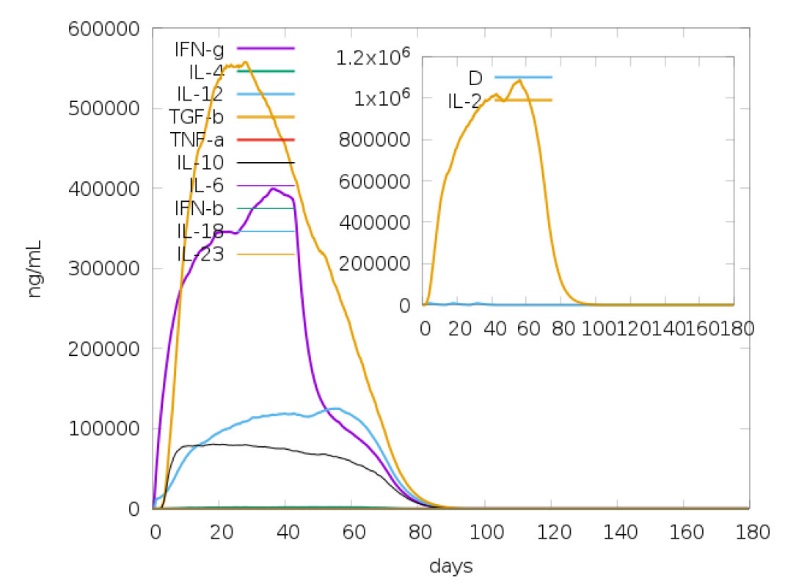


**Supplementary Figure S1-44**. Concentration of cytokines and interleukins analyzed by C-ImmSim online server. Gp63_203-217_ epitope without any adjuvant was injected for three times at intervals of 2 weeks. IFN-gamma (IFN-γ), TGF-b (TGF-β), IL-10, and IL-12 are shown in purple line, yellow thick line, black line, and blue thick line respectively. IL-2 and danger signal (D) are presented in yellow thick line and blue thick line in the insert plot respectively.

**Gp63_492-506_:**


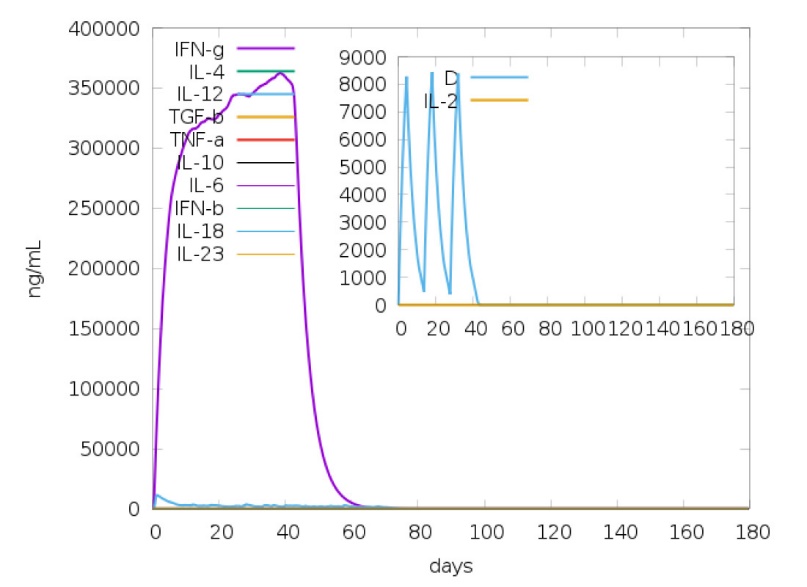


**Supplementary Figure S1-45**. Concentration of cytokines and interleukins analyzed by C-ImmSim online server. Gp63_492-506_ epitope without any adjuvant was injected for three times at intervals of 2 weeks. IFN-gamma (IFN-γ), TGF-b (TGF-β), IL-10, and IL-12 are shown in purple line, yellow thick line, black line, and blue thick line respectively. IL-2 and danger signal (D) are presented in yellow thick line and blue thick line in the insert plot respectively.

**Gp63_158-172_:**

**_
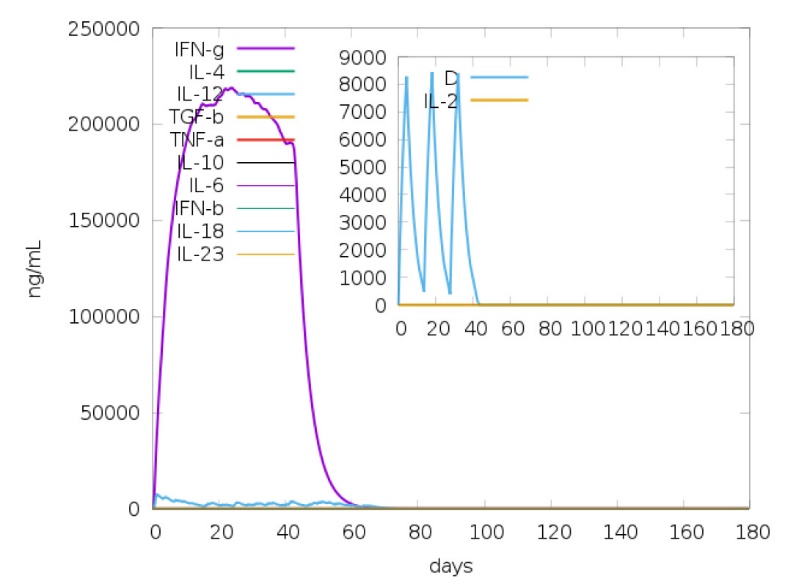
_**

**Supplementary Figure S1-46**. Concentration of cytokines and interleukins analyzed by C-ImmSim online server. Gp63_158-172_ epitope without any adjuvant was injected for three times at intervals of 2 weeks. IFN-gamma (IFN-γ), TGF-b (TGF-β), IL-10, and IL-12 are shown in purple line, yellow thick line, black line, and blue thick line respectively. IL-2 and danger signal (D) are presented in yellow thick line and blue thick line in the insert plot respectively.

**Gp63_107-121_:**


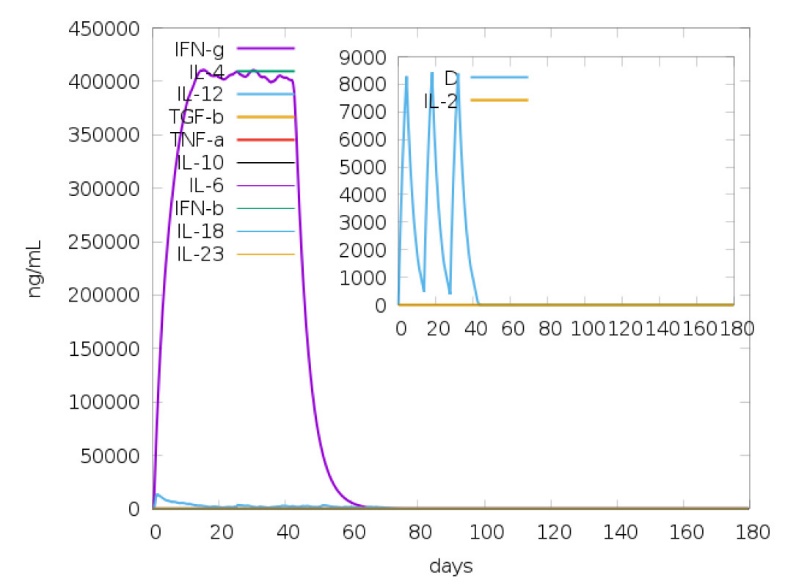


**Supplementary Figure S1-47**. Concentration of cytokines and interleukins analyzed by C-ImmSim online server. Gp63_107-121_ epitope without any adjuvant was injected for three times at intervals of 2 weeks. IFN-gamma (IFN-γ), TGF-b (TGF-β), IL-10, and IL-12 are shown in purple line, yellow thick line, black line, and blue thick line respectively. IL-2 and danger signal (D) are presented in yellow thick line and blue thick line in the insert plot respectively.

**Gp63_491-505_:**

**_
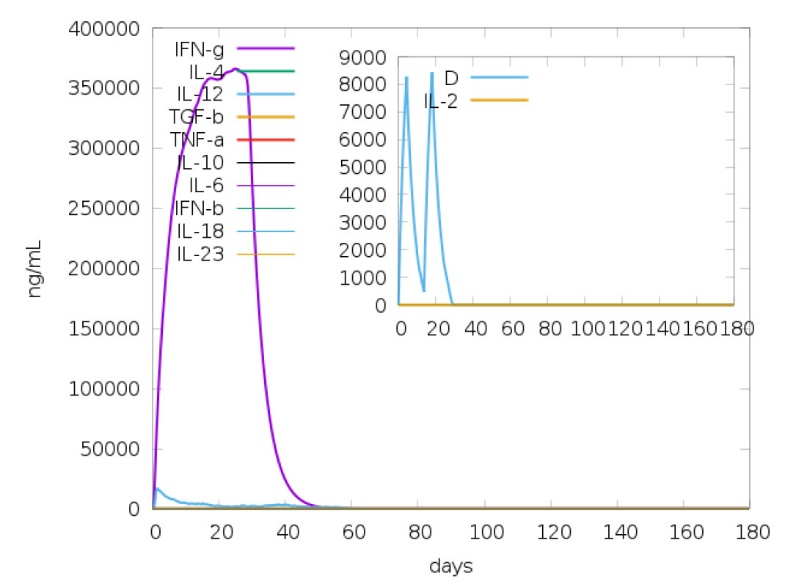
_**

**Supplementary Figure S1-48**. Concentration of cytokines and interleukins analyzed by C-ImmSim online server. Gp63_491-505_ epitope without any adjuvant was injected for three times at intervals of 2 weeks. IFN-gamma (IFN-γ), TGF-b (TGF-β), IL-10, and IL-12 are shown in purple line, yellow thick line, black line, and blue thick line respectively. IL-2 and danger signal (D) are presented in yellow thick line and blue thick line in the insert plot respectively.

**Gp63_341-355_:**

**_
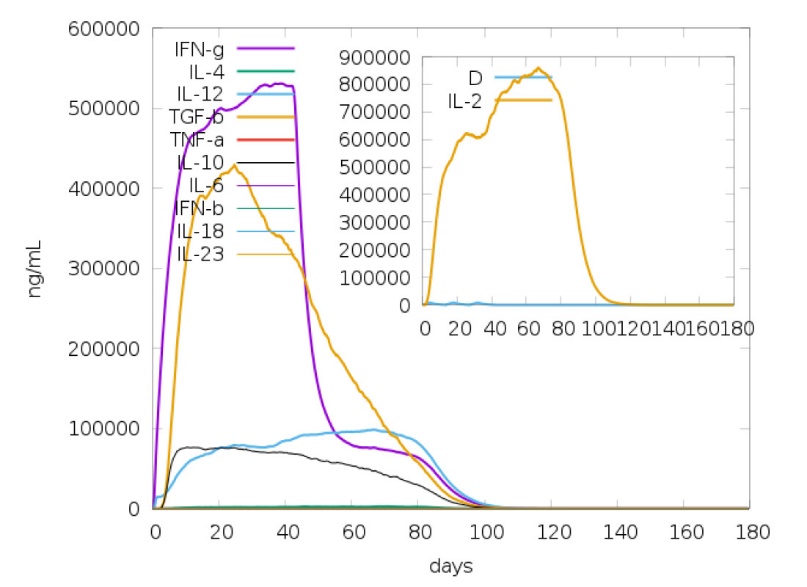
_**

**Supplementary Figure S1-49**. Concentration of cytokines and interleukins analyzed by C-ImmSim online server. Gp63_341-355_ epitope without any adjuvant was injected for three times at intervals of 2 weeks. IFN-gamma (IFN-γ), TGF-b (TGF-β), IL-10, and IL-12 are shown in purple line, yellow thick line, black line, and blue thick line respectively. IL-2 and danger signal (D) are presented in yellow thick line and blue thick line in the insert plot respectively.

**Gp63_277-291_:**

**_
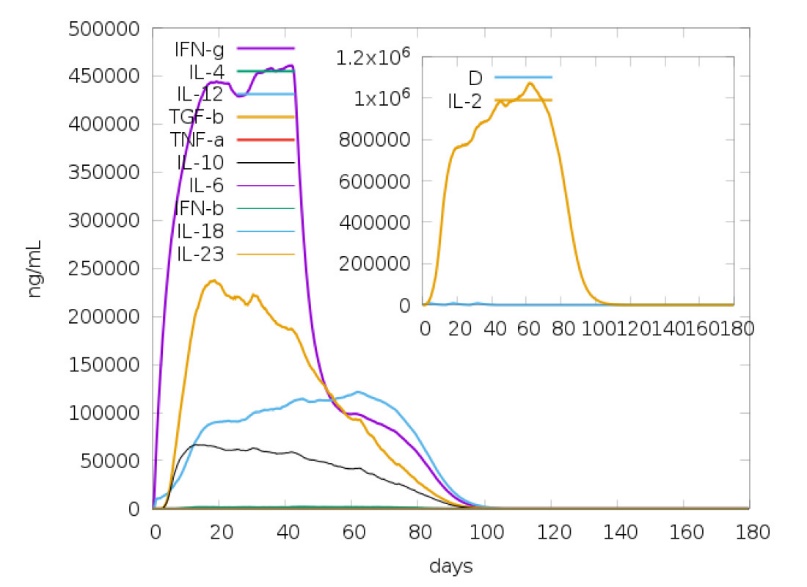
_**

**Supplementary Figure S1-50**. Concentration of cytokines and interleukins analyzed by C-ImmSim online server. Gp63_277-291_ epitope without any adjuvant was injected for three times at intervals of 2 weeks. IFN-gamma (IFN-γ), TGF-b (TGF-β), IL-10, and IL-12 are shown in purple line, yellow thick line, black line, and blue thick line respectively. IL-2 and danger signal (D) are presented in yellow thick line and blue thick line in the insert plot respectively.

**Gp63_279-293_:**

**_
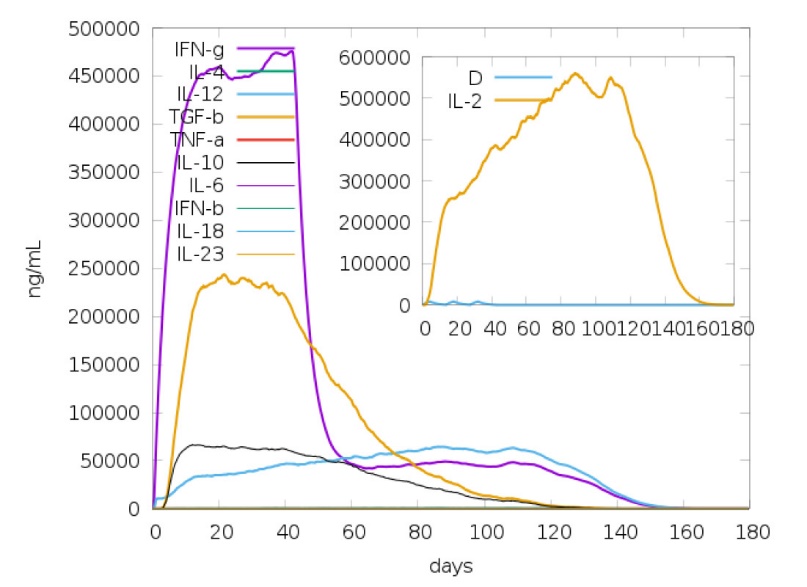
_**

**Supplementary Figure S1-51**. Concentration of cytokines and interleukins analyzed by C-ImmSim online server. Gp63_279-293_ epitope without any adjuvant was injected for three times at intervals of 2 weeks. IFN-gamma (IFN-γ), TGF-b (TGF-β), IL-10, and IL-12 are shown in purple line, yellow thick line, black line, and blue thick line respectively. IL-2 and danger signal (D) are presented in yellow thick line and blue thick line in the insert plot respectively.

**Gp63_160-174_:**

**_
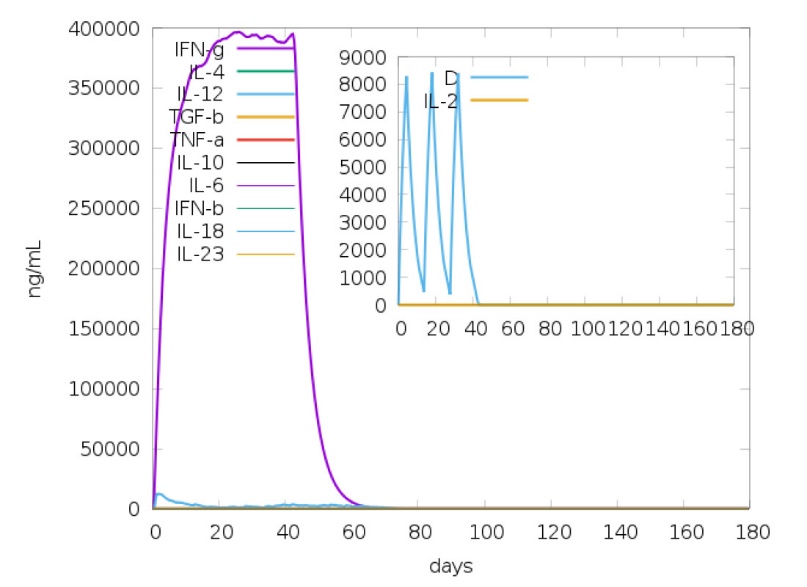
_**

**Supplementary Figure S1-52**. Concentration of cytokines and interleukins analyzed by C-ImmSim online server. Gp63_160-174_ epitope without any adjuvant was injected for three times at intervals of 2 weeks. IFN-gamma (IFN-γ), TGF-b (TGF-β), IL-10, and IL-12 are shown in purple line, yellow thick line, black line, and blue thick line respectively. IL-2 and danger signal (D) are presented in yellow thick line and blue thick line in the insert plot respectively.

**Gp63_197-211_:**

**_
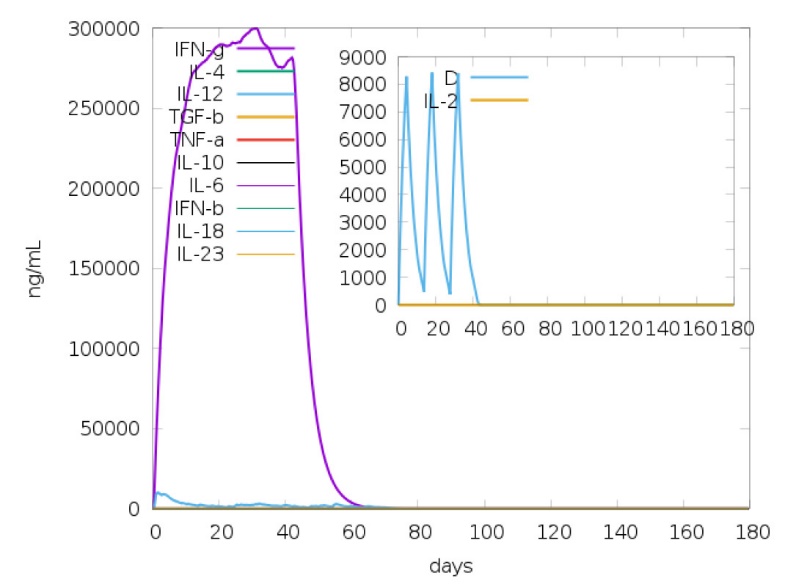
_**

**Supplementary Figure S1-53**. Concentration of cytokines and interleukins analyzed by C-ImmSim online server. Gp63_197-211_ epitope without any adjuvant was injected for three times at intervals of 2 weeks. IFN-gamma (IFN-γ), TGF-b (TGF-β), IL-10, and IL-12 are shown in purple line, yellow thick line, black line, and blue thick line respectively. IL-2 and danger signal (D) are presented in yellow thick line and blue thick line in the insert plot respectively.

**TSA_35-49_:**

**_
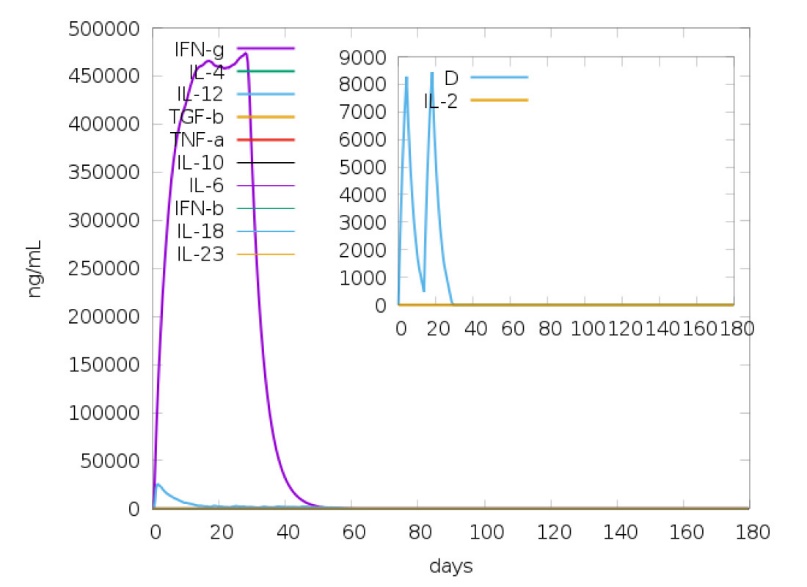
_**

**Supplementary Figure S1-54**. Concentration of cytokines and interleukins analyzed by C-ImmSim online server. TSA_35-49_ epitope without any adjuvant was injected for three times at intervals of 2 weeks. IFN-gamma (IFN-γ), TGF-b (TGF-β), IL-10, and IL-12 are shown in purple line, yellow thick line, black line, and blue thick line respectively. IL-2 and danger signal (D) are presented in yellow thick line and blue thick line in the insert plot respectively.

**TSA_34-48_:**

**_
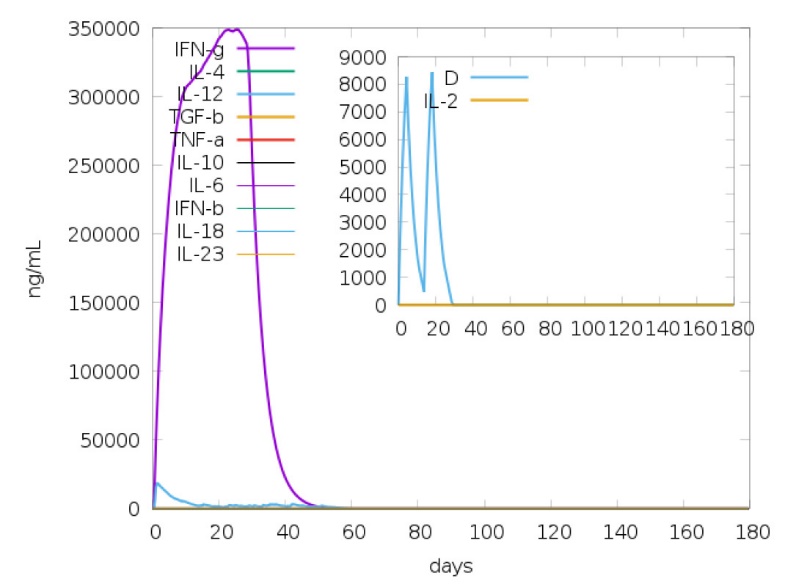
_**

**Supplementary Figure S1-55**. Concentration of cytokines and interleukins analyzed by C-ImmSim online server. TSA_34-48_ epitope without any adjuvant was injected for three times at intervals of 2 weeks. IFN-gamma (IFN-γ), TGF-b (TGF-β), IL-10, and IL-12 are shown in purple line, yellow thick line, black line, and blue thick line respectively. IL-2 and danger signal (D) are presented in yellow thick line and blue thick line in the insert plot respectively.

**TSA_181-195_:**


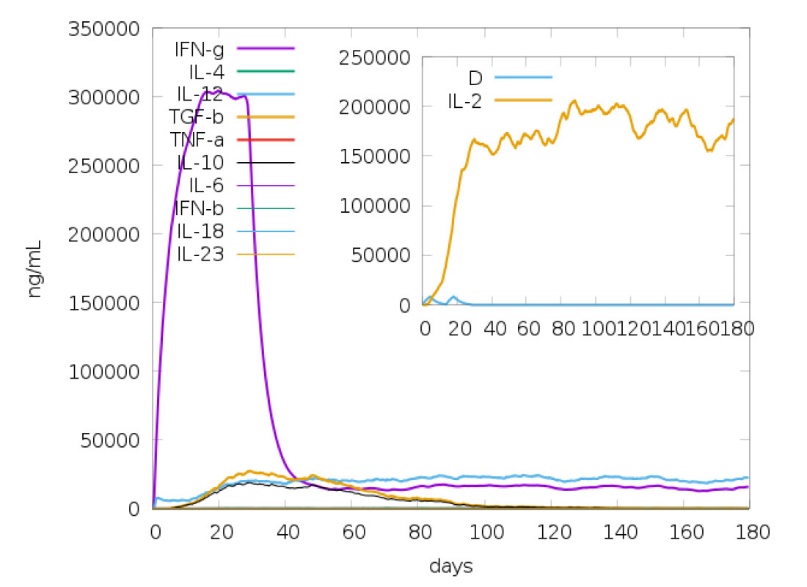


**Supplementary Figure S1-56**. Concentration of cytokines and interleukins analyzed by C-ImmSim online server. TSA_181-195_ epitope without any adjuvant was injected for three times at intervals of 2 weeks. IFN-gamma (IFN-γ), TGF-b (TGF-β), IL-10, and IL-12 are shown in purple line, yellow thick line, black line, and blue thick line respectively. IL-2 and danger signal (D) are presented in yellow thick line and blue thick line in the insert plot respectively.

**TSA_185-199_:**


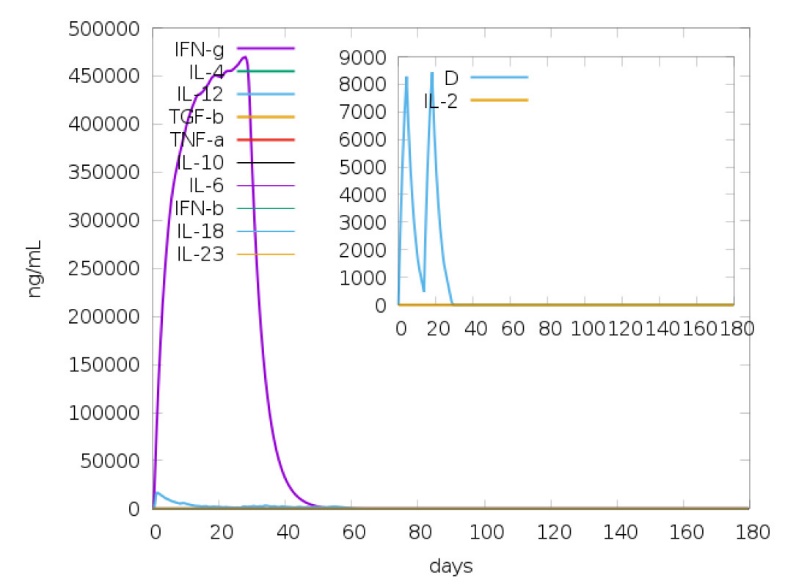


**Supplementary Figure S1-57**. Concentration of cytokines and interleukins analyzed by C-ImmSim online server. TSA_185-199_ epitope without any adjuvant was injected for three times at intervals of 2 weeks. IFN-gamma (IFN-γ), TGF-b (TGF-β), IL-10, and IL-12 are shown in purple line, yellow thick line, black line, and blue thick line respectively. IL-2 and danger signal (D) are presented in yellow thick line and blue thick line in the insert plot respectively.

**TSA_180-194_:**

**_
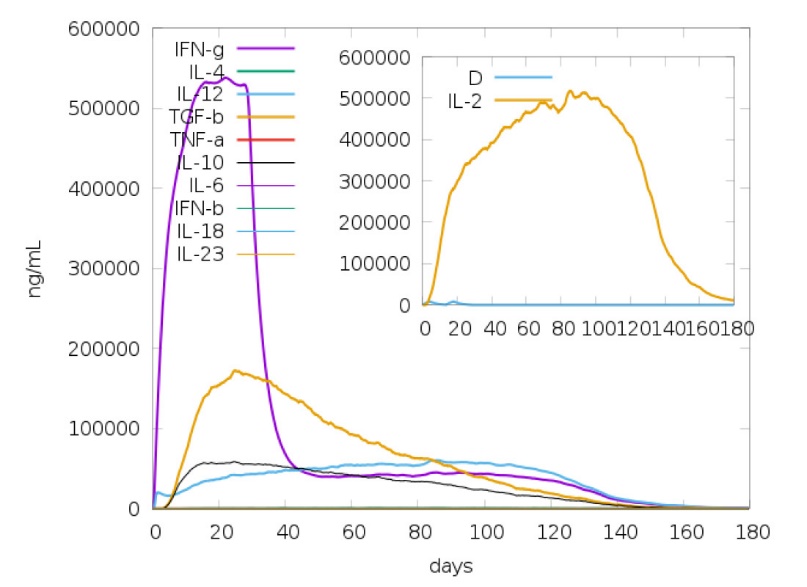
_**

**Supplementary Figure S1-58**. Concentration of cytokines and interleukins analyzed by C-ImmSim online server. TSA_180-194_ epitope without any adjuvant was injected for three times at intervals of 2 weeks. IFN-gamma (IFN-γ), TGF-b (TGF-β), IL-10, and IL-12 are shown in purple line, yellow thick line, black line, and blue thick line respectively. IL-2 and danger signal (D) are presented in yellow thick line and blue thick line in the insert plot respectively.

**TSA_184-198_:**

**_
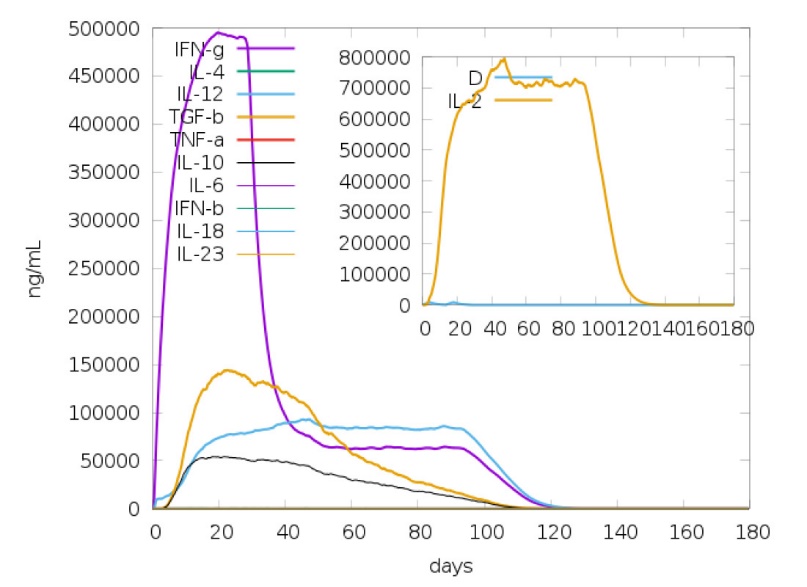
_**

**Supplementary Figure S1-59**. Concentration of cytokines and interleukins analyzed by C-ImmSim online server. TSA_184-198_ epitope without any adjuvant was injected for three times at intervals of 2 weeks. IFN-gamma (IFN-γ), TGF-b (TGF-β), IL-10, and IL-12 are shown in purple line, yellow thick line, black line, and blue thick line respectively. IL-2 and danger signal (D) are presented in yellow thick line and blue thick line in the insert plot respectively.

**TSA_36-50_:**

**_
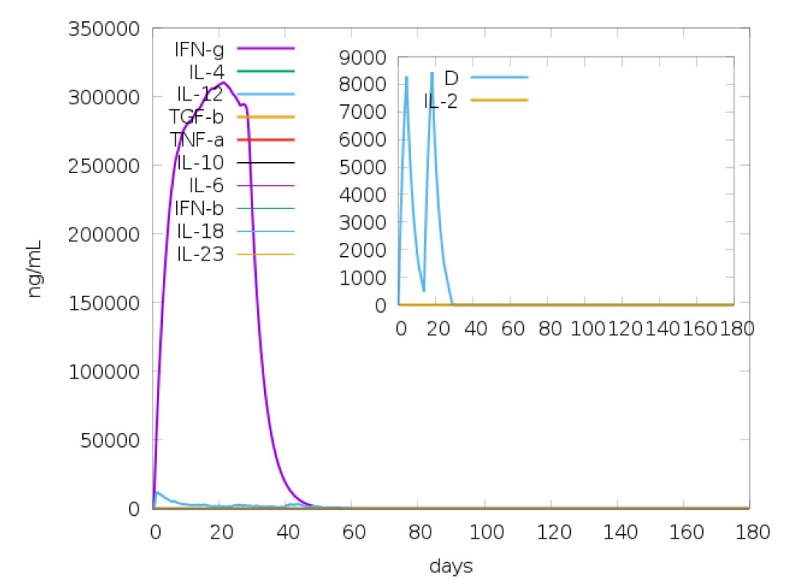
_**

**Supplementary Figure S1-60**. Concentration of cytokines and interleukins analyzed by C-ImmSim online server. TSA_36-50_ epitope without any adjuvant was injected for three times at intervals of 2 weeks. IFN-gamma (IFN-γ), TGF-b (TGF-β), IL-10, and IL-12 are shown in purple line, yellow thick line, black line, and blue thick line respectively. IL-2 and danger signal (D) are presented in yellow thick line and blue thick line in the insert plot respectively.

**SMT_241-255_:**

**_
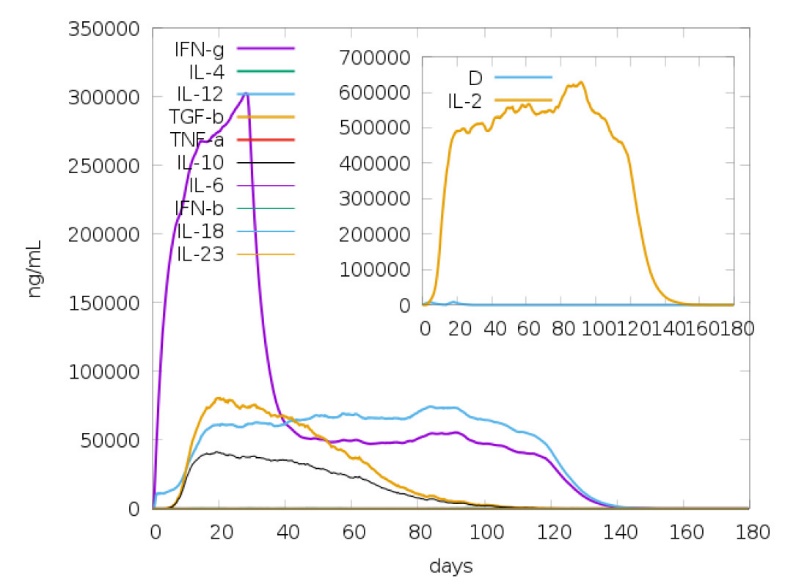
_**

**Supplementary Figure S1-61**. Concentration of cytokines and interleukins analyzed by C-ImmSim online server. SMT_241-255_ epitope without any adjuvant was injected for three times at intervals of 2 weeks. IFN-gamma (IFN-γ), TGF-b (TGF-β), IL-10, and IL-12 are shown in purple line, yellow thick line, black line, and blue thick line respectively. IL-2 and danger signal (D) are presented in yellow thick line and blue thick line in the insert plot respectively.

**SMT_339-353_:**


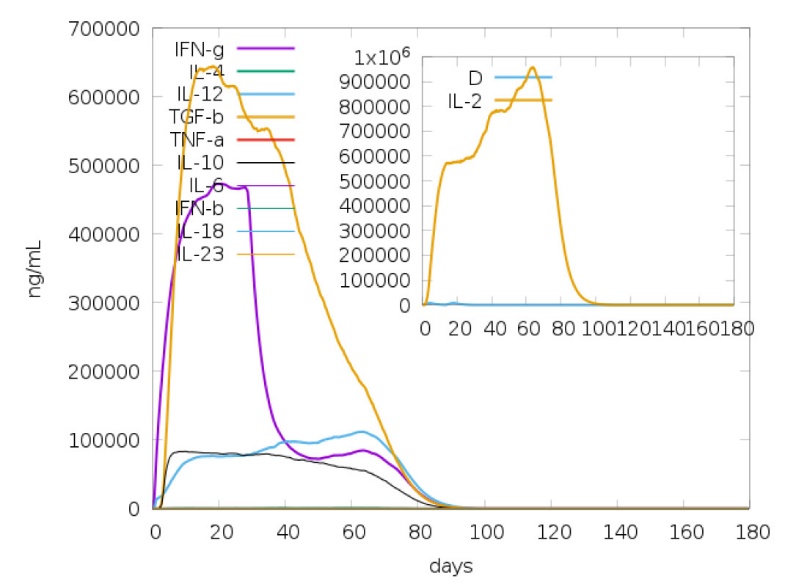


**Supplementary Figure S1-62**. Concentration of cytokines and interleukins analyzed by C-ImmSim online server. SMT_339-353_ epitope without any adjuvant was injected for three times at intervals of 2 weeks. IFN-gamma (IFN-γ), TGF-b (TGF-β), IL-10, and IL-12 are shown in purple line, yellow thick line, black line, and blue thick line respectively. IL-2 and danger signal (D) are presented in yellow thick line and blue thick line in the insert plot respectively.

**SMT_338-352_:**


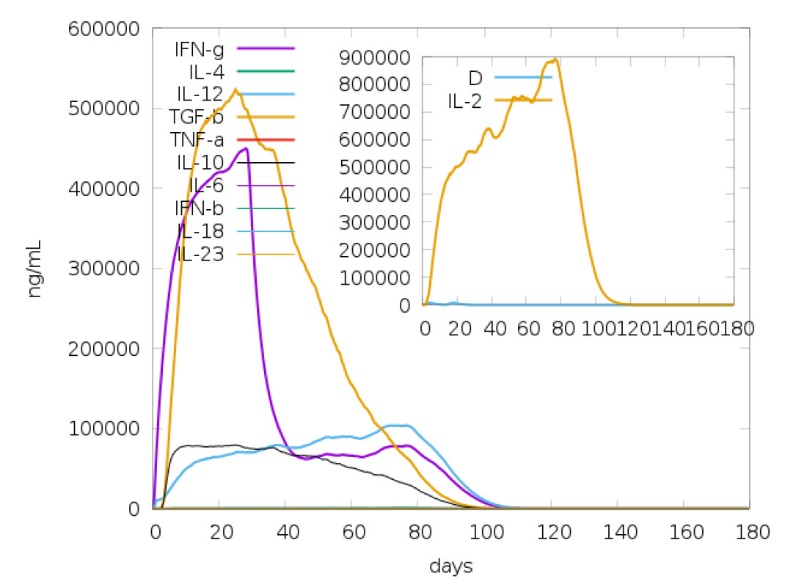


**Supplementary Figure S1-63**. Concentration of cytokines and interleukins analyzed by C-ImmSim online server. SMT_338-352_ epitope without any adjuvant was injected for three times at intervals of 2 weeks. IFN-gamma (IFN-γ), TGF-b (TGF-β), IL-10, and IL-12 are shown in purple line, yellow thick line, black line, and blue thick line respectively. IL-2 and danger signal (D) are presented in yellow thick line and blue thick line in the insert plot respectively.

**SMT_90-104_:**


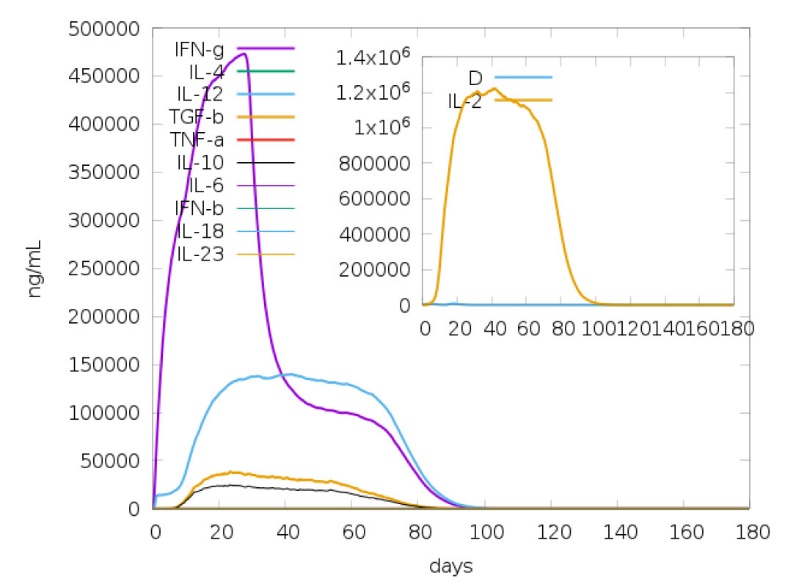


**Supplementary Figure S1-64**. Concentration of cytokines and interleukins analyzed by C-ImmSim online server. SMT_90-104_ epitope without any adjuvant was injected for three times at intervals of 2 weeks. IFN-gamma (IFN-γ), TGF-b (TGF-β), IL-10, and IL-12 are shown in purple line, yellow thick line, black line, and blue thick line respectively. IL-2 and danger signal (D) are presented in yellow thick line and blue thick line in the insert plot respectively.

**SMT_246-260_:**

**_
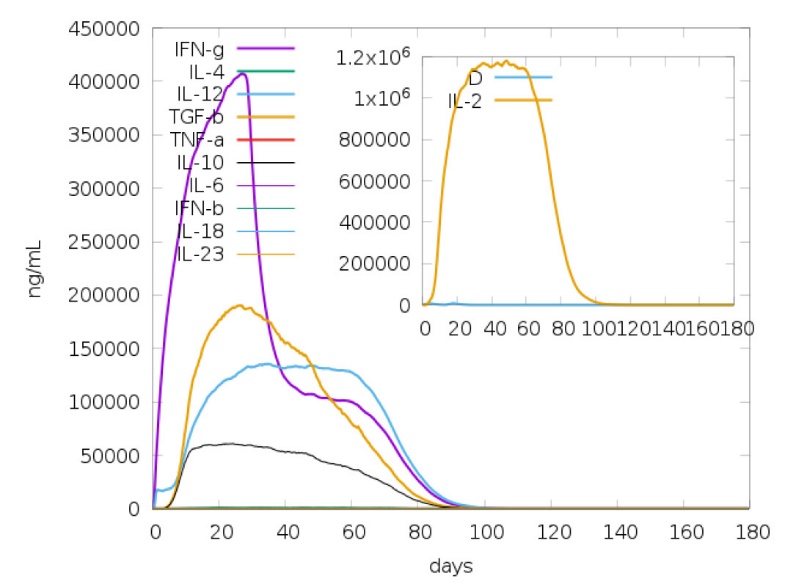
_**

**Supplementary Figure S1-65**. Concentration of cytokines and interleukins analyzed by C-ImmSim online server. SMT_246-260_ epitope without any adjuvant was injected for three times at intervals of 2 weeks. IFN-gamma (IFN-γ), TGF-b (TGF-β), IL-10, and IL-12 are shown in purple line, yellow thick line, black line, and blue thick line respectively. IL-2 and danger signal (D) are presented in yellow thick line and blue thick line in the insert plot respectively.

**SMT_334-348_:**

**_
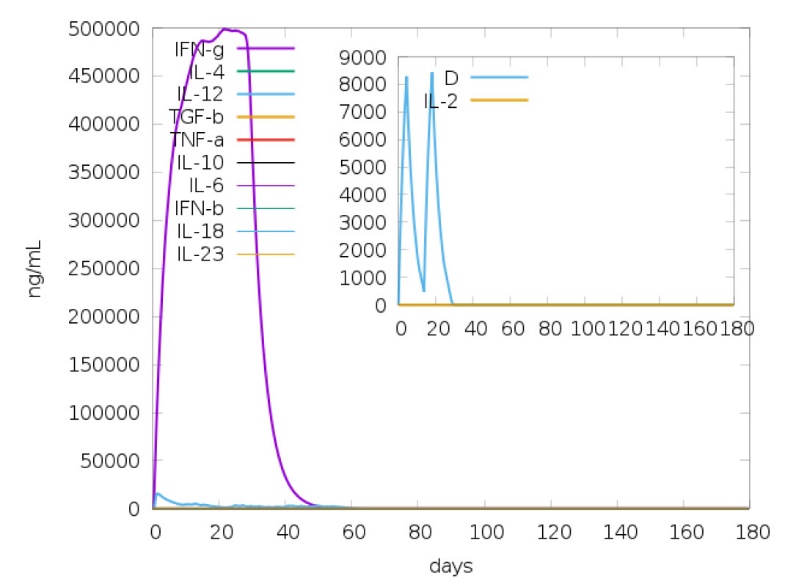
_**

**Supplementary Figure S1-66**. Concentration of cytokines and interleukins analyzed by C-ImmSim online server. SMT_334-348_ epitope without any adjuvant was injected for three times at intervals of 2 weeks. IFN-gamma (IFN-γ), TGF-b (TGF-β), IL-10, and IL-12 are shown in purple line, yellow thick line, black line, and blue thick line respectively. IL-2 and danger signal (D) are presented in yellow thick line and blue thick line in the insert plot respectively.

**SMT_89-103_:**

**_
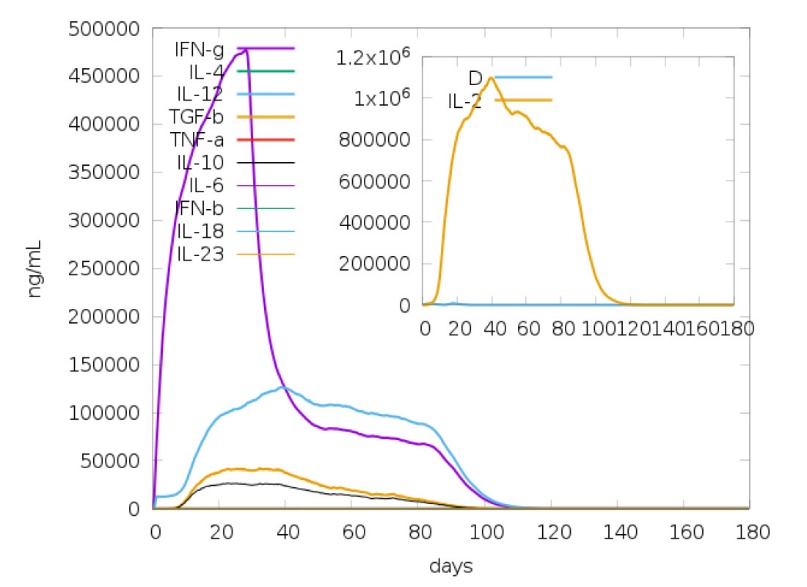
_**

**Supplementary Figure S1-67**. Concentration of cytokines and interleukins analyzed by C-ImmSim online server. SMT_89-103_ epitope without any adjuvant was injected for three times at intervals of 2 weeks. IFN-gamma (IFN-γ), TGF-b (TGF-β), IL-10, and IL-12 are shown in purple line, yellow thick line, black line, and blue thick line respectively. IL-2 and danger signal (D) are presented in yellow thick line and blue thick line in the insert plot respectively.

**SMT_271-285_:**


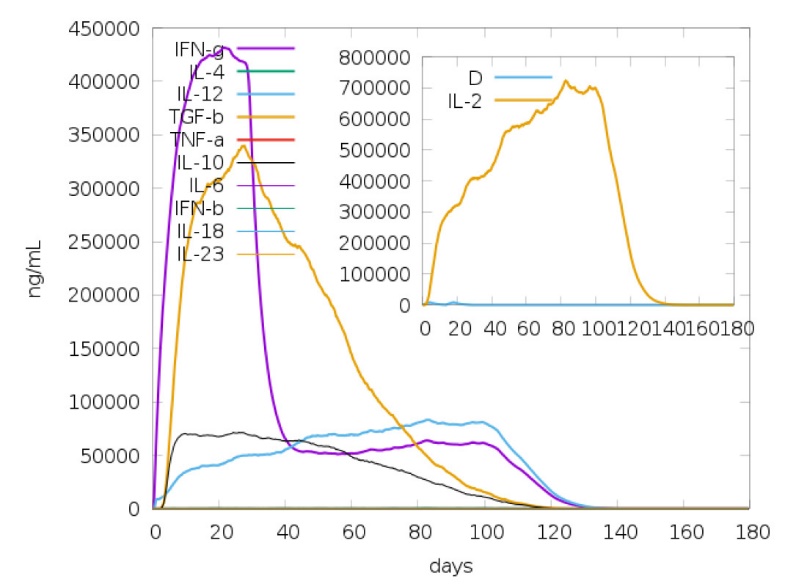


**Supplementary Figure S1-68**. Concentration of cytokines and interleukins analyzed by C-ImmSim online server. SMT_271-285_ epitope without any adjuvant was injected for three times at intervals of 2 weeks. IFN-gamma (IFN-γ), TGF-b (TGF-β), IL-10, and IL-12 are shown in purple line, yellow thick line, black line, and blue thick line respectively. IL-2 and danger signal (D) are presented in yellow thick line and blue thick line in the insert plot respectively.

**Supplementary Figure S2: The distribution of CTL epitopes based on percentile rank and antigenicity**

CTL epitopes with antigenicity index >0.5, non-toxin and non-allergen, deriving from HbR, Kmp-11, Gp63, TSA, and SMT were analyzed their distribution based on percentile rank and antigenicity. The results of distribution are shown as folloiw:

**HbR**

**
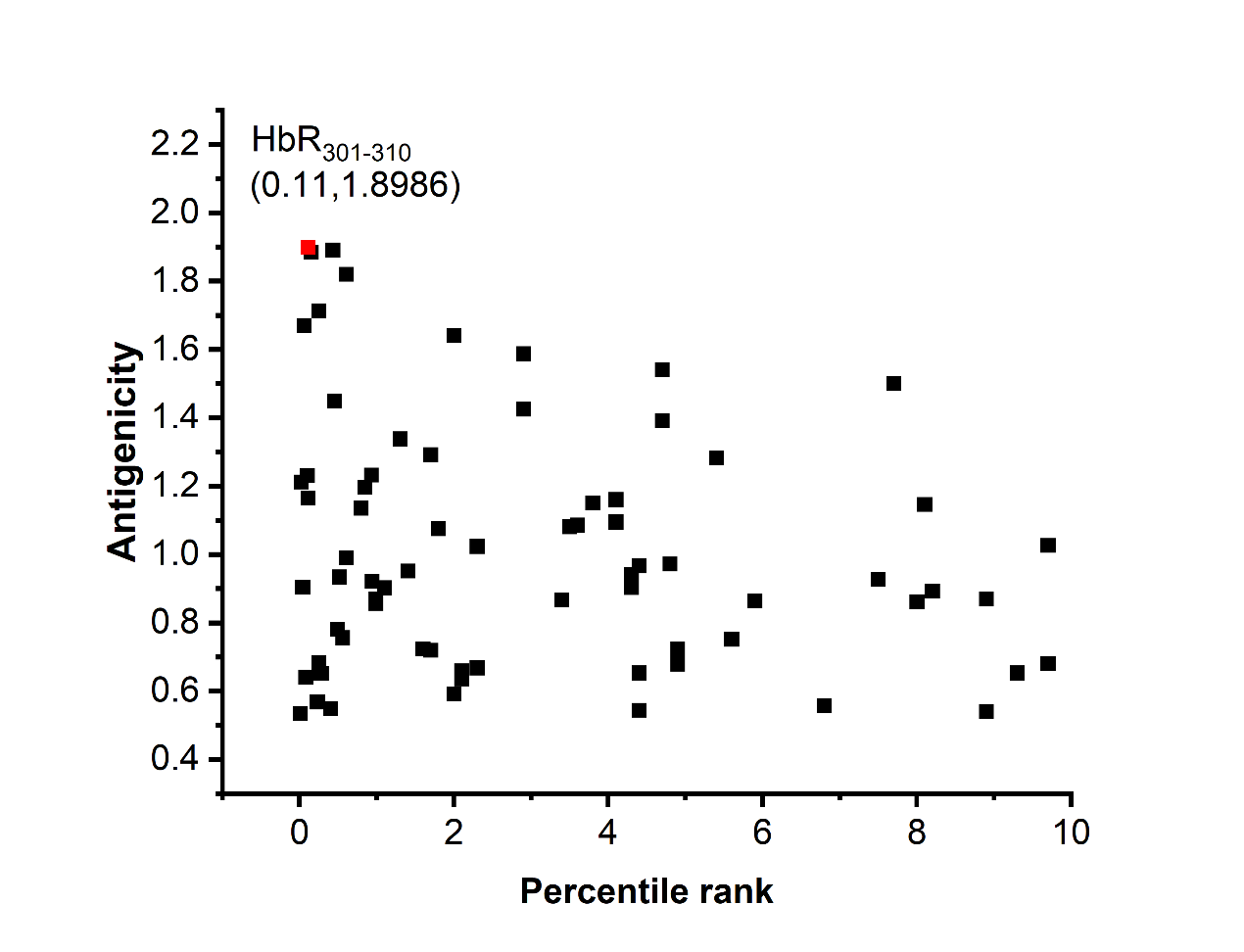
**

**Supplementary Figure S2-1.** CTL epitopes from HbR were distributed according their percentile rank and antigenicity. HbR_301-310_ epitope that has simultaneously low percentile rank and large antigenicity index was colored with red and selected for multi-epitope vaccine construction.

**KMP-11**

**
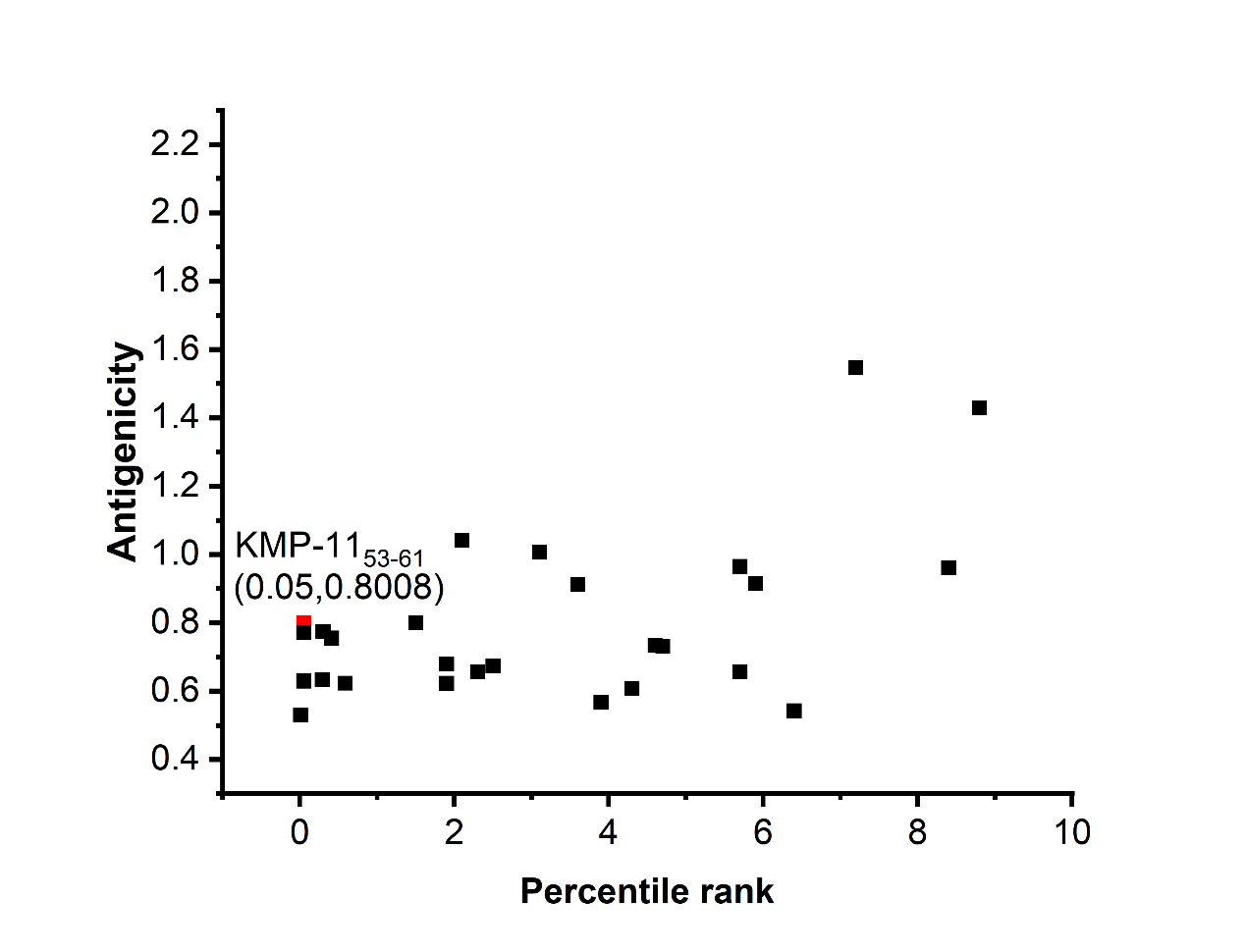
**

**Supplementary Figure S2-2.** CTL epitopes from KMP-11 were distributed according their percentile rank and antigenicity. Kmp-11_53-61_ epitope that has simultaneously low percentile rank and large antigenicity index was colored with red and selected for multi-epitope vaccine construction.

**Gp63**

**
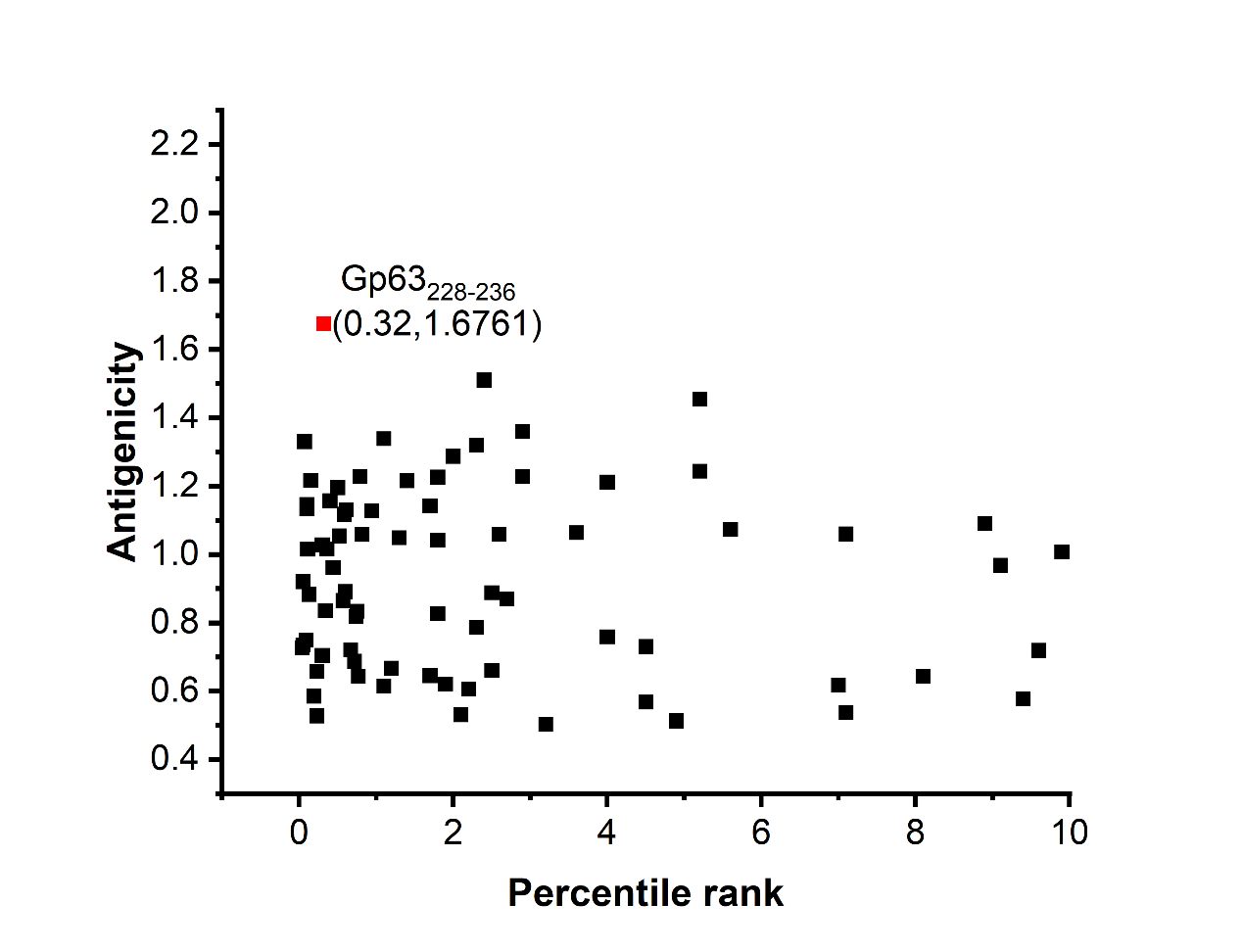
**

**Supplementary Figure S2-3.** CTL epitopes from Gp63 were distributed according their percentile rank and antigenicity. Gp63_228-236_ epitope that has simultaneously low percentile rank and large antigenicity index was colored with red and selected for multi-epitope vaccine construction.

**TSA**_
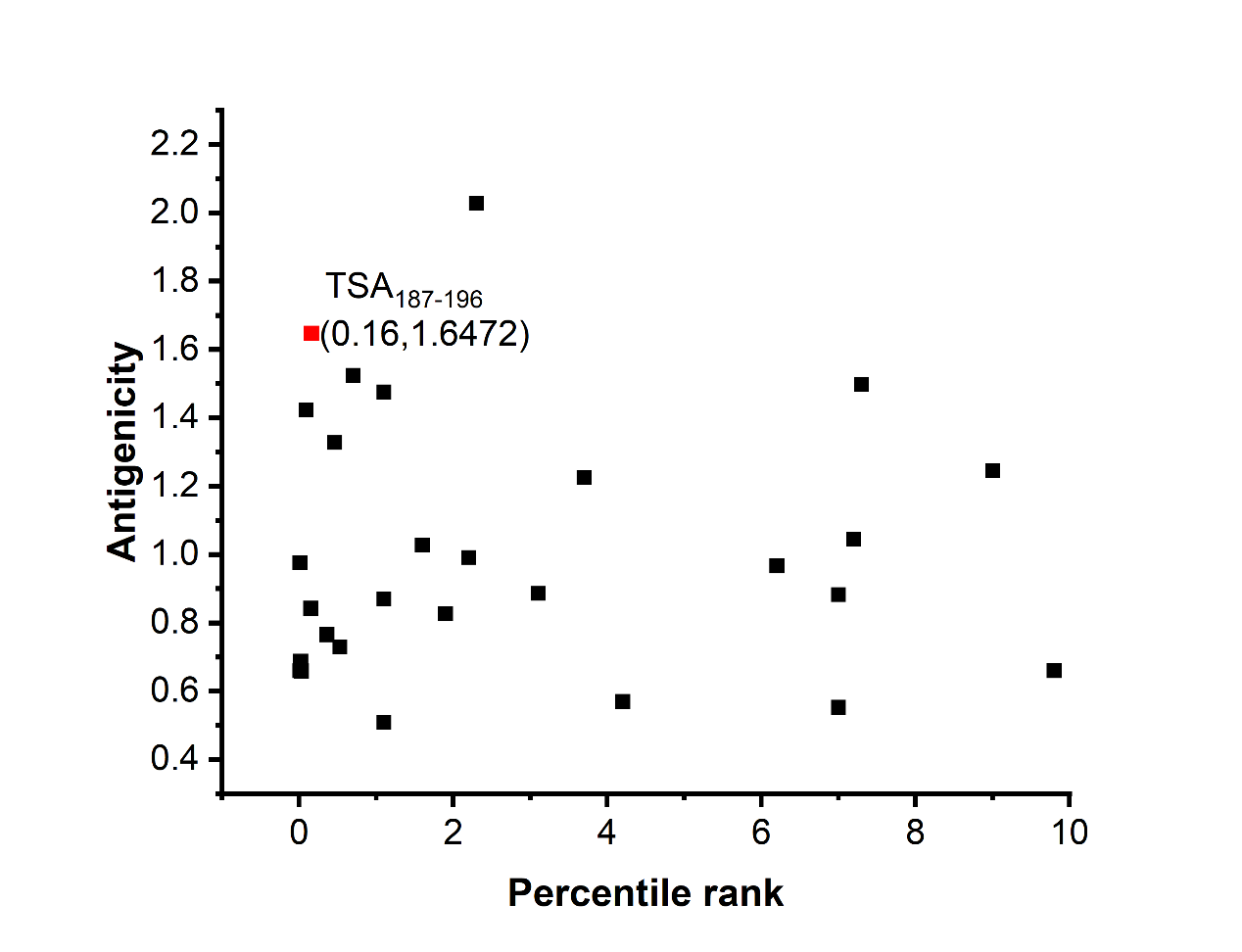
_

**Supplementary Figure S2-4.** CTL epitopes from TSA were distributed according their percentile rank and antigenicity. TSA_187-196_ epitope that has simultaneously low percentile rank and large antigenicity index was colored with red and selected for multi-epitope vaccine construction.

**SMT
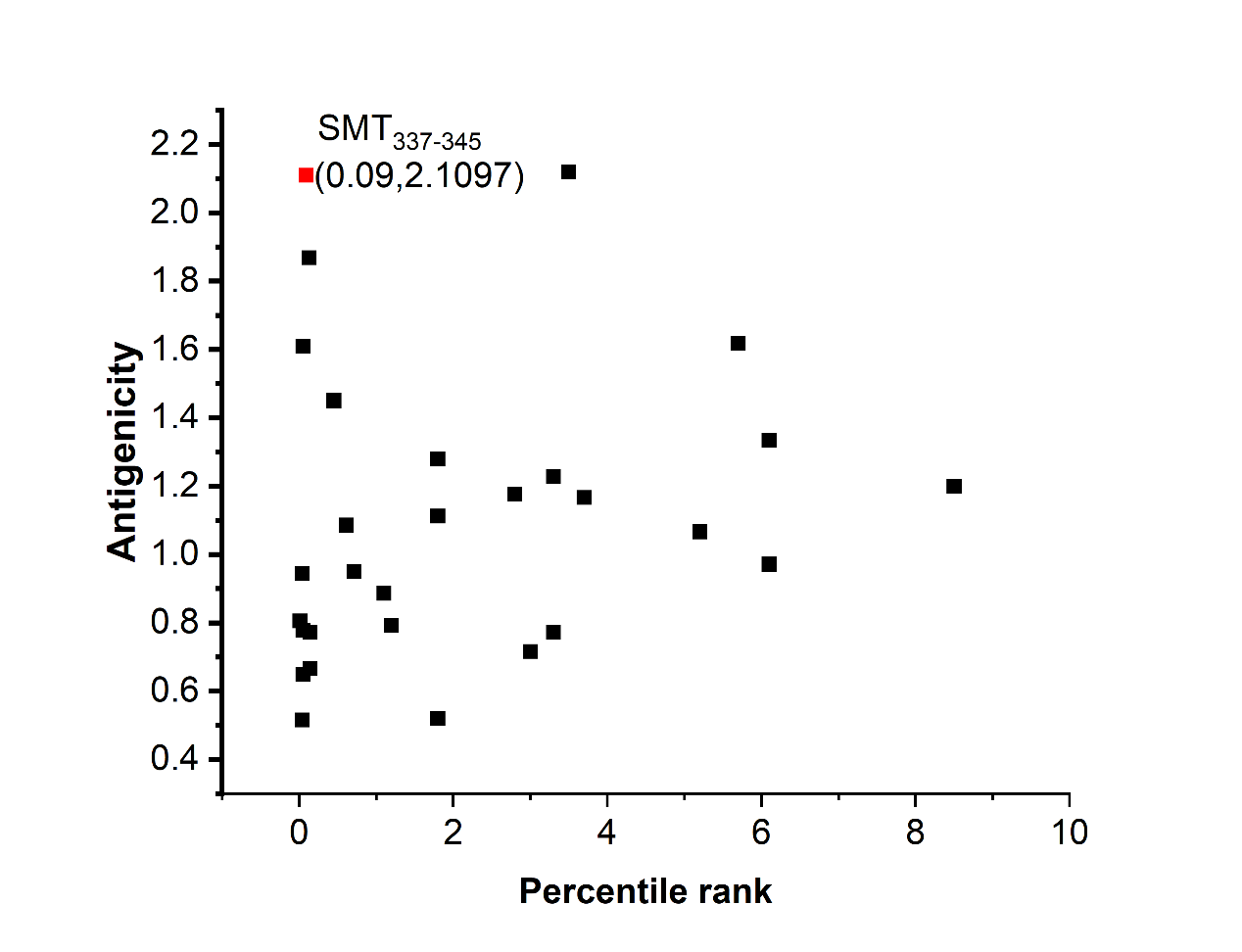
**

**Supplementary Figure S2-5.** CTL epitopes from SMT were distributed according their percentile rank and antigenicity. SMT_337-345_ epitope that has simultaneously low percentile rank and large antigenicity index was colored with red and selected for multi-epitope vaccine construction.
